# Mapping black panthers: Macroecological modeling of melanism in leopards (*Panthera pardus*)

**DOI:** 10.1371/journal.pone.0170378

**Published:** 2017-04-05

**Authors:** Lucas G. da Silva, Kae Kawanishi, Philipp Henschel, Andrew Kittle, Arezoo Sanei, Alexander Reebin, Dale Miquelle, Andrew B. Stein, Anjali Watson, Laurence Bruce Kekule, Ricardo B. Machado, Eduardo Eizirik

**Affiliations:** 1 PUCRS, Faculdade de Biociências, Porto Alegre, Rio Grande do Sul, Brazil; 2 Malaysian Conservation Alliance for Tigers, Petaling Jaya, Malaysia; 3 Panthera - Lion Program, New York, New York, United States of America; 4 Wilderness & Wildlife Conservation Trust, Colombo, Sri Lanka; 5 Asian Leopard Specialist Society, Tehran, Iran; 6 Wildlife Conservation Society, New York, New York, United States of America; 7 Landmark College, Putney, Vermont, United States of America; 8 Botswana Predator Conservation Trust, Maun, Botswana; 9 BK Wildlife Photography Co., Bangkok, Thailand; 10 Universidade de Brasília, Brasília, Distrito Federal, Brazil; 11 Instituto Pró-Carnívoros, Atibaia, São Paulo, Brazil; Texas A&M University College Station, UNITED STATES

## Abstract

The geographic distribution and habitat association of most mammalian polymorphic phenotypes are still poorly known, hampering assessments of their adaptive significance. Even in the case of the black panther, an iconic melanistic variant of the leopard (*Panthera pardus*), no map exists describing its distribution. We constructed a large database of verified records sampled across the species’ range, and used it to map the geographic occurrence of melanism. We then estimated the potential distribution of melanistic and non-melanistic leopards using niche-modeling algorithms. The overall frequency of melanism was *ca*. 11%, with a significantly non-random spatial distribution. Distinct habitat types presented significantly different frequencies of melanism, which increased in Asian moist forests and approached zero across most open/dry biomes. Niche modeling indicated that the potential distributions of the two phenotypes were distinct, with significant differences in habitat suitability and rejection of niche equivalency between them. We conclude that melanism in leopards is strongly affected by natural selection, likely driven by efficacy of camouflage and/or thermoregulation in different habitats, along with an effect of moisture that goes beyond its influence on vegetation type. Our results support classical hypotheses of adaptive coloration in animals (*e*.*g*. Gloger’s rule), and open up new avenues for in-depth evolutionary analyses of melanism in mammals.

## Introduction

Animal coloration has often been proposed to possess adaptive relevance, performing various roles in behavioral and ecological processes [1–8]. However, to this date remarkably little is known about the evolutionary and ecological significance of most coloration phenotypes across all groups of animals, including variants that are easily and commonly observed in the field. In many cases, even the geographic distribution of coloration variants remains poorly documented, precluding a more in-depth analysis of their relationships with ecological variables.

A very commonly observed coloration variant is melanism, which consists of a darkened external pigmentation relative to what would be considered a ‘normal’ or ‘wild-type’ phenotype. Several biological factors, such as thermoregulation, camouflage, aposematism, susceptibility or response to disease, sexual selection and reproductive success, have been classically associated with melanism in various groups of organisms [6; 9–11]. There are classical hypotheses, dating back to the 19^th^ century, which postulate an adaptive role for melanism (*e*.*g*. [1–2]), suggesting an association between dark individuals and wetter areas with dense vegetation (*i*.*e*. tropical forests). In addition, previous studies have mentioned the possibility that selection against dark individuals might operate in open areas, where solar radiation and mean temperatures are high [1; 10–11].

The occurrence of melanism is common in the cat family (Felidae), having been documented in 13 of its 37 species, and having arisen independently at least eight times in the family [12–14]. Interestingly, this variant phenotype seems to always be maintained as a polymorphism, never reaching species-wide fixation in any felid. Of all the wild cats exhibiting this phenotype, perhaps the most widely known is the black panther, a melanistic form of the leopard [*Panthera pardus* (Linnaeus, 1758)]. Although the molecular basis of melanism in this species has already been identified as a recessively inherited mutation in the *ASIP* (*Agouti Signaling Protein*) gene [13], the understanding of its adaptive relevance remains in its infancy.

Given the very broad geographic distribution of leopards (from the Russian Far East to Africa), encompassing a diverse array of habitats, from deserts to rainforests, and from the humid tropics to temperate zones [15–16], a necessary first step is to map the occurrence of melanism across their range. Recent analyses have indicated that melanism can reach very high frequencies in some leopard populations (*e*.*g*. Southeast Asia [17–18]). In addition, there have been confirmed reports of melanistic leopards in India [15; 19–20], Ethiopia [15; 21], Java and Malaysia [17–18; 22–25], Aberdare Mountains in Kenya [15] and Nepal [26], as well as a potential occurrence in South Africa [15; 27]. Although these initial observations may suggest the hypothesis that leopard melanism provides an adaptive advantage in certain ecological conditions [6; 12–13; 28–29], they are still localized, and have so far not been analyzed systematically. Moreover, although at least four of the leopard subspecies suggested by [30] have been cited in the literature as having confirmed records of melanism, the exact geographic range of this coloration phenotype has never been mapped in any of them, nor in leopards as a whole.

Therefore, we set out to investigate the distribution of melanism in leopards, and to conduct a systematic test of its association with different habitats. In addition, we aimed to generate and assess spatial distribution models constructed for the different phenotypes. Although some distribution models have been generated for leopards on smaller geographic scales [31–33], no study has yet produced a model for its entire range, nor focused on spatial patterns of phenotypic variation in this species. Therefore, we generated separate, range-wide models for melanistic and non-melanistic leopards, and used them to investigate the geographic distribution and underlying ecological associations of melanism in this felid. We employed this approach to test two alternative hypotheses: (1) melanism is present throughout the species distribution, occurring randomly in all environments (*i*.*e*. lack of association between melanism and different habitats); and (2) melanism is distributed non-randomly, and its presence is associated with particular habitats and environmental parameters. Testing these hypotheses led to novel insights into the ecological and evolutionary processes affecting melanism in this wide-ranging species.

## Methods

### Species data and frequency-based analyses

**Fig 1:**
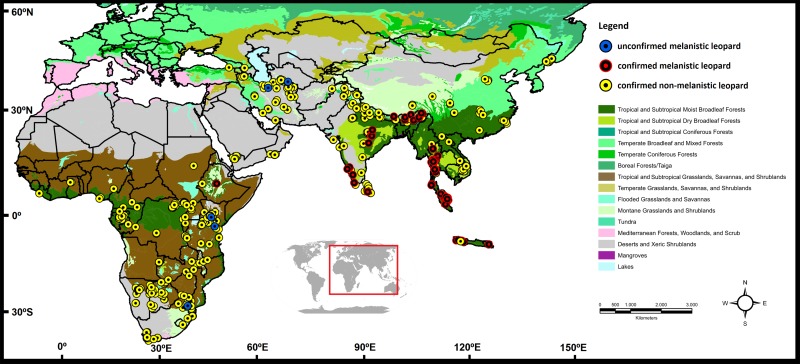
Location of melanistic and non-melanistic leopard records analyzed in this study, overlaid on a map of terrestrial biomes (based on [35]).

We constructed a database of location records (Fig 1; S1 Table), spanning the entire historical range of the leopard from the Russian Far East to Africa, and representing a very broad suite of biomes (montane, mediterranean, temperate, mixed, moist and dry forests, grasslands, savannahs, tundra, woodlands, scrublands and deserts). These records were obtained from four different sources: (1) specimens held in scientific collections that possessed precise information on their geographic origin, as well as coat color data (preferably individuals whose color could be directly ascertained and photographed); (2) individuals found dead or captured during field studies; (3) camera-trap data (with only individuals visibly identified as unique being counted, when sampled from the same location [defined by a 25-km-diameter buffer to minimize the chance of double-counting, based on the maximum reported home range size for the species by [15]]); and (4) direct communication by reliable field researchers or published bibliographical sources.

The data set assembled in this study was completely derived from information collected by the authors and their collaborators in the context of previous studies (*e*.*g*. field ecology surveys), as well as publicly available records (*e*.*g*. details on all museum specimens are available online at each institution's website). A list of all records and their sources is shown in S1 Table. No animal was captured nor handled for the purpose of this study. No biological material was collected nor transported internationally, precluding the need for any collection or export/import permit.

We performed an initial assessment of phenotype frequency and distribution using all location records of individuals confirmed to be melanistic or non-melanistic. We also constructed a global map depicting the distribution of these coloration phenotypes, incorporating the confirmed records and also including four points in which the presence of melanism was reported by not directly documented (referred to herein as ‘unconfirmed records’). For that purpose, we inserted location records of non-melanistic, melanistic and unconfirmed melanistic individuals into an ArcGis 9.3 [34] database. All sampled locations were converted into degree coordinates with the *WGS84* map datum. Additionally, we used a biome layer following the classification into terrestrial ecoregions [35], to extract and analyze information about the habitats where the different phenotypes were recorded.

As there were no systematic biases in our record acquisition with respect to coloration phenotype (*i*.*e*. sampling was random with regard to coat color, with differences among data types [*e*.*g*. museum pelts *vs*. camera-trap photos] not affecting the overall estimate — see Results), we assumed that the presence of melanism in the total data set represented the global frequency in the species. This provided a null hypothesis against which we tested potential deviations in different regions, using a chi-square test.

After this initial analysis, we generated a filtered database to remove potential noise induced by heterogeneous sampling and recent changes in natural landscapes. To do this, we excluded samples collected ≥ 20 years ago, duplicate points (*i*.*e*. different individuals sampled at exactly the same coordinates) and records associated with high rates of landscape modification between 2000 and 2013 (using the annual mean Normalized Difference Vegetation Index [NDVI — see below] as a mask layer and ±0.7 as the cutoff threshold). The filtered database therefore included only recent records that could be directly compared to current landscape features and climate variables, and was employed for all subsequent analyses.

To test whether the spatial distribution of melanism was random throughout the geographic range of leopards, we used a complete spatial randomness (CSR) analysis using a nearest-neighbor method modified to incorporate a multivariate kernel estimation to guide the simulations [36]. This was performed with the 'raster' and 'spatstat' packages in R software [37]. To conduct this test, we initially delimited the spatial scope of analysis by assigning a buffer (~200km radius) around each of our sampled points, and then merging all the included buffers into a single polygon to cover the effectively sampled area (S1 Fig). We then used 500 simulations to test the null hypothesis that our location records were randomly distributed (considering the multivariate kernel probability distribution) within this global polygon, and compared the patterns observed for the melanistic and non-melanistic phenotypes.

### Spatial modeling

To perform in-depth spatial analyses of the two phenotypes (non-melanistic and melanistic), we used the approach proposed by [38] to generate separate potential distribution models for each of them. We used 38 explanatory environmental variables and landscape data: 35 bioclimatic variables obtained from the Worldclim (http://www.worldclim.org) and Climond (http://www.climond.org) databases, a digital elevation model (obtained from the SRTM — http://www2.jpl.nasa.gov/srtm), landscape features (obtained from the ESA GlobCover Project 2009 — http://due.esrin.esa.int/page_globcover.php, and NASA NEO NDVI Modis — http://neo.sci.gsfc.nasa.gov). All variables had ~1 km pixel spatial resolution (S2 Table).

To avoid problems of model overfitting caused by correlation among explanatory variables, we ran Pearson's correlation coefficient test (*r*) for each pair of variables [39–41]. We assessed this correlation by extracting variable information from 10,000 unique and randomly generated points sampled from the currently known geographic distribution of leopards (obtained from IUCN and complemented by our own records) using ArcGis 9.3. We selected 12 predictors that were not highly correlated with each other, using *r* = 0.7 as the cut-off value. These variables were: annual mean temperature, maximum temperature of the warmest week, minimum temperature of the coldest week, annual precipitation, precipitation of the wettest week, precipitation seasonality, annual mean radiation, radiation seasonality, highest weekly moisture index, mean moisture index of the wettest quarter, mean moisture index of the driest quarter, and altitude (see S2 Table and S2 and S3 Figs). We used these 12 selected predictors to analyze the filtered database, assuming that they were adequate and sufficient ecological indicators for niche and biogeographic modeling [42].

We modeled the distribution of non-melanistic and melanistic leopards using the maximum entropy algorithm implemented in the Maxent 3.3.3k software [43–47]. We generated two different models: (1) only non-melanistic individuals (control model) and (2) only melanistic animals (melanism model). For each of these sets of location records, we used 70% of the points for training and 30% for testing the models, with the data being sampled using the bootstrap routine [48]. For all runs, we used the following parameters and configurations: random seed, convergence threshold of 1E-5, 500 iterations and 10,000 hidden background points [49]. The model performance was assessed by the AUC (Area Under Curve) value for the Receiver Operating Characteristic (ROC) curve based on sensitivity *versus* specificity of the response between occurrence data and predictors, incorporating a binomial probability as a null model [48–51]. The Maxent modeling results were converted into ASCII format files and processed with ArcGis 9.3, yielding distribution maps for the two different leopard phenotypes.

To assess potential differences in habitat association between the melanistic and non-melanistic individuals, we used two methods to directly compare niches between the two phenotypes: (1) differences in suitability values between the two models at our own location records; and (2) a statistical assessment of niche equivalency. The former was performed by extracting absolute values of habitat suitability for each of our location records (regardless of the coat color) in each of the two models (*i*.*e*. each point yielded two values). We then employed a paired t-test to compare the mean suitability across all points between the two models, aiming to assess whether any significant difference could be discerned. The latter method employed Shoener's index (*D*), Hellinger’s distance (*I*) and the relative rank (*RR*), as implemented in ENMTools 1.3 [52–54], to test the niche equivalency between the two models, using the average values from the 500 Maxent replicates generated for each phenotype. The resulting indices (*i*.*e*. observed values) were compared against a null distribution that was generated as follows: (1) Based on the proportion of the two phenotypes observed in the overall database (10% melanistic and 90% non-melanistic), we produced 100 random replicates from within the record set of the opposite coat color group (*i*.*e*. 10% of records sampled per replicate from the non-melanistic set; 90% sampled from the melanistic set); (2) For each random replicate, we used the sampled records as the training set in Maxent, while the remaining ones were used as the test set; (3) We performed a reciprocal comparison of the Maxent results (100 replicates based on the melanistic records *vs*. 100 from the non-melanistic ones; *i*.*e*. 10,000 comparisons) that yielded a null distribution of *D*, *I* and *RR* values.

Finally, to investigate which environmental predictors most influenced the differential distribution of the two phenotypes, we analyzed the relative importance, as well as the mean absolute values, of each of the 12 selected variables in the melanistic and non-melanistic models. These comparisons were performed on three different spatial scales (full data set, Central+Southeastern Asia, and Southeast Asia only) to assess whether any consistent pattern distinguishing the phenotypes could be observed. The significance of the observed differences was assessed statistically using a paired t-test, employing a Bonferroni correction to account for multiple comparisons. We then performed in-depth analyses of the two variables displaying differential patterns (see Results), assessing their relationship with vegetational cover and with the estimated habitat suitability for melanistic and non-melanistic leopards.

## Results

We obtained 624 records, comprising 552 non-melanistic individuals, 67 confirmed melanistic individuals, and five unconfirmed melanistic individuals (Fig 1; S1 Table). Our database provided a broad coverage of the known leopard distribution, as well as an update of the species’ current range (S4 and S5 Figs), by filling in geographic gaps in which leopards had not been officially recorded, but were expected to occur [16; 55]. Melanism presented a global frequency of 10.75% across the species’ range. This estimate was mostly driven by the camera-trap records (N = 513; 58 melanistic animals), while that derived from museum specimens (N = 101; 4 melanistic) was lower (see S1 Table). Given the larger sample size, broad geographic coverage and more recent age of the camera-trap data, we consider that its stronger impact on the overall estimate is a positive feature, leading to a representative assessment of melanism frequency across leopard populations.

The presence and frequency of melanism varied considerably among different biomes (Table 1). The confirmed presence of melanistic leopards was recorded only in the following regions (Fig 1): Africa, Central India, Nepal and Bhutan, Sri Lanka, Southeast Asia and Java. All of these regions contained new records for areas in which melanism had been previously described, as well as representation of additional areas. Melanism was absent in the Russian Far East, Central China and Middle East (including the Arabian Peninsula). Additionally, we obtained three unconfirmed melanism records from Africa and two from Iran. These five points were removed from the melanism model, conservatively assuming the absence of melanism in these areas. It is noteworthy that four of these five records were located in areas in which the niche model (see below) indicated low suitability for melanism, whereas the fifth record (located on Mount Kenya) did match an area with high suitability for melanism.

**Table 1:** Chi-square test of association between landscape variables (biomes) and phenotypes (non-melanistic/melanistic). An adjusted residual >2 or <-2 indicates statistical significance for alpha = 0.05.

Biome	Statistics	Leopard Groups		Total
		Non-melanistic	Melanistic	
**Desert and Xeric Shrublands**	Count	69	0	69
	Expected	61.5	7.5	69.0
	% within landscape	100.0%	0.0%	100.0%
	% within groups	12.5%	0.0%	11.1%
	% of Total	11.1%	0.0%	11.1%
	Adjusted Residual	3.1	-3.1	
**Mediterranean Forests. Woodlands. and Scrub**	Count	13	0	13
	Expected	11.6	1.4	79
	% within landscape	100.0%	0.0%	100.0%
	% within groups	2.4%	0.0%	2.1%
	% of Total	2.1%	0.0%	2.1%
	Adjusted Residual	1.3	-1.3	
**Tundra**	Count	2	0	2
	Expected	1.8	0.2	2.0
	% within landscape	100.0%	0.0%	100.0%
	% within groups	0.4%	0.0%	0.3%
	% of Total	0.3%	0.0%	0.3%
	Adjusted Residual	0.5	-0.5	
**Montane Grasslands and Shrublands**	Count	47	3	50
	Expected	44.6	5.4	50.0
	% within landscape	94.0%	6.0%	100.0%
	% within groups	8.5%	4.5%	8.1%
	% of Total	7.6%	0.5%	8.1%
	Adjusted Residual	1.1	-1.1	
**Temperate Broadleaf and Mixed Forests**	Count	108	3	111
	Expected	99.0	12.0	111.0
	% within landscape	97.3%	2.7%	1
	% within groups	19.6%	4.5%	17.9%
	% of Total	17.4%	0.5%	17.9%
	Adjusted Residual	3	-3	
**Temperate Coniferous Forest**	Count	5	0	5
	Expected	4.5	0.5	5.0
	% within landscape	100.0%	0.0%	100.0%
	% within groups	0.9%	0.0%	0.8%
	% of Total	0.8%	0.0%	0.8%
	Adjusted Residual	0.8	-0.8	
**Temperate Grasslands. Savannas and Shrublands**	Count	2	0	2
	Expected	1.8	0.2	2.0
	% within landscape	100.0%	0.0%	100.0%
	% within groups	0.4%	0.0%	0.3%
	% of Total	0.3%	0.0%	0.3%
	Adjusted Residual	0.5	-0.5	
**Tropical and Subtropical Coniferous Forests**	Count	5	0	5
	Expected	4.5	0.5	5.0
	% within landscape	100.0%	0.0%	100.0%
	% within groups	0.9%	0.0%	0.8%
	% of Total	0.8%	0.0%	0.8%
	Adjusted Residual	0.8	-0.8	
**Tropical and Subtropical Dry Broadleaf Forests**	Count	38	2	40
	Expected	35.7	4.3	40.0
	% within landscape	95.0%	5.0%	100.0%
	% within groups	6.9%	3.0%	6.5%
	% of Total	6.1%	0.3%	6.5%
	Adjusted Residual	1.2	-1.2	
**Tropical and Subtropical Grasslands. Savannas and Shrublands**	Count	126	0	126
	Expected	112.4	13.6	126.0
	% within landscape	100.0%	0.0%	100.0%
	% within groups	22.8%	0.0%	20.4%
	% of Total	20.4%	0.0%	20.4%
	Adjusted Residual	4.4	-4.4	
**Tropical and Subtropical Moist Broadleaf Forest**	Count	137	59	196
	Expected	174.8	21.2	196.0
	% within landscape	69.9%	30.1	100.0
	% within groups	24.8%	88.1%	31.7%
	% of Total	22.1%	9.5%	31.7%
	Adjusted Residual	-10.5	10.5	
	Count	552	67	196
	% of Total	89.2%	10.8%	100.0%

*Chi-square = 112,608, likelihood ratio = 118,450, linear-by-linear association = 43,897.

Although leopards were found in 11 different biomes, melanism was only observed in four of them (Table 1), and was most common in tropical and subtropical moist forests (59 of the 67 melanistic records, or 88%), especially in the Indian Ghats (India, n = 8), Javan forests (Indonesia, n = 7), Kayah-Karen/Tenasserim forests (Southeast Asia, n = 16) and Peninsular Malaysian rain forests (Southeast Asia, n = 19) (S5 Fig). In four different biomes, the frequencies of the two phenotypes were significantly different from the null expectation, based on the overall frequency across the range (see Table 1). This was especially the case for tropical and subtropical moist broadleaf forests, where 30% of the animals were black, *i*.*e*. almost three times the expected frequency. In contrast, the frequency of melanism was significantly lower than expected in deserts and xeric scrublands, temperate broadleaf and mixed forests, as well as tropical and subtropical grasslands, savannahs and scrublands (see Table 1).

To further investigate the spatial distribution of the two phenotypes, we performed CSR and niche modeling analyses using a thoroughly filtered database. The filtering step removed 34 records (32 spotted and two melanistic) that consisted of older samples, duplicated occurrences, and points with high rates of anthropogenic landscape modification over the last 13 years (see Methods), yielding a final database of 585 location records.

**Fig 2:**
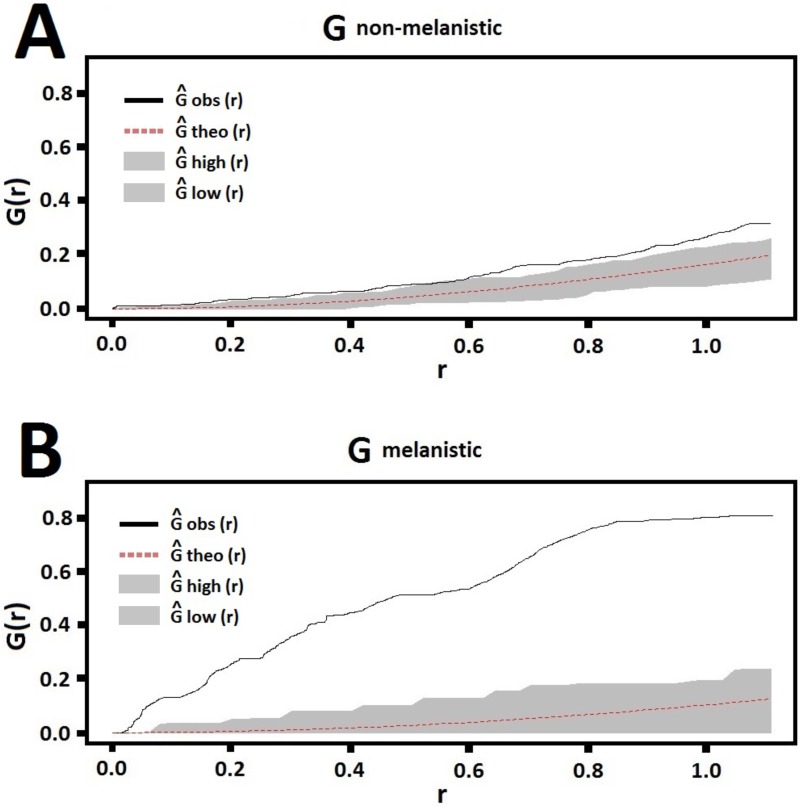
Comparison between distinct phenotypes performed with the CSR test contrasting patterns of random and observed distributions of location records in our database. (A) non-melanistic leopards (p = 0.019) and (B) melanistic leopards (p = 0.004).

The CSR analysis, which yields a G scale showing how the observed distribution deviates from a null model, supported the conclusion that melanism was not evenly distributed across the leopard range. The results obtained for the two sample sets (melanistic and non-melanistic) were strikingly different (Fig 2): while the non-melanistic records presented a spatial distribution that approached randomness within our sampled polygon, the melanistic records strongly deviated from this expected pattern.

**Fig 3:**
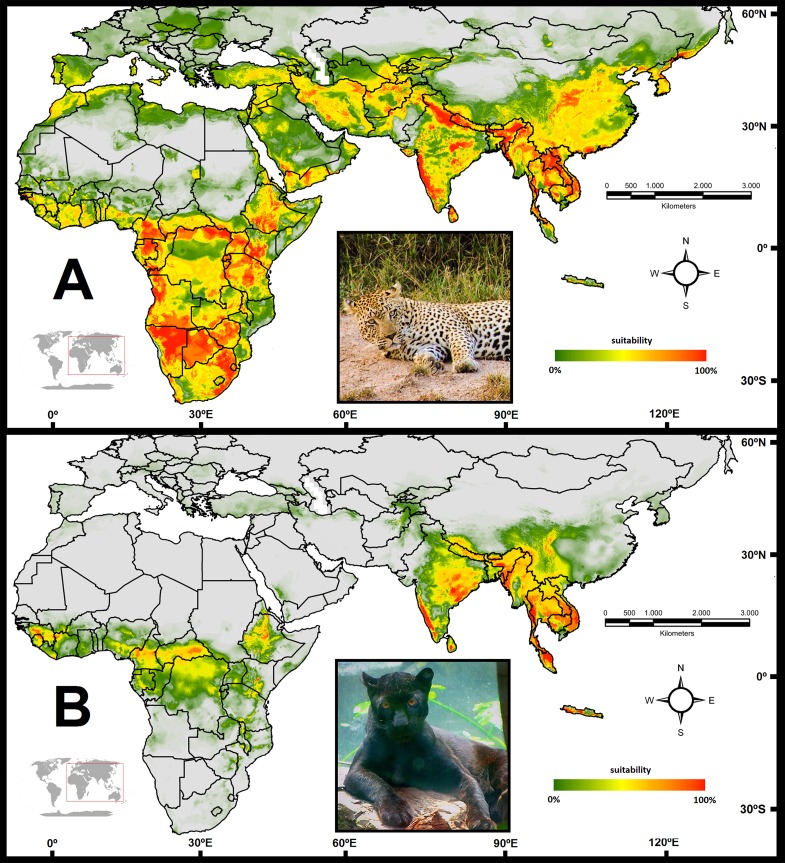
Potential distribution maps of the two coloration phenotypes analyzed in this study. (A) Distribution of non-melanistic leopards and (B) Distribution of melanistic leopards. Photos: Eduardo Eizirik and Lucas G. da Silva.

Niche models generated for both phenotypes (Fig 3) were considered satisfactory (AUC ≥ 0.9): control model of non-melanistic individuals (n = 520): mean AUC = 0.948 (std error 0.004); melanism model (n = 65): mean AUC = 0.982 (std error 0.003). This assessment allowed a comparison between the overall range of the ancestral (non-melanistic) phenotype and that of melanistic animals, showing regional enrichment for this variant in some areas, as well as its absence in many others. In the control (non-melanistic) model, the sampled points presented a mean suitability of 0.594 (standard deviation = 0.167). In contrast, in the melanism model, the sampled points presented a mean suitability of 0.192 (standard deviation = 0.280), as shown in S7 Fig.

The niche models allowed us to compare statistically the habitat suitability between the two coloration phenotypes across the leopard range, revealing a significant difference between them (p<0.01). Additionally, the niche equivalency estimates showed that non-melanistic and melanistic models were significantly different based on Shoener's index (*D* = 0.45, p<0.0001) and Hellinger’s distance (*I* = 0.76, p<0.0001), whereas the relative rank was not significant (*RR* = 0.78, p>0.05).

We then analyzed the environmental variables that were most influential on the two models, and observed that predictors related to precipitation and moisture tended to have a large effect, although their relative importance varied across the different geographic scales (S6 Fig). Interestingly, when we inspected the absolute values of each variable in the two models and across the three scales, we observed a consistent difference between the phenotypes in two predictors (‘Annual precipitation’ and ‘Mean moisture index of driest quarter’). For both variables, we observed consistently higher values for melanistic leopards than for non-melanistic leopards, across the three scales (see S6 Fig). These differences were highly significant (p≤0.002) for both variables on the two largest scales, but non-significant (p>0.05) for the more restricted scale (Southeast Asia), possibly due to its smaller sample size and proportionally larger variance.

We subsequently performed an in-depth investigation of these two variables, assessing their correlation with each other and with an index of vegetation cover (NDVI), and then analyzing the relationship between their values (and the NDVI) and the habitat suitability for melanistic and non-melanistic leopards, across the three spatial scales (S8 Fig). We observed that the two bioclimatic variables are positively correlated with each other and with the NDVI on the two largest spatial scales, but no clear relationship was discerned on the smallest extent. When we plotted the two variables and NDVI against the estimated habitat suitability for melanistic (M) and non-melanistic (NM) leopards, an interesting pattern emerged (see S8 Fig). No obvious difference between the two models was apparent on the largest and smallest scales, but the intermediate scale (comprising central and southeastern Asia) did reveal an interesting pattern. There was a positive correlation between habitat suitability and two variables (NDVI and ‘Annual precipitation’) for the two phenotypes, but the slope for melanistic leopards was steeper in both cases (NDVI: R = 0.318 for NM and R = 0.359 for M; ‘Annual Precipitation’: R = 0.186 for NM and R = 0.300 for M). The difference in slope was even more apparent for the ‘Mean moisture index of driest quarter’, with no correlation observed for non-melanistic animals (R = 0.035) and a visible correlation detected for the melanistic model (R = 0.296).

## Discussion

The database assembled in this study allowed the construction of the first map of the occurrence of melanism in leopards (Figs 1 and 3), which could be directly compared to an updated distribution map for the species as a whole (S2 Fig). We employed these maps and the associated database to investigate the spatial pattern of occurrence of black panthers, and to assess potential causes for the observed distribution.

Our data revealed that melanism occurs non-randomly across the leopard's range, as demonstrated by the CSR test and the significantly different niche models for the two phenotypes. Moist forests (especially in Southeast Asia) presented very high frequencies of melanistic leopards (*e*.*g*. 39 of the 71 individuals [55%] sampled in Southeast Asia), and more than 80% of the black animals in our global database, a five-fold increase relative to the expected number based on the overall average. Furthermore, we found no confirmed melanistic leopards in the Middle East, Arabian Peninsula, Central China and Russian Far East (Fig 1), nor any citation in the literature as to the presence of melanism in these regions, indicating that this variant is absent in the leopards occurring in these areas. Furthermore, the frequency of melanism was significantly below the global average in some biomes that consist of open habitats or temperate forests (Table 1). Therefore, there is a clear pattern in which melanism tends to increase in tropical/subtropical moist forests, and to decrease in open/dry or temperate habitats. Such a result supports the classical hypotheses (*e*.*g*. Gloger’s rule) postulating an adaptive role for melanism, which would be favored in tropical and humid environments [1; 6; 12–13]. Conversely, these results indicate that melanism is selected against in open/dry and temperate habitats. Such hypotheses had so far not been directly tested for leopards, and the present demonstration that this pattern is indeed significant opens up opportunities to further investigate the underlying processes.

To assess the robustness of the observed pattern and its relationship to various environmental predictors, we performed a suite of analyses based on ecological niche modeling. The models generated in our study were found to be robust, as they showed realistic estimates of habitat suitability for leopard occurrence on a broad geographic scale. We tested the predictive power of the melanism model by removing parts of the sample set and observing the robustness of the estimated spatial patterns. We particularly focused on regions with low sampling and presence of melanism, whose reliability might be lower. When we removed all the samples from Java and ran the model again, the output map maintained the high suitability for black leopards on that island, as observed in the original model. The same result was obtained when we removed the only confirmed melanistic record from Africa, with the model still indicating a high suitability for melanistic leopards at the sampled location. Such observations lend confidence to the reliability of the models generated in this study, and their potential to serve as a basis for in-depth spatial analyses.

We then compared in detail the models generated for non-melanistic and melanistic leopards, and observed that they displayed marked spatial differences (Fig 3). Furthermore, the models were significantly different when compared with both the t-test and the niche equivalency analysis. When we analyzed the influence of environmental predictors on habitat suitability for melanistic and non-melanistic leopards, we identified two moisture-related variables (‘Annual precipitation’ and ‘Mean moisture index of driest quarter’) that were significantly different between the two models (see S6 Fig). Interestingly, we did not observe any differential pattern for variables related to temperature, suggesting that this factor is not as influential as moisture on the occurrence of leopard melanism. The two variables exhibiting a differential pattern were correlated with an index of vegetational cover (NDVI), suggesting that they might exert their effect on suitability indirectly, via an influence on the habitat type itself (see S8 Fig). This would support the interpretation that the mechanism underlying the selective advantage of melanism in tropical forests is camouflage/crypsis, and that these moisture-related variables are only tracking differences in the presence of closed-canopy forests.

However, a non-exclusive hypothesis is that moisture itself may exert a selective influence on leopard melanism. We tested this idea by comparing habitat suitability for each phenotype with these three variables (two moisture-related predictors and NDVI) on three different spatial scales (see S8 Fig). The intermediate scale (encompassing central and southeastern Asia) revealed a stronger correlation of all three variables with habitat suitability for melanistic leopards, relative to non-melanistic animals (using the same set of location records for both phenotypes). This pattern was strongest for one of the moisture-related predictors (‘Mean moisture index of driest quarter’), which showed no relationship with suitability for non-melanistic leopards, but substantial correlation with suitability for melanistic animals (see Results and S8 Fig). This result, compared with the relationship observed with NDVI at the same points, suggests that moisture is related to melanism to a greater extent than can be attributed solely to its influence on habitat type. Given the available data for birds (*e*.*g*. [56]) demonstrating a relationship between darker coloration and resistance to feather-degrading bacteria in humid habitats, one can hypothesize a similar selective pressure for mammals (*e*.*g*. [57]. Testing this bacterial-related selection directly, along with its potential interaction with a camouflage-related pressure, is an interesting avenue for future research focusing on leopards and other mammals displaying similar patterns (*e*.*g*. [58]).

To assess whether our results might also be influenced by non-selective factors, we investigated the possible effect of demographic processes such as population structure and drift-induced differentiation. The existence of phylogeographic structure in leopards [30] raises the possibility that restricted historical gene flow among some portions of the range might influence the present distribution of variable phenotypes. Therefore, we inspected the spatial patterns observed within subspecies (as defined by [30]) that harbor melanism and for which we had the largest sample sizes, thus focusing on India and Southeast Asia. The same patterns of habitat association observed for the full data set were affirmed for these subspecies-specific data sets, strongly supporting a selective rather than a demographic explanation for the presence and frequency of melanism.

We explored this comparison in more depth using the Southeast Asia data set (S5 Fig — panel E). Our data support the observations reported by [17] and [18], showing that leopard melanism is almost fixed in areas south of the Isthmus of Kra (Thailand/Malaysia). We obtained only two records of non-melanistic animals south of the Isthmus (the same was reported by [59]), while in more northerly areas both phenotypes appear at similar frequencies. This intriguing regional pattern may have been influenced by some degree of demographic isolation across the Isthmus, which is consistent with the hypothesis that in the past (during the Last Glacial Maximum, 20,000–25,000 years ago [60]) it operated as an effective barrier restricting gene flow for several organisms (*e*.*g*. [61]). A phylogeographic break in this area has been reported for several felid species [62–63], supporting the inference of historical partitions in this region consistently affecting this group. Interestingly, the data available for leopards [63] do not support such a partition in this species, indicating that it was either not affected by such gene-flow restrictions, or less affected than other felids.

If such a demographic explanation does not seem to strongly account for the observed pattern, neither does selection based on habitat type, since both sides of the Isthmus harbor similar landscapes (the whole region falls within the ‘Tropical and Subtropical Moist Broadleaf Forests’ biome [see S5 Fig — panel E]). This led us to hypothesize that variation in moisture (beyond its influence on vegetation) might underlie the difference in the frequency of leopard melanism south *vs*. north of the Isthmus of Kra. To test this, we generated 600 random points in the Malay Peninsula, from which we extracted the value for the ‘Mean moisture index of driest quarter’. The points were divided exactly between the two halves (50% north and 50% south of the Isthmus), covered approximately the same area on either side, and were only placed in regions classified as ‘Tropical and Subtropical Moist Broadleaf Forest’. Remarkably, the difference in moisture between the two sides of the Isthmus was highly significant (t-test; p<0.001), with the southern side (where melanism is almost fixed) bearing much higher values. This result supports the inference that a selective pressure directly related to humidity (beyond its impact on vegetation) influences the frequency of melanism in leopards.

Given these findings, we consider that the most probable historical scenario for melanism in leopards is the emergence of the causative allele at a particular location (likely southeast Asia) and its dispersal throughout much of the species’ distribution, undergoing natural selection driven by habitat type and humidity in different landscapes, as well as genetic drift in some situations (*e*.*g*. founding of new populations during range expansion events, or episodic disruption of gene flow). Since melanism in leopards is a recessive trait [13], it is plausible that its causative allele can disperse long distances over evolutionary time even across habitats where it could be selected against (*e*.*g*. deserts and grasslands). This is because the allele can remain "hidden" in heterozygosity for many generations when it is at low frequency, while at the same time it could be lost in some areas due to genetic drift. Another possibility is that melanism arose in leopards more than once, *e*.*g*. hypothesizing a distinct mutation emerging in Africa, since the known *ASIP* mutation was reported only based on Asian samples [13]. This hypothesis can be directly tested with molecular approaches targeting the implicated region of the *ASIP* gene in melanistic animals sampled in Africa. Still, even assuming multiple origins in different regions, the same process of long-range dispersal across unfavorable habitats would have to be postulated.

In summary, after over 100 years of anecdotal appearances in the scientific and popular literature, but no direct assessment with rigorous approaches, this study has provided a characterization of the spatial distribution of melanism in leopards. We demonstrate that this distribution is non-random across the species’ range, with the observed spatial patterns significantly supporting an association with moist forests and a decrease in frequency in open/dry habitats. While these results support classical adaptive hypotheses, implying that melanism in leopards is influenced by natural selection related to habitat type and moisture, several questions remain unanswered, such as the exact selective mechanisms in different areas. The results and analyses reported here may serve as a useful basis for studies addressing these questions, shedding further light on the ecological and evolutionary dynamics of this remarkable coloration variant.

## Supporting information

S1 Table *Panthera pardus* location records used in the present study. (PDF)

S2 Table Environmental predictors used in the initial analysis and selected with Pearson's test (in red). (PDF)

S1 Fig Base map for the CSR test. (PDF)

S2 Fig Response curves observed in the Maxent analysis for each environmental predictor used to construct the non-melanistic (control) model. (PDF)

S3 Fig Response curves observed in the Maxent analysis for each environmental predictor used to construct the melanistic model. (PDF)

S4 Fig New distributional map for *Panthera pardus*. Location records comprising our full database are indicated, and overlaid on the present IUCN range map along with additional areas of occurrence documented in this study. Subspecies partitions proposed by Uphyrkina et al. (2001) are also indicated, including summaries of the number records of each coloration phenotype per subspecies.
(PDF)

S5 Fig Detailed maps showing the geographic distribution of records comprising our database of melanistic and non-melanistic leopards, overlaid on the terrestrial biomes (based on Olson et al. 2001). Each major geographic region representing a leopard subspecies is shown in a separate panel.
(PDF)

S6 Fig Detailed assessment of the 12 bioclimatic predictors selected for inclusion in the Maxent modeling of the two coloration phenotypes, after removing the variables showing the most correlation relative to all others (see Methods). A) Relative importance of each variable for modeling habitat suitability, depicted separately for three geographic scales: (i) full data set (‘Non-melanistic’ and ‘melanistic’ in the legend); (ii) Central+Southeastern Asia [*P*. *p*. *fusca*, *P*. *p*. *melas*, *P*. *p*. *delacouri*, *P*. *p*. *japonensis* and *P*. *p*. *kotiya* in S5 Fig] (‘Non-melanistic Asia’ and ‘melanistic Asia’ in the legend); (iii) (ii) Southeast Asia [*P*. *p*. *delacouri* in S5 Fig] (‘Non-melanistic SE Asia’ and ‘melanistic SE Asia’ in the legend). B) Mean absolute values of each of the 12 selected variables for the same six groups (phenotypes *vs*. geographic scales) depicted on panel A. Arrows indicate two variables (‘Annual precipitation’ and ‘Mean moisture index of driest quarter’) that exhibited a consistent pattern of differential effects on the two phenotype-based models, across the three assessed geographic scales.
(PDF)

S7 Fig Graphs depicting the results of the suitability test comparing the melanistic and non-melanistic models across all the location records in our database (p<0.001). Mean suitability in the non-melanistic model = 0.594 (standard deviation = 0.167); mean suitability in the melanistic model = 0.192 (standard deviation = 0.280).
(PDF)

S8 Fig In-depth analysis of environmental variables and their relationship with melanism in leopards. The top three graphs (panel A) depict the relationships between the two variables identified as having differential effects on the two phenotypes (see S6 Fig), as well as their relationship with a measure of cover (vegetation index NDVI). For each graph, the relationship between the assessed variables is shown for three different geographic scales (as defined in S6 Fig). Panels B-D depict the relationship between each of these three explanatory variables and the habitat suitability estimated for melanistic and non-melanistic leopards, shown separately for the same three geographic scales.
(PDF)
