# Supplementary material for: Mapping black panthers: Macroecological modeling of melanism in leopards (Panthera pardus)
Source: PLoS One. 2017 Apr 5;12(4):e0170378. doi: 10.1371/journal.pone.0170378 (PMC5381760; doi:10.1371/journal.pone.0170378)
Supplement: S1 Table — (PDF) [file pone.0170378.s001.pdf]

S1 Table - Panthera pardus location records used in the present study.

Id	Subspecies	Location	Deg. - WGS84		Biome	Ecorregion	Sample	Analysis	Year	Source
			Latitude	Longitude						
Doubtful Mel. 01	*saxicolor*	Tandoureh National Park, Iran	37.3762	58.5006	Temperate Coniferous Forest	Caucasus-Anatolian-Hyrcanian temperate forests	Report	All	no data	Asian Leopard Group
Doubtful Mel. 02	*pardus*	Chyulu Hills National Park, Kenya	-2.8161	38.1473	Tropical and Subtropical Grasslands, Savannas and Shrublands	East African Acacia Savannas	Report	All	no data	Lion Guardians
Doubtful Mel. 03	*pardus*	Mount Kenya's, Kenya	-0.3062	37.1866	Tropical and Subtropical Moist Broadleaf Forests	East African montane forests	Report	All	no data	Video Mount Kenya/Sunquist & Sunquist 2002
Doubtful Mel. 04	*pardus*	Lydenburg, South Africa	-24.9221	30.6565	Montane Grasslands and Shrublands	Drakensberg Montane Woodlands and Grasslands	Report	All	no data	Sharon Hammond
Doubtful Mel. 05	*saxicolor*	Garmnsar, Semnan Province, Iran	34.9385	52.5348	Deserts and Xeric Shrublands	Central Persian desert basins	Report	All	no data	Asian Leopard Group
Melanistic 01	*fusca*	Manas National Park, Bhutan	26.8011	91.0330	Tropical and Subtropical Moist Broadleaf Forests	Brahmaputra Valley semi-evergreen forests	Photograph	All	no data	Smithsonian Institution
Melanistic 02	*fusca*	Tsamang, Monggar, Bhutan	27.5048	91.1057	Temperate Broadleaf and Mixed Forests	Eastern Himalayan broadleaf forests	Photograph	All	no data	Smithsonian Institution
Melanistic 03	*fusca*	Southern Lung, China	28.2364	92.7316	Montane Grasslands and Shrublands	Eastern Himalayan alpine shrub and meadows	Photograph	All	no data	Smithsonian Institution
Melanistic 04	*pardus*	Addis Ababa, Abissynya, Ethiopia	9.0445	38.6967	Montane Grasslands and Shrublands	Ethiopian montane grasslands and woodlands	Photograph	Filtered	1909	National Museum of Natural History USA
Melanistic 05	*fusca*	Achanakmar Tiger Reserve, India	22.4528	81.5555	Tropical and Subtropical Moist Broadleaf Forests	Eastern highlands moist deciduous forests	Report	All	no data	Milind Pariwakam/Biswajit Mohanty
Melanistic 06	*fusca*	Achanakmar Tiger Reserve, India	22.5980	81.8327	Tropical and Subtropical Moist Broadleaf Forests	Eastern highlands moist deciduous forests	Photograph	All	no data	Milind Pariwakam/Biswajit Mohanty
Melanistic 07	*fusca*	Achanakmar Tiger Reserve, India	22.3421	81.8245	Tropical and Subtropical Moist Broadleaf Forests	Eastern highlands moist deciduous forests	Report	All	no data	Milind Pariwakam/Biswajit Mohanty
Melanistic 08	*fusca*	Bhadra Tiger Reserve, India	13.4910	75.6496	Tropical and Subtropical Moist Broadleaf Forests	North Western Ghats moist deciduous forests	Photograph	All	2012	Conservation India
Melanistic 09	*fusca*	Bhadra Tiger Reserve, India	13.6189	75.6286	Tropical and Subtropical Moist Broadleaf Forests	North Western Ghats moist deciduous forests	Photograph	All	2012	Conservation India
Melanistic 10	*fusca*	Chhattisgarh, India	23.9813	82.0120	Tropical and Subtropical Dry Broadleaf Forests	Narmada Valley dry deciduous forests	Report	All	no data	Milind Pariwakam/Biswajit Mohanty
Melanistic 11	*fusca*	Dandeli-Anshi Tiger Reserve, india	13.1213	75.0433	Tropical and Subtropical Moist Broadleaf Forests	Malabar Coast moist forests	Photograph	All	2013	Wildlife Conservation Society
Melanistic 12	*fusca*	Dibrugarh, India	27.4848	95.0622	Tropical and Subtropical Moist Broadleaf Forests	Brahmaputra Valley semi-evergreen forests	Photograph	All	no data	Kashmira Kakati
Melanistic 13	*fusca*	Kaziranga National Park, India	26.6911	93.4945	Tropical and Subtropical Moist Broadleaf Forests	Brahmaputra Valley semi-evergreen forests	Photograph	All	no data	World Wild Fund
Melanistic 14	*fusca*	Maijan Bungalow Brahmaputra Ghat, India	27.5008	94.9323	Tropical and Subtropical Moist Broadleaf Forests	Brahmaputra Valley semi-evergreen forests	Photograph	All	no data	Kashmira Kakati
Melanistic 15	*fusca*	Mudumalai National Park, Central India, India	11.5087	76.5651	Tropical and Subtropical Moist Broadleaf Forests	South Western Ghats moist deciduous forests	Photograph	All	2013	Phillip Ross
Melanistic 16	*fusca*	Orissa, India	20.3555	80.9707	Tropical and Subtropical Moist Broadleaf Forests	Eastern highlands moist deciduous forests	Report	All	no data	Milind Pariwakam/Biswajit Mohanty
Melanistic 17	*fusca*	Pakke Tiger Reserve in Arunachal Pradesh, India	28.5669	95.9691	Temperate Broadleaf and Mixed Forests	Eastern Himalayan broadleaf forests	Photograph	All	2013	Milind Pariwakam/Biswajit Mohanty
Melanistic 18	*fusca*	Pakke Tiger Reserve in Arunachal Pradesh, India	28.6604	95.6358	Temperate Broadleaf and Mixed Forests	Eastern Himalayan broadleaf forests	Photograph	All	no data	Milind Pariwakam/Biswajit Mohanty
Melanistic 19	*fusca*	Periyar Wildlife Sanctuary, India	9.2756	76.9211	Tropical and Subtropical Moist Broadleaf Forests	South Western Ghats moist deciduous forests	Report	All	no data	Milind Pariwakam/Biswajit Mohanty
Melanistic 20	*melas*	Baluran National Park, Java, Indonesia	-7.8531	114.4085	Tropical and Subtropical Moist Broadleaf Forests	Eastern Java-Bali rain forests	Photograph	All	2012	Copenhague Zoo
Melanistic 21	*melas*	Gunung Gede Pangrango National Park, West Java, Indonesia	-6.8038	106.9310	Tropical and Subtropical Moist Broadleaf Forests	Western Java montane rain forests	Photograph	All	no data	Anton Ario
Melanistic 22	*melas*	Gunung Salak National Park, Java, Indonesia	-7.1238	107.3197	Tropical and Subtropical Moist Broadleaf Forests	Western Java montane rain forests	Photograph	All	no data	CIFOR
Melanistic 23	*melas*	Halimun-Salak, Java, Indonesia	-6.7671	106.5601	Tropical and Subtropical Moist Broadleaf Forests	Western Java montane rain forests	Photograph	All	2004	Anhar Harahap
Melanistic 24	*melas*	Halimun-Salak, Java, Indonesia	-6.7832	106.6828	Tropical and Subtropical Moist Broadleaf Forests	Western Java montane rain forests	Photograph	All	2005	Anhar Harahap
Melanistic 25	*melas*	Ujung Kulon National Park, Java, Indonesia	-6.7524	105.3290	Tropical and Subtropical Moist Broadleaf Forests	Western Java rain forests	Photograph	All	no data	World Wild Fund
Melanistic 26	*melas*	Western Java, Indonesia	-6.6280	105.9386	Tropical and Subtropical Moist Broadleaf Forests	Western Java rain forests	Photograph	Filtered	1907	National Museum of Natural History USA
Melanistic 27	*delacouri*	Kenyir Wildlife Corridor, Malaysia	5.2801	102.6407	Tropical and Subtropical Moist Broadleaf Forests	Peninsular Malaysian rain forests	Photograph	All	2012	Reuben Clements
Melanistic 28	*delacouri*	Kenyir Wildlife Corridor, Malaysia	5.1205	102.9918	Tropical and Subtropical Moist Broadleaf Forests	Peninsular Malaysian rain forests	Photograph	All	no data	Kenyir Wildlife Corridor Leopard Project
Melanistic 29	*delacouri*	Kenyir Wildlife Corridor, Malaysia	5.2390	102.7215	Tropical and Subtropical Moist Broadleaf Forests	Peninsular Malaysian rain forests	Photograph	All	2011	Kae Kawanishi
Melanistic 30	*delacouri*	Malay Peninsula, Malaysia	3.3657	102.1930	Tropical and Subtropical Moist Broadleaf Forests	Peninsular Malaysian rain forests	Photograph	All	2009	Kae Kawanishi
Melanistic 31	*delacouri*	Malay Peninsula, Malaysia	4.4303	103.1427	Tropical and Subtropical Moist Broadleaf Forests	Peninsular Malaysian rain forests	Photograph	All	2009	Kae Kawanishi
Melanistic 32	*delacouri*	Malay Peninsula, Malaysia	4.4536	102.5160	Tropical and Subtropical Moist Broadleaf Forests	Peninsular Malaysian rain forests	Photograph	All	2009	Kae Kawanishi
Melanistic 33	*delacouri*	Malay Peninsula, Malaysia	4.2904	102.3561	Tropical and Subtropical Moist Broadleaf Forests	Peninsular Malaysian rain forests	Photograph	All	2009	Kae Kawanishi
Melanistic 34	*delacouri*	Malay Peninsula, Malaysia	4.0748	102.8376	Tropical and Subtropical Moist Broadleaf Forests	Peninsular Malaysian rain forests	Photograph	All	2009	Kae Kawanishi
Melanistic 35	*delacouri*	Malay Peninsula, Malaysia	4.1564	102.5324	Tropical and Subtropical Moist Broadleaf Forests	Peninsular Malaysian rain forests	Photograph	All	2009	Kae Kawanishi
Melanistic 36	*delacouri*	Malay Peninsula, Malaysia	4.2963	102.7504	Tropical and Subtropical Moist Broadleaf Forests	Peninsular Malaysian rain forests	Photograph	All	2009	Kae Kawanishi
Melanistic 37	*delacouri*	Malay Peninsula, Malaysia	5.4888	102.1140	Tropical and Subtropical Moist Broadleaf Forests	Peninsular Malaysian rain forests	Photograph	All	2009	Kae Kawanishi
Melanistic 38	*delacouri*	Malay Peninsula, Malaysia	5.7067	101.9804	Tropical and Subtropical Moist Broadleaf Forests	Peninsular Malaysian rain forests	Photograph	All	2009	Kae Kawanishi
Melanistic 39	*delacouri*	Malay Peninsula, Malaysia	5.4091	101.6545	Tropical and Subtropical Moist Broadleaf Forests	Peninsular Malaysian rain forests	Photograph	All	2009	Kae Kawanishi
Melanistic 40	*delacouri*	Malay Peninsula, Malaysia	5.6240	101.7890	Tropical and Subtropical Moist Broadleaf Forests	Peninsular Malaysian rain forests	Photograph	All	2009	Kae Kawanishi
Melanistic 41	*delacouri*	Malay Peninsula, Malaysia	5.7618	101.5769	Tropical and Subtropical Moist Broadleaf Forests	Peninsular Malaysian rain forests	Photograph	All	2009	Kae Kawanishi
Melanistic 42	*delacouri*	Malay Peninsula, Thailand	12.8965	99.3874	Tropical and Subtropical Moist Broadleaf Forests	Kayah-Karen/Tenasserim moist forests	Photograph	All	2009	Kae Kawanishi
Melanistic 43	*delacouri*	Malay Peninsula, Thailand	13.1375	99.2334	Tropical and Subtropical Moist Broadleaf Forests	Kayah-Karen/Tenasserim moist forests	Photograph	All	2009	Kae Kawanishi
Melanistic 44	*delacouri*	Malay Peninsula, Malaysia	6.1715	101.0211	Tropical and Subtropical Moist Broadleaf Forests	Peninsular Malaysian rain forests	Photograph	All	no data	American Museum of Natural History USA
Melanistic 45	*delacouri*	Taman Negara Park, Malaysia	5.2790	102.4918	Tropical and Subtropical Moist Broadleaf Forests	Peninsular Malaysian rain forests	Photograph	All	2010	Kae Kawanishi
Melanistic 46	*fusca*	Kangchenjunga Conservation Area, Nepal	27.7408	87.9714	Montane Grasslands and Shrublands	Eastern Himalayan alpine meadows	Photograph	All	2013	Thapa et al 2013
Melanistic 47	*kotiya*	Deniyaya, Sri Lanka	6.7561	80.6977	Tropical and Subtropical Moist Broadleaf Forests	Sri Lankan moist forest	Photograph	All	no data	Andrew Kittle
Melanistic 48	*delacouri*	Ban Krang, Thailand	16.7477	100.2027	Tropical and Subtropical Moist Broadleaf Forests	Chao Phraya freshwater swamp forests	Photograph	All	no data	American Museum of Natural History USA
Melanistic 49	*delacouri*	Chiang Mai, Thailand	18.7627	98.8565	Tropical and Subtropical Moist Broadleaf Forests	Kayah-Karen/Tenasserim moist forests	Photograph	All	2013	Bruce Kekule
Melanistic 50	*delacouri*	Huai Kha Khaeng Wildlife Sanctuary, Thailand	15.6539	99.5247	Tropical and Subtropical Dry Broadleaf Forests	Indochina dry forests	Photograph	All	2009	Bruce Kekule
Melanistic 51	*delacouri*	Huai Kha Khaeng Wildlife Sanctuary, Thailand	15.1661	99.2784	Tropical and Subtropical Moist Broadleaf Forests	Kayah-Karen/Tenasserim moist forests	Photograph	All	2013	Bruce Kekule
Melanistic 52	*delacouri*	Huai Kha Khaeng Wildlife Sanctuary, Thailand	15.6986	98.7621	Tropical and Subtropical Moist Broadleaf Forests	Kayah-Karen/Tenasserim moist forests	Photograph	All	no data	Kae Kawanishi
Melanistic 53	*delacouri*	Huai Kha Khaeng Wildlife Sanctuary, Thailand	15.5612	98.9229	Tropical and Subtropical Moist Broadleaf Forests	Kayah-Karen/Tenasserim moist forests	Photograph	All	2013	Bruce Kekule
Melanistic 54	*delacouri*	Huai Kha Khaeng Wildlife Sanctuary, Thailand	15.0417	98.3983	Tropical and Subtropical Moist Broadleaf Forests	Chao Phraya lowland moist deciduous forests	Photograph	All	2012	Wildlife Conservation Society
Melanistic 55	*delacouri*	Huai Kha Khaeng Wildlife Sanctuary, Thailand	14.4518	98.8936	Tropical and Subtropical Moist Broadleaf Forests	Chao Phraya lowland moist deciduous forests	Photograph	All	2012	Wildlife Conservation Society
Melanistic 56	*delacouri*	Huai Kha Khaeng Wildlife Sanctuary, Thailand	15.3042	99.2820	Tropical and Subtropical Moist Broadleaf Forests	Kayah-Karen/Tenasserim moist forests	Photograph	All	2013	Wildlife Conservation Society

Id	Subspecies	Location	Deg. - WGS84		Biome	Ecorregion	Sample	Analysis	Year	Source
			Latitude	Longitude						
Melanistic 57	*delacouri*	Huai Kha Khaeng Wildlife Sanctuary, Thailand	15.1038	99.1074	Tropical and Subtropical Moist Broadleaf Forests	Kayah-Karen/Tenasserim moist forests	Photograph	All	2013	Wildlife Conservation Society
Melanistic 58	*delacouri*	Huai Kha Khaeng Wildlife Sanctuary, Thailand	15.1941	98.9874	Tropical and Subtropical Moist Broadleaf Forests	Kayah-Karen/Tenasserim moist forests	Photograph	All	2013	Wildlife Conservation Society
Melanistic 59	*delacouri*	Kaeng Krachan National Park, Thailand	13.0750	99.5457	Tropical and Subtropical Moist Broadleaf Forests	Kayah-Karen/Tenasserim moist forests	Photograph	All	2009	Bruce Kekule
Melanistic 60	*delacouri*	Kaeng Krachan National Park, Thailand	13.1241	99.4217	Tropical and Subtropical Moist Broadleaf Forests	Kayah-Karen/Tenasserim moist forests	Photograph	All	2009	Bruce Kekule
Melanistic 61	*delacouri*	Kaeng Krachan National Park, Thailand	13.0125	99.2634	Tropical and Subtropical Moist Broadleaf Forests	Kayah-Karen/Tenasserim moist forests	Photograph	All	2009	Bruce Kekule
Melanistic 62	*delacouri*	Khao Sok National Park, Thailand	8.9317	98.5110	Tropical and Subtropical Moist Broadleaf Forests	Kayah-Karen/Tenasserim moist forests	Photograph	All	no data	Wildlife Conservation Society
Melanistic 63	*delacouri*	Kuiburi National Park, Thailand	12.3004	99.5971	Tropical and Subtropical Moist Broadleaf Forests	Kayah-Karen/Tenasserim moist forests	Photograph	All	no data	Kae Kawanishi
Melanistic 64	*delacouri*	Malay Peninsula, Thailand	8.5951	98.4122	Tropical and Subtropical Moist Broadleaf Forests	Kayah-Karen/Tenasserim moist forests	Photograph	All	2009	Kae Kawanishi
Melanistic 65	*delacouri*	Malay Peninsula, Malaysia	3.2732	102.3561	Tropical and Subtropical Moist Broadleaf Forests	Peninsular Malaysian rain forests	Photograph	All	2009	Kae Kawanishi
Melanistic 66	*delacouri*	Ban Krang, Thailand	12.9953	99.3327	Tropical and Subtropical Moist Broadleaf Forests	Kayah-Karen/Tenasserim moist forests	Photograph	All	2013	Bruce Kekule
Melanistic 67	*delacouri*	Kenyir Wildlife Corridor, Malaysia	4.7589	102.8283	Tropical and Subtropical Moist Broadleaf Forests	Peninsular Malaysian rain forests	Photograph	All	2013	International Society of Zoological Sciences
Non-melanistic 01	*fusca*	Afghanistan Central Highlands, Afeghanistan	35.4703	70.7899	Montane Grasslands and Shrublands	Middle Asian montane woodlands and steppe	Photograph	All	2011	Wildlife Conservation Society
Non-melanistic 02	*pardus*	Chitau, Bie, Angola	-12.9522	22.6538	Tropical and Subtropical Grasslands, Savannas and Shrublands	Angolan Miombo woodlands	Photograph	Filtered	1925	American Museum of Natural History USA
Non-melanistic 03	*pardus*	Chitau, Bie, Angola	-12.9522	22.6538	Tropical and Subtropical Grasslands, Savannas and Shrublands	Angolan Miombo woodlands	Photograph	Filtered	1925	American Museum of Natural History USA
Non-melanistic 04	*pardus*	Chitau, Bie, Angola	-12.9522	22.6538	Tropical and Subtropical Grasslands, Savannas and Shrublands	Angolan Miombo woodlands	Photograph	Filtered	1925	American Museum of Natural History USA
Non-melanistic 05	*pardus*	Chitau, Bie, Angola	-12.7876	22.6620	Tropical and Subtropical Grasslands, Savannas and Shrublands	Angolan Miombo woodlands	Photograph	Filtered	1925	American Museum of Natural History USA
Non-melanistic 06	*pardus*	Chitau, Bie, Angola	-12.7876	22.6620	Tropical and Subtropical Grasslands, Savannas and Shrublands	Angolan Miombo woodlands	Photograph	Filtered	1925	American Museum of Natural History USA
Non-melanistic 07	*saxicolor*	Zanguezur State Sanctuary, Armenia	39.0436	46.4373	Temperate Broadleaf and Mixed Forests	Caucasus-Anatolian-Hyrcanian temperate forests	Photograph	All	no data	Thomas Gray
Non-melanistic 08	*fusca*	Manas National Park, Bhutan	26.8923	91.0124	Tropical and Subtropical Moist Broadleaf Forests	Himalayan subtropical broadleaf forests	Photograph	All	no data	Smithsonian Institution
Non-melanistic 09	*fusca*	Manas National Park, Bhutan	26.8480	91.1996	Tropical and Subtropical Moist Broadleaf Forests	Himalayan subtropical broadleaf forests	Photograph	All	no data	Smithsonian Institution
Non-melanistic 10	*fusca*	Manas National Park, Bhutan	26.8270	91.1957	Tropical and Subtropical Moist Broadleaf Forests	Himalayan subtropical broadleaf forests	Photograph	All	no data	Smithsonian Institution
Non-melanistic 11	*pardus*	Central Kalahari Reserve, Botswana	-23.1392	24.1780	Deserts and Xeric Shrublands	Kalahari xeric savanna	Photograph	All	no data	Andrew Stein
Non-melanistic 12	*pardus*	Central Kalahari Reserve, Botswana	-23.1096	24.0939	Deserts and Xeric Shrublands	Kalahari xeric savanna	Photograph	All	no data	Andrew Stein
Non-melanistic 13	*pardus*	Central Kalahari Reserve, Botswana	-22.1384	23.7266	Deserts and Xeric Shrublands	Kalahari xeric savanna	Photograph	All	no data	Andrew Stein
Non-melanistic 14	*pardus*	Central Kalahari Reserve, Botswana	-21.7793	23.2238	Tropical and Subtropical Grasslands, Savannas and Shrublands	Kalahari Acacia-Baikiaea woodlands	Photograph	All	no data	Andrew Stein
Non-melanistic 15	*pardus*	Central Kalahari Reserve, Botswana	-21.5270	24.1481	Tropical and Subtropical Grasslands, Savannas and Shrublands	Kalahari Acacia-Baikiaea woodlands	Photograph	All	no data	Andrew Stein
Non-melanistic 16	*pardus*	Central Kalahari Reserve, Botswana	-21.2204	23.3095	Tropical and Subtropical Grasslands, Savannas and Shrublands	Kalahari Acacia-Baikiaea woodlands	Photograph	All	no data	Andrew Stein
Non-melanistic 17	*pardus*	Chobe National Park, Botswana	-18.8340	24.1883	Tropical and Subtropical Grasslands, Savannas and Shrublands	Zambezian and Mopane woodlands	Photograph	All	no data	Andrew Stein
Non-melanistic 18	*pardus*	Chobe National Park, Botswana	-18.5234	24.4231	Tropical and Subtropical Grasslands, Savannas and Shrublands	Zambezian Baikiaea woodlands	Photograph	All	no data	Andrew Stein
Non-melanistic 19	*pardus*	Chobe National Park, Botswana	-18.2749	24.4725	Tropical and Subtropical Grasslands, Savannas and Shrublands	Zambezian Baikiaea woodlands	Photograph	All	no data	Andrew Stein
Non-melanistic 20	*pardus*	Ghanzi, Botswana	-21.5383	21.5976	Tropical and Subtropical Grasslands, Savannas and Shrublands	Kalahari Acacia-Baikiaea woodlands	Photograph	All	no data	Andrew Stein
Non-melanistic 21	*pardus*	Kgalagadi Transfrontier Park, Botswana	-25.5575	20.7679	Deserts and Xeric Shrublands	Kalahari xeric savanna	Photograph	All	no data	Andrew Stein
Non-melanistic 22	*pardus*	Kgalagadi Transfrontier Park, Botswana	-24.9999	21.1966	Deserts and Xeric Shrublands	Kalahari xeric savanna	Photograph	All	no data	Andrew Stein
Non-melanistic 23	*pardus*	Kgalagadi Transfrontier Park, Botswana	-24.5926	20.3313	Deserts and Xeric Shrublands	Kalahari xeric savanna	Photograph	All	no data	Andrew Stein
Non-melanistic 24	*pardus*	Kgalagadi Transfrontier Park, Botswana	-24.5773	20.3227	Deserts and Xeric Shrublands	Kalahari xeric savanna	Photograph	All	no data	Andrew Stein
Non-melanistic 25	*pardus*	Okavango Delta, Botswana	-18.3912	23.2474	Tropical and Subtropical Grasslands, Savannas and Shrublands	Zambezian Baikiaea woodlands	Photograph	All	no data	Andrew Stein
Non-melanistic 26	*pardus*	Tsao, Botswana	-20.4123	21.4723	Tropical and Subtropical Grasslands, Savannas and Shrublands	Kalahari Acacia-Baikiaea woodlands	Photograph	All	2009	Andrew Stein
Non-melanistic 27	*pardus*	Tsao, Botswana	-20.4123	21.4723	Tropical and Subtropical Grasslands, Savannas and Shrublands	Kalahari Acacia-Baikiaea woodlands	Photograph	All	2009	Andrew Stein
Non-melanistic 28	*pardus*	Tsao, Botswana	-20.4123	21.4723	Tropical and Subtropical Grasslands, Savannas and Shrublands	Kalahari Acacia-Baikiaea woodlands	Photograph	All	2009	Andrew Stein
Non-melanistic 29	*pardus*	Tuli Reserve, Botswana	-21.9139	28.9188	Tropical and Subtropical Grasslands, Savannas and Shrublands	Zambezian and Mopane woodlands	Photograph	All	no data	Andrew Stein
Non-melanistic 30	*delacouri*	Mondulkiri, Cambodia	12.4594	107.3662	Tropical and Subtropical Dry Broadleaf Forests	Southeastern Indochina dry evergreen forests	Photograph	All	2009	Bruce Kekule
Non-melanistic 31	*delacouri*	Mondulkiri Eastern Plains, Cambodia	12.5022	107.5279	Tropical and Subtropical Dry Broadleaf Forests	Southeastern Indochina dry evergreen forests	Photograph	All	2009	Bruce Kekule
Non-melanistic 32	*delacouri*	Mondulkiri Protected Forest, Cambodia	12.7827	106.9304	Tropical and Subtropical Dry Broadleaf Forests	Central Indochina dry forests	Photograph	All	no data	Bruce Kekule
Non-melanistic 33	*delacouri*	Srepok Wilderness Area, Cambodia	13.0865	107.3496	Tropical and Subtropical Dry Broadleaf Forests	Central Indochina dry forests	Photograph	All	no data	Bruce Kekule
Non-melanistic 34	*pardus*	Meuban, Cameroon	2.4069	12.6924	Tropical and Subtropical Moist Broadleaf Forests	Northwestern Congolian lowland forests	Photograph	Filtered	1935	American Museum of Natural History USA
Non-melanistic 35	*pardus*	Ngoundi, Cameroon	3.8528	15.1198	Tropical and Subtropical Moist Broadleaf Forests	Northwestern Congolian lowland forests	Photograph	Filtered	1935	American Museum of Natural History USA
Non-melanistic 36	*orientalis*	Duhuangzi, China	43.3754	130.8418	Temperate Broadleaf and Mixed Forests	Manchurian mixed forests	Photograph	All	2011	Shu Jin Luo
Non-melanistic 37	*japonensis*	Fu Tan, Yen Ching Kao, Szechuan, China	31.1713	103.6034	Temperate Coniferous Forest	Hengduan Shan conifer forests	Photograph	Filtered	1922	American Museum of Natural History USA
Non-melanistic 38	*japonensis*	Fujian, China	26.1299	119.3211	Tropical and Subtropical Moist Broadleaf Forests	Southeast China-Hainan moist forests	Photograph	Filtered	1925	American Museum of Natural History USA
Non-melanistic 39	*japonensis*	Fuqing, Fujian, China	25.7475	119.3734	Tropical and Subtropical Moist Broadleaf Forests	Southeast China-Hainan moist forests	Photograph	Filtered	1916	American Museum of Natural History USA
Non-melanistic 40	*japonensis*	Futsing, Fukien Province, China	26.4773	119.2144	Tropical and Subtropical Moist Broadleaf Forests	Southeast China-Hainan moist forests	Photograph	All	no data	American Museum of Natural History USA
Non-melanistic 41	*orientalis*	Hunchun Amur Tiger National Nature Reserve, China	43.1131	130.5932	Temperate Broadleaf and Mixed Forests	Manchurian mixed forests	Photograph	All	2012	Wildlife Conservation Society
Non-melanistic 42	*orientalis*	Hunchun Amur Tiger National Nature Reserve, China	43.1737	130.6676	Temperate Broadleaf and Mixed Forests	Manchurian mixed forests	Photograph	All	2012	Wildlife Conservation Society
Non-melanistic 43	*japonensis*	Kuan Shien, Sichuan, China	28.5850	111.8209	Temperate Broadleaf and Mixed Forests	Changjiang Plain evergreen forests	Photograph	Filtered	1932	National Museum of Natural History USA
Non-melanistic 44	*fusca*	Lung, China	31.0137	93.1163	Montane Grasslands and Shrublands	Southeast Tibet shrublands and meadows	Photograph	Filtered	1948	American Museum of Natural History USA
Non-melanistic 45	*fusca*	Mengmang, China	30.7464	93.1432	Montane Grasslands and Shrublands	Southeast Tibet shrublands and meadows	Photograph	All	no data	American Museum of Natural History USA
Non-melanistic 46	*orientalis*	Mijang, China	43.1356	130.2399	Temperate Broadleaf and Mixed Forests	Manchurian mixed forests	Photograph	All	2013	Wildlife Conservation Society
Non-melanistic 47	*japonensis*	Minchou, Gansu, China	26.2164	119.0907	Tropical and Subtropical Moist Broadleaf Forests	Southeast China-Hainan moist forests	Photograph	Filtered	1911	National Museum of Natural History USA
Non-melanistic 48	*japonensis*	Nanping, Fujian Province, China	26.6246	118.1872	Tropical and Subtropical Moist Broadleaf Forests	Southeast China-Hainan moist forests	Photograph	Filtered	1920	American Museum of Natural History USA
Non-melanistic 49	*japonensis*	Shansi, Hezhou, China	23.9024	111.7892	Tropical and Subtropical Moist Broadleaf Forests	Southeast China-Hainan moist forests	Photograph	All	no data	Asian Leopard Project
Non-melanistic 50	*japonensis*	Shanxi Province, China	37.8071	114.2488	Temperate Broadleaf and Mixed Forests	Central China loess plateau mixed forests	Photograph	All	no data	Asian Leopard Project
Non-melanistic 51	*japonensis*	Shanxi Province, China	37.5338	114.0875	Temperate Broadleaf and Mixed Forests	Central China loess plateau mixed forests	Photograph	All	no data	Asian Leopard Project

Id	Subspecies	Location	Deg. - WGS84		Biome	Ecorregion	Sample	Analysis	Year	Source
			Latitude	Longitude						
Non-melanistic 52	*fusca*	Sichuan, China	33.2854	98.7018	Montane Grasslands and Shrublands	Southeast Tibet shrublands and meadows	Photograph	All	2008	Smithsonian Institution
Non-melanistic 53	*japonensis*	Suifu, Tseo-Jia-Keo, Sichuan, China	28.7546	104.7215	Temperate Broadleaf and Mixed Forests	Sichuan Basin evergreen broadleaf forests	Photograph	Filtered	1927	National Museum of Natural History USA
Non-melanistic 54	*japonensis*	Suifu, Tseo-Jia-Keo, Sichuan, China	28.6382	104.7183	Temperate Broadleaf and Mixed Forests	Sichuan Basin evergreen broadleaf forests	Photograph	Filtered	1929	National Museum of Natural History USA
Non-melanistic 55	*japonensis*	Suifu, Tseo-Jia-Keo, Sichuan, China	28.7636	104.7596	Temperate Broadleaf and Mixed Forests	Sichuan Basin evergreen broadleaf forests	Photograph	Filtered	1929	National Museum of Natural History USA
Non-melanistic 56	*japonensis*	Tai-Yuan-Fu, Shanxi, China	38.0998	113.3001	Temperate Broadleaf and Mixed Forests	Central China loess plateau mixed forests	Photograph	Filtered	1910	National Museum of Natural History USA
Non-melanistic 57	*japonensis*	Tashenlu, Sichuan, China	29.5977	111.9222	Tropical and Subtropical Moist Broadleaf Forests	Guizhou Plateau broadleaf and mixed forests	Photograph	Filtered	1930	National Museum of Natural History USA
Non-melanistic 58	*japonensis*	Tseo-Jia-Keo, Sichuan, China	29.1496	112.2255	Temperate Broadleaf and Mixed Forests	Changjiang Plain evergreen forests	Photograph	Filtered	1931	National Museum of Natural History USA
Non-melanistic 59	*japonensis*	Wen Chuan, Sichuan, China	31.4709	103.5986	Temperate Coniferous Forest	Hengduan Shan conifer forests	Photograph	All	no data	National Museum of Natural History USA
Non-melanistic 60	*japonensis*	Yenping, Fukien Province, China	26.5698	118.5703	Tropical and Subtropical Moist Broadleaf Forests	Southeast China-Hainan moist forests	Photograph	All	1921	American Museum of Natural History USA
Non-melanistic 61	*japonensis*	Yochow, Hunan, China	28.1421	112.8030	Temperate Broadleaf and Mixed Forests	Changjiang Plain evergreen forests	Photograph	All	no data	National Museum of Natural History USA
Non-melanistic 62	*delacouri*	Yunnan National Nature Reserve, China	22.1923	101.3011	Tropical and Subtropical Moist Broadleaf Forests	North Indochina subtropical moist forests	Photograph	All	2008	Jutzeler et al 2010
Non-melanistic 63	*pardus*	Akenge, Congo	2.8334	27.1776	Tropical and Subtropical Moist Broadleaf Forests	Northeastern Congo Basin moist forests	Photograph	Filtered	1913	American Museum of Natural History USA
Non-melanistic 64	*pardus*	Akenge, Congo	2.8724	27.2210	Tropical and Subtropical Moist Broadleaf Forests	Northeastern Congo Basin moist forests	Photograph	Filtered	1913	American Museum of Natural History USA
Non-melanistic 65	*pardus*	Bwera, Congo	-0.8704	29.3107	Tropical and Subtropical Grasslands, Savannas and Shrublands	Victoria Basin forest-savanna mosaic	Photograph	All	2012	National Geographic Society
Non-melanistic 66	*pardus*	Congo River, Ngabe, Congo	-3.0253	16.1324	Tropical and Subtropical Grasslands, Savannas and Shrublands	Western Congolian forest-savanna mosaic	Photograph	All	no data	Philipp Henschel
Non-melanistic 67	*pardus*	Nouabalé-Ndoki National Park, Congo	2.4665	16.5519	Tropical and Subtropical Moist Broadleaf Forests	Western Congo Basin moist forests	Photograph	All	no data	Philipp Henschel
Non-melanistic 68	*pardus*	Bereket Girma Wildlife Rascue, Ethiopia	9.0639	38.5470	Montane Grasslands and Shrublands	Ethiopian Highlands	Photograph	All	2011	Stephen Brend
Non-melanistic 69	*pardus*	Haro, Abyssinia, Ethiopia	9.0104	34.7012	Tropical and Subtropical Moist Broadleaf Forests	Ethiopian montane forests	Photograph	All	no data	American Museum of Natural History USA
Non-melanistic 70	*pardus*	Ivindo National Park, Gabon	0.1768	12.9879	Tropical and Subtropical Moist Broadleaf Forests	Western Congo Basin moist forests	Photograph	All	2009	Philipp Henschel
Non-melanistic 71	*pardus*	Ivindo National Park, Gabon	0.1768	12.9879	Tropical and Subtropical Moist Broadleaf Forests	Western Congo Basin moist forests	Photograph	All	2009	Philipp Henschel
Non-melanistic 72	*pardus*	Ivindo National Park, Gabon	0.1768	12.9879	Tropical and Subtropical Moist Broadleaf Forests	Western Congo Basin moist forests	Photograph	All	2009	Philipp Henschel
Non-melanistic 73	*pardus*	Ivindo National Park, Gabon	-0.2699	12.7138	Tropical and Subtropical Moist Broadleaf Forests	Western Congo Basin moist forests	Photograph	All	no data	Philipp Henschel
Non-melanistic 74	*pardus*	Koulamoutou, Gabon	-0.9773	12.3016	Tropical and Subtropical Moist Broadleaf Forests	Western Congo Basin moist forests	Photograph	All	no data	Philipp Henschel
Non-melanistic 75	*pardus*	Lope National Park, Gabon	-0.2151	11.5191	Tropical and Subtropical Grasslands, Savannas and Shrublands	Western Congolian forest-savanna mosaic	Photograph	All	no data	Philipp Henschel
Non-melanistic 76	*pardus*	Lope National Park, Gabon	-0.2151	11.5191	Tropical and Subtropical Grasslands, Savannas and Shrublands	Western Congolian forest-savanna mosaic	Photograph	All	no data	Philipp Henschel
Non-melanistic 77	*pardus*	Lope National Park, Gabon	-0.5203	11.4897	Tropical and Subtropical Moist Broadleaf Forests	Congolian Coastal Forests	Photograph	All	no data	Philipp Henschel
Non-melanistic 78	*pardus*	Lope National Park, Gabon	-0.5203	11.4897	Tropical and Subtropical Moist Broadleaf Forests	Congolian Coastal Forests	Photograph	All	no data	Philipp Henschel
Non-melanistic 79	*pardus*	Lope National Park, Gabon	-0.6079	11.6042	Tropical and Subtropical Moist Broadleaf Forests	Congolian Coastal Forests	Photograph	All	no data	Philipp Henschel
Non-melanistic 80	*pardus*	Lope National Park, Gabon	-0.6606	11.5551	Tropical and Subtropical Moist Broadleaf Forests	Congolian Coastal Forests	Photograph	All	no data	Philipp Henschel
Non-melanistic 81	*pardus*	Lope National Park, Gabon	-0.1773	11.4754	Tropical and Subtropical Grasslands, Savannas and Shrublands	Western Congolian forest-savanna mosaic	Photograph	All	no data	Philipp Henschel
Non-melanistic 82	*pardus*	Lope National Park, Gabon	-0.3088	11.6332	Tropical and Subtropical Moist Broadleaf Forests	Congolian Coastal Forests	Photograph	All	no data	Philipp Henschel
Non-melanistic 83	*pardus*	Ogooue River, Kan Kan, Gabon	-0.0333	12.3136	Tropical and Subtropical Moist Broadleaf Forests	Western Congo Basin moist forests	Photograph	All	no data	Philipp Henschel
Non-melanistic 84	*pardus*	Ongongo, Gabon	1.3084	11.6978	Tropical and Subtropical Moist Broadleaf Forests	Congolian Coastal Forests	Photograph	All	no data	Philipp Henschel
Non-melanistic 85	*pardus*	Plateau Bateke National Park, Gabon	-2.2737	14.0952	Tropical and Subtropical Grasslands, Savannas and Shrublands	Western Congolian forest-savanna mosaic	Photograph	All	no data	Philipp Henschel
Non-melanistic 86	*saxicolor*	Vashlovani Reserve, Georgia	41.2115	46.4417	Deserts and Xeric Shrublands	Azerbaijan shrub desert and steppe	Photograph	All	no data	Asian Leopard Project
Non-melanistic 87	*pardus*	Mole National Park, Ghana	9.4323	-1.7133	Tropical and Subtropical Grasslands, Savannas and Shrublands	West Sudanian savanna	Photograph	All	no data	National Geographic Society
Non-melanistic 88	*pardus*	Mole National Park, Ghana	9.3845	-2.0271	Tropical and Subtropical Grasslands, Savannas and Shrublands	West Sudanian savanna	Photograph	All	no data	National Geographic Society
Non-melanistic 89	*fusca*	Achanakmar Tiger Reserve, India	22.5980	81.8327	Tropical and Subtropical Moist Broadleaf Forests	Eastern highlands moist deciduous forests	Photograph	All	no data	Vidya Athreya
Non-melanistic 90	*fusca*	Achanakmar Tiger Reserve, India	22.4232	81.7286	Tropical and Subtropical Moist Broadleaf Forests	Eastern highlands moist deciduous forests	Photograph	All	no data	Vidya Athreya
Non-melanistic 91	*fusca*	Akola/Rajur, India	19.5507	73.9639	Deserts and Xeric Shrublands	Deccan thorn scrub forests	Photograph	All	no data	Vidya Athreya
Non-melanistic 92	*fusca*	Akole, India	19.3109	73.4020	Tropical and Subtropical Moist Broadleaf Forests	North Western Ghats montane rain forests	Photograph	All	2012	Vidya Athreya
Non-melanistic 93	*fusca*	Akole, India	19.3258	73.3799	Tropical and Subtropical Moist Broadleaf Forests	North Western Ghats montane rain forests	Photograph	All	2012	Vidya Athreya
Non-melanistic 94	*fusca*	Anamalai Hills, Southern Western Ghats, India	12.1058	78.9328	Tropical and Subtropical Dry Broadleaf Forests	South Deccan Plateau dry deciduous forests	Photograph	All	no data	World Wild Fund
Non-melanistic 95	*fusca*	Bagdodra, Haskhowa, India	26.7458	88.2892	Tropical and Subtropical Moist Broadleaf Forests	Himalayan subtropical broadleaf forests	Photograph	All	2012	World Wild Fund
Non-melanistic 96	*fusca*	Bandipur Tiger Reserve, India	11.6407	76.4442	Tropical and Subtropical Moist Broadleaf Forests	Southwestern Ghats moist forest	Photograph	All	2013	Wildlife Conservation Society
Non-melanistic 97	*fusca*	Bhadra Tiger Reserve, India	13.6071	75.5910	Tropical and Subtropical Moist Broadleaf Forests	North Western Ghats moist deciduous forests	Photograph	All	no data	Vidya Athreya
Non-melanistic 98	*fusca*	Bhadra Tiger Reserve, India	13.5380	75.5222	Tropical and Subtropical Moist Broadleaf Forests	North Western Ghats moist deciduous forests	Photograph	All	2011	Conservation India
Non-melanistic 99	*fusca*	Bhadra Tiger Reserve, India	13.6189	75.6286	Tropical and Subtropical Moist Broadleaf Forests	North Western Ghats moist deciduous forests	Photograph	All	2012	Conservation India
Non-melanistic 100	*fusca*	Dahra Dan, India	30.3872	78.1732	Tropical and Subtropical Moist Broadleaf Forests	Upper Gangetic Plains moist deciduous forests	Photograph	All	no data	Goyal 2009
Non-melanistic 101	*fusca*	Dandeli-Anshi Tiger Reserve, India	13.1213	75.0433	Tropical and Subtropical Moist Broadleaf Forests	Malabar Coast moist forests	Photograph	All	2013	Wildlife Conservation Society
Non-melanistic 102	*fusca*	Dandeli-Anshi Tiger Reserve, India	13.1213	75.0433	Tropical and Subtropical Moist Broadleaf Forests	Malabar Coast moist forests	Photograph	All	2013	Wildlife Conservation Society
Non-melanistic 103	*fusca*	Dehing Patkai Wildlife Sanctuary, India	26.4451	93.5389	Tropical and Subtropical Moist Broadleaf Forests	Meghalaya subtropical forests	Photograph	All	2009	Dipankar Ghose
Non-melanistic 104	*fusca*	Dudhwa National Park, India	27.4659	79.7461	Tropical and Subtropical Moist Broadleaf Forests	Upper Gangetic Plains moist deciduous forests	Photograph	All	no data	World Wild Fund
Non-melanistic 105	*fusca*	Dudhwa National Park, India	27.2008	79.8949	Tropical and Subtropical Moist Broadleaf Forests	Upper Gangetic Plains moist deciduous forests	Photograph	All	no data	World Wild Fund
Non-melanistic 106	*fusca*	Dudhwa National Park, India	27.9333	81.3510	Tropical and Subtropical Moist Broadleaf Forests	Upper Gangetic Plains moist deciduous forests	Photograph	All	no data	World Wild Fund
Non-melanistic 107	*fusca*	Garhwal Western Himalaya, India	30.5542	79.3165	Montane Grasslands and Shrublands	Western Himalayan alpine shrub and Meadows	Photograph	All	2008	World Wild Fund
Non-melanistic 108	*fusca*	Gir Forest National Park, India	21.2057	71.1518	Tropical and Subtropical Dry Broadleaf Forests	Khathiar-Gir dry deciduous forests	Photograph	All	no data	Singh 2005
Non-melanistic 109	*fusca*	Gir Forest National Park, India	21.2721	71.1679	Tropical and Subtropical Dry Broadleaf Forests	Khathiar-Gir dry deciduous forests	Photograph	All	no data	Smithsonian Institution
Non-melanistic 110	*fusca*	Guwahati, India	26.1208	91.7107	Tropical and Subtropical Moist Broadleaf Forests	Meghalaya subtropical forests	Photograph	All	no data	Dipankar Ghose
Non-melanistic 111	*fusca*	Haraicha, Mavatuv, India	30.7922	76.1306	Deserts and Xeric Shrublands	Northwestern thorn scrub forests	Photograph	Filtered	1936	American Museum of Natural History USA
Non-melanistic 112	*fusca*	Haridwar, Rajaji National Park Chila Range, India	30.0489	78.3142	Tropical and Subtropical Moist Broadleaf Forests	Upper Gangetic Plains moist deciduous forests	Photograph	All	no data	Smithsonian Institution

Id	Subspecies	Location	Deg. - WGS84		Biome	Ecorregion	Sample	Analysis	Year	Source
			Latitude	Longitude						
Non-melanistic 113	*fusca*	Hyhama, Jammu And Kashmir, India	33.9766	77.4887	Montane Grasslands and Shrublands	Tibetan Plateau steppe	Photograph	Filtered	1911	National Museum of Natural History USA
Non-melanistic 114	*fusca*	Hyhama, Jammu And Kashmir, India	33.6569	77.7122	Montane Grasslands and Shrublands	Tibetan Plateau steppe	Photograph	Filtered	1911	National Museum of Natural History USA
Non-melanistic 115	*fusca*	Jeypore-Dehing Area, India	27.1370	95.3860	Tropical and Subtropical Moist Broadleaf Forests	Brahmaputra Valley semi-evergreen forests	Photograph	All	no data	Kashmira Kakati
Non-melanistic 116	*fusca*	Jeypore-Dehing Area, India	27.1370	95.3860	Tropical and Subtropical Moist Broadleaf Forests	Brahmaputra Valley semi-evergreen forests	Photograph	All	no data	Kashmira Kakati
Non-melanistic 117	*fusca*	Jeypore-Dehing Area, India	27.1370	95.3860	Tropical and Subtropical Moist Broadleaf Forests	Brahmaputra Valley semi-evergreen forests	Photograph	All	no data	Kashmira Kakati
Non-melanistic 118	*fusca*	Jeypore-Dehing Area, India	27.0584	95.5366	Tropical and Subtropical Moist Broadleaf Forests	Brahmaputra Valley semi-evergreen forests	Photograph	All	no data	Kashmira Kakati
Non-melanistic 119	*fusca*	Jeypore-Dehing Area, India	27.2488	95.5193	Tropical and Subtropical Moist Broadleaf Forests	Brahmaputra Valley semi-evergreen forests	Photograph	All	no data	Kashmira Kakati
Non-melanistic 120	*fusca*	Jeypore-Dehing Area, India	27.2488	95.5193	Tropical and Subtropical Moist Broadleaf Forests	Brahmaputra Valley semi-evergreen forests	Photograph	All	no data	Kashmira Kakati
Non-melanistic 121	*fusca*	Jeypore-Dehing Area, India	27.6499	95.4474	Tropical and Subtropical Moist Broadleaf Forests	Brahmaputra Valley semi-evergreen forests	Photograph	All	no data	Kashmira Kakati
Non-melanistic 122	*fusca*	Jeypore-Dehing Area, India	27.1285	95.8475	Temperate Broadleaf and Mixed Forests	Eastern Himalayan broadleaf forests	Photograph	All	no data	Kashmira Kakati
Non-melanistic 123	*fusca*	Jeypore-Dehing Area, India	27.1285	95.8475	Temperate Broadleaf and Mixed Forests	Eastern Himalayan broadleaf forests	Photograph	All	no data	Kashmira Kakati
Non-melanistic 124	*fusca*	Jeypore-Dehing Area, India	27.2331	95.6986	Tropical and Subtropical Moist Broadleaf Forests	Brahmaputra Valley semi-evergreen forests	Photograph	All	no data	Kashmira Kakati
Non-melanistic 125	*fusca*	Jeypore-Dehing Area, India	27.2331	95.6986	Tropical and Subtropical Moist Broadleaf Forests	Brahmaputra Valley semi-evergreen forests	Photograph	All	no data	Kashmira Kakati
Non-melanistic 126	*fusca*	Jeypore-Dehing Area, India	27.3618	95.7520	Tropical and Subtropical Moist Broadleaf Forests	Brahmaputra Valley semi-evergreen forests	Photograph	All	no data	Kashmira Kakati
Non-melanistic 127	*fusca*	Jeypore-Dehing Area, India	27.3618	95.7520	Tropical and Subtropical Moist Broadleaf Forests	Brahmaputra Valley semi-evergreen forests	Photograph	All	no data	Kashmira Kakati
Non-melanistic 128	*fusca*	Jeypore-Dehing Area, India	27.4243	95.7601	Tropical and Subtropical Moist Broadleaf Forests	Brahmaputra Valley semi-evergreen forests	Photograph	All	no data	Kashmira Kakati
Non-melanistic 129	*fusca*	Jeypore-Dehing Area, India	27.4243	95.7601	Tropical and Subtropical Moist Broadleaf Forests	Brahmaputra Valley semi-evergreen forests	Photograph	All	no data	Kashmira Kakati
Non-melanistic 130	*fusca*	Jeypore-Dehing Area, India	27.5402	95.7905	Tropical and Subtropical Moist Broadleaf Forests	Brahmaputra Valley semi-evergreen forests	Photograph	All	no data	Kashmira Kakati
Non-melanistic 131	*fusca*	Jeypore-Dehing Area, India	27.5402	95.7905	Tropical and Subtropical Moist Broadleaf Forests	Brahmaputra Valley semi-evergreen forests	Photograph	All	no data	Kashmira Kakati
Non-melanistic 132	*fusca*	Jeypore-Dehing Area, India	27.5402	95.7905	Tropical and Subtropical Moist Broadleaf Forests	Brahmaputra Valley semi-evergreen forests	Photograph	All	no data	Kashmira Kakati
Non-melanistic 133	*fusca*	Kabini, Bandipur, India	34.1378	75.0364	Temperate Broadleaf and Mixed Forests	Western Himalayan broadleaf forests	Photograph	All	no data	Vidya Athreya
Non-melanistic 134	*fusca*	Karnataka, India	12.8232	76.0097	Tropical and Subtropical Moist Broadleaf Forests	North Western Ghats moist deciduous forests	Photograph	All	2013	World Wild Fund
Non-melanistic 135	*fusca*	Kanavde, India	19.5468	74.0299	Deserts and Xeric Shrublands	Deccan thorn scrub forests	Photograph	All	no data	Vidya Athreya
Non-melanistic 136	*fusca*	Kanha Tiger Reserve, Madhya Pradesh, India	23.2809	80.4837	Tropical and Subtropical Moist Broadleaf Forests	Eastern Deccan plateau moist forests	Photograph	All	no data	Christoph Knogge
Non-melanistic 137	*fusca*	Kanha Tiger Reserve, Madhya Pradesh, India	23.0263	80.7801	Tropical and Subtropical Moist Broadleaf Forests	Eastern Deccan plateau moist forests	Photograph	All	2013	Sandeep Sharma
Non-melanistic 138	*fusca*	Knagar, India	19.5177	74.0293	Deserts and Xeric Shrublands	Deccan thorn scrub forests	Photograph	All	no data	Vidya Athreya
Non-melanistic 139	*fusca*	Kormar Gudda, India	22.9019	81.1676	Tropical and Subtropical Moist Broadleaf Forests	Eastern Deccan plateau moist forests	Photograph	All	no data	Kashmira Kakati
Non-melanistic 140	*fusca*	Kundur, India	23.4147	80.7939	Tropical and Subtropical Moist Broadleaf Forests	Eastern Deccan plateau moist forests	Photograph	All	no data	Kashmira Kakati
Non-melanistic 141	*fusca*	Kupwara Forest, India	34.5811	74.3511	Temperate Broadleaf and Mixed Forests	Western Himalayan broadleaf forests	Photograph	All	no data	Kashmira Kakati
Non-melanistic 142	*fusca*	Madras, Mavatuv, India	31.4190	76.5938	Tropical and Subtropical Coniferous Forests	Himalayan subtropical pine forests	Photograph	Filtered	1936	American Museum of Natural History USA
Non-melanistic 143	*fusca*	Mundanthurai Sanctuary, India	8.7603	77.2816	Tropical and Subtropical Moist Broadleaf Forests	Southwestern Ghats moist forest	Photograph	All	no data	Christoph Knogge
Non-melanistic 144	*fusca*	Mundanthurai Sanctuary, India	8.7252	77.2885	Tropical and Subtropical Moist Broadleaf Forests	Southwestern Ghats moist forest	Photograph	All	no data	Christoph Knogge
Non-melanistic 145	*fusca*	Nagarjunasagar Srisailam Tiger Reserve, india	16.4090	79.2564	Tropical and Subtropical Dry Broadleaf Forests	Central Deccan Plateau dry deciduous forests	Photograph	All	2011	World Wild Fund
Non-melanistic 146	*fusca*	Naimeri National Park, India	26.9456	92.7525	Tropical and Subtropical Moist Broadleaf Forests	Brahmaputra Valley semi-evergreen forests	Photograph	All	2011	World Wild Fund
Non-melanistic 147	*fusca*	Pauri Garhwal, India	29.8702	78.9477	Tropical and Subtropical Coniferous Forests	Himalayan subtropical pine forests	Photograph	All	no data	Goyal 2009
Non-melanistic 148	*fusca*	Pakke Tiger Reserve in Arunachal Pradesh, India	28.5669	95.9691	Temperate Broadleaf and Mixed Forests	Eastern Himalayan broadleaf forests	Photograph	All	2013	Milind Pariwakam/Biswajit Mohanty
Non-melanistic 149	*fusca*	Philibit, India	28.6421	79.9662	Tropical and Subtropical Moist Broadleaf Forests	Upper Gangetic Plains moist deciduous forests	Photograph	All	2010	World Wild Fund
Non-melanistic 150	*fusca*	Pilibhit, India	28.6355	79.8480	Tropical and Subtropical Moist Broadleaf Forests	Upper Gangetic Plains moist deciduous forests	Photograph	All	2010	World Wild Fund
Non-melanistic 151	*fusca*	Sanaripur, India	30.0183	77.5406	Tropical and Subtropical Moist Broadleaf Forests	Upper Gangetic Plains moist deciduous forests	Photograph	Filtered	1923	American Museum of Natural History USA
Non-melanistic 152	*fusca*	Sanaripur, India	30.0183	77.5406	Tropical and Subtropical Moist Broadleaf Forests	Upper Gangetic Plains moist deciduous forests	Photograph	Filtered	1923	American Museum of Natural History USA
Non-melanistic 153	*fusca*	Sanaripur, India	29.9502	77.5913	Tropical and Subtropical Moist Broadleaf Forests	Upper Gangetic Plains moist deciduous forests	Photograph	Filtered	1923	American Museum of Natural History USA
Non-melanistic 154	*fusca*	Sanjay Gandhi National Park, India	19.2325	72.9024	Tropical and Subtropical Moist Broadleaf Forests	Malabar Coast moist forests	Photograph	All	2012	Conservation India
Non-melanistic 155	*fusca*	Sanjay Gandhi National Park, India	19.2141	72.9253	Tropical and Subtropical Moist Broadleaf Forests	Malabar Coast moist forests	Photograph	All	2013	Conservation India
Non-melanistic 156	*fusca*	Sanjay Gandhi National Park, India	19.1726	72.8914	Tropical and Subtropical Moist Broadleaf Forests	Malabar Coast moist forests	Photograph	All	2013	Conservation India
Non-melanistic 157	*fusca*	Sanjay Gandhi National Park, India	19.1726	72.8914	Tropical and Subtropical Moist Broadleaf Forests	Malabar Coast moist forests	Photograph	All	2013	Conservation India
Non-melanistic 158	*fusca*	Sariska Tiger Reserve, India	27.4076	76.7404	Tropical and Subtropical Dry Broadleaf Forests	Khathiar-Gir dry deciduous forests	Photograph	All	no data	National Geographic Society
Non-melanistic 159	*fusca*	Sariska Tiger Reserve, India	27.3019	76.7276	Tropical and Subtropical Dry Broadleaf Forests	Khathiar-Gir dry deciduous forests	Photograph	All	no data	National Geographic Society
Non-melanistic 160	*fusca*	Sariska Tiger Reserve, India	27.2146	76.4171	Tropical and Subtropical Dry Broadleaf Forests	Khathiar-Gir dry deciduous forests	Photograph	All	no data	Chauhan et al 2005
Non-melanistic 161	*fusca*	Sariska Tiger Reserve, India	27.2146	76.4171	Tropical and Subtropical Dry Broadleaf Forests	Khathiar-Gir dry deciduous forests	Photograph	All	no data	Chauhan et al 2005
Non-melanistic 162	*fusca*	Sariska Tiger Reserve, India	27.2426	76.3155	Tropical and Subtropical Dry Broadleaf Forests	Khathiar-Gir dry deciduous forests	Photograph	All	no data	Chauhan et al 2005
Non-melanistic 163	*fusca*	Sariska Tiger Reserve, India	27.1166	76.2561	Tropical and Subtropical Dry Broadleaf Forests	Khathiar-Gir dry deciduous forests	Photograph	All	no data	Chauhan et al 2005
Non-melanistic 164	*fusca*	Sariska Tiger Reserve, India	27.3059	76.4886	Tropical and Subtropical Dry Broadleaf Forests	Khathiar-Gir dry deciduous forests	Photograph	All	no data	Chauhan et al 2005
Non-melanistic 165	*fusca*	Shivaji, India	19.5854	73.9862	Deserts and Xeric Shrublands	Deccan thorn scrub forests	Photograph	All	no data	Vidya Athreya
Non-melanistic 166	*fusca*	Siliguri, Prakash Nagar, India	26.8001	88.4827	Tropical and Subtropical Grasslands, Savannas and Shrublands	Terai-Duar savanna and grasslands	Photograph	All	2011	Vidya Athreya
Non-melanistic 167	*fusca*	Tadoba Andhari Tiger Reserve, Maharashtra, India	20.2085	79.5276	Tropical and Subtropical Dry Broadleaf Forests	Central Deccan Plateau dry deciduous forests	Photograph	All	no data	Vidya Athreya
Non-melanistic 168	*fusca*	Talewadi, India	19.5107	73.9672	Deserts and Xeric Shrublands	Deccan thorn scrub forests	Photograph	All	no data	Vidya Athreya
Non-melanistic 169	*fusca*	Uttarakhand, India	30.0002	80.2295	Temperate Broadleaf and Mixed Forests	Western Himalayan broadleaf forests	Photograph	All	no data	Goyal 2009
Non-melanistic 170	*fusca*	Uttarakhand, India	30.3109	79.6338	Montane Grasslands and Shrublands	Western Himalayan alpine shrub and Meadows	Photograph	All	no data	Goyal 2009
Non-melanistic 171	*fusca*	Uttarakhand, India	30.4446	79.6386	Montane Grasslands and Shrublands	Western Himalayan alpine shrub and Meadows	Photograph	All	no data	Goyal 2009
Non-melanistic 172	*fusca*	Uttarakhand, India	29.9234	79.6530	Tropical and Subtropical Coniferous Forests	Himalayan subtropical pine forests	Photograph	All	no data	Goyal 2009
Non-melanistic 173	*fusca*	Uttaranchal, India	30.3076	80.1796	Montane Grasslands and Shrublands	Western Himalayan alpine shrub and Meadows	Photograph	All	no data	Marker & Sivamani 2009

Id	Subspecies	Location	Deg. - WGS84		Biome	Ecorregion	Sample	Analysis	Year	Source
			Latitude	Longitude						
Non-melanistic 174	*fusca*	Vitbhatti, India	19.5298	74.0396	Deserts and Xeric Shrublands	Deccan thorn scrub forests	Photograph	All	no data	Vidya Athreya
Non-melanistic 175	*melas*	Baluran National Park, Java, Indonesia	-7.8044	114.3797	Tropical and Subtropical Moist Broadleaf Forests	Eastern Java-Bali rain forests	Photograph	All	2012	Copenhague Zoo
Non-melanistic 176	*melas*	Baluran National Park, Java, Indonesia	-7.8878	114.3786	Tropical and Subtropical Moist Broadleaf Forests	Eastern Java-Bali rain forests	Photograph	All	2012	Copenhague Zoo
Non-melanistic 177	*melas*	Bengkung, Java, Indonesia	-6.7706	106.4565	Tropical and Subtropical Moist Broadleaf Forests	Western Java rain forests	Photograph	All	no data	WildCru
Non-melanistic 178	*melas*	Gunung Salak National Park, Java, Indonesia	-7.2547	107.4355	Tropical and Subtropical Moist Broadleaf Forests	Western Java rain forests	Photograph	All	no data	CIFOR
Non-melanistic 179	*melas*	Halimun-Salak, Java, Indonesia	-6.7847	106.5780	Tropical and Subtropical Moist Broadleaf Forests	Western Java rain forests	Photograph	All	2004	Anhar Harahap
Non-melanistic 180	*melas*	Mount Halimun-Salak National Park, Java, Indonesia	-7.1029	107.3755	Tropical and Subtropical Moist Broadleaf Forests	Western Java rain forests	Photograph	All	no data	CIFOR
Non-melanistic 181	*melas*	Pelaboean Ratoe, Java, Indonesia	-6.9882	106.5549	Tropical and Subtropical Moist Broadleaf Forests	Western Java rain forests	Photograph	All	1909	National Museum of Natural History USA
Non-melanistic 182	*melas*	Ujung Kulon National Park, Java, Indonesia	-6.7339	105.3438	Tropical and Subtropical Moist Broadleaf Forests	Western Java rain forests	Photograph	All	no data	WildCru
Non-melanistic 183	*saxicolor*	Alborz Mountains, Iran	36.4611	51.4824	Temperate Broadleaf and Mixed Forests	Caspian Hyrcanian mixed forests	Photograph	All	no data	Farhadinia et al 2007
Non-melanistic 184	*saxicolor*	Bafq, Iran	31.8150	55.2717	Deserts and Xeric Shrublands	Central Persian desert basins	Photograph	All	2012	Asian Leopard Project
Non-melanistic 185	*saxicolor*	Bafq, Iran	31.7713	55.3136	Deserts and Xeric Shrublands	Central Persian desert basins	Photograph	All	2013	Panthera
Non-melanistic 186	*saxicolor*	Bafq, Iran	31.7713	55.3136	Deserts and Xeric Shrublands	Central Persian desert basins	Photograph	All	2013	Panthera
Non-melanistic 187	*saxicolor*	Bafq, Iran	32.2349	55.4353	Deserts and Xeric Shrublands	Central Persian desert basins	Photograph	All	2013	Panthera
Non-melanistic 188	*saxicolor*	Bafq, Iran	32.2349	55.4353	Deserts and Xeric Shrublands	Central Persian desert basins	Photograph	All	2013	Panthera
Non-melanistic 189	*saxicolor*	Bafq, Iran	31.6719	55.0649	Deserts and Xeric Shrublands	Central Persian desert basins	Photograph	All	2013	Panthera
Non-melanistic 190	*saxicolor*	Bamu National Park, Iran	29.6579	53.1614	Temperate Broadleaf and Mixed Forests	Zagros Mountains forest steppe	Photograph	All	no data	Arash Ghoddousi
Non-melanistic 191	*saxicolor*	Bamu National Park, Iran	29.8867	52.9034	Temperate Broadleaf and Mixed Forests	Zagros Mountains forest steppe	Photograph	All	no data	Arash Ghoddousi
Non-melanistic 192	*saxicolor*	Bamu National Park, Iran	29.8339	52.9008	Temperate Broadleaf and Mixed Forests	Zagros Mountains forest steppe	Photograph	All	no data	Arash Ghoddousi
Non-melanistic 193	*saxicolor*	Bamu National Park, Iran	29.8853	52.9552	Temperate Broadleaf and Mixed Forests	Zagros Mountains forest steppe	Photograph	All	no data	Arash Ghoddousi
Non-melanistic 194	*saxicolor*	Bamu National Park, Iran	29.8853	52.9552	Temperate Broadleaf and Mixed Forests	Zagros Mountains forest steppe	Photograph	All	no data	Arash Ghoddousi
Non-melanistic 195	*saxicolor*	Bamu National Park, Iran	29.8853	52.9552	Temperate Broadleaf and Mixed Forests	Zagros Mountains forest steppe	Photograph	All	no data	Arash Ghoddousi
Non-melanistic 196	*saxicolor*	Bamu National Park, Iran	29.8853	52.9552	Temperate Broadleaf and Mixed Forests	Zagros Mountains forest steppe	Photograph	All	no data	Arash Ghoddousi
Non-melanistic 197	*saxicolor*	Bamu National Park, Iran	29.8853	52.9552	Temperate Broadleaf and Mixed Forests	Zagros Mountains forest steppe	Photograph	All	no data	Arash Ghoddousi
Non-melanistic 198	*saxicolor*	Bamu National Park, Iran	29.8150	52.9750	Temperate Broadleaf and Mixed Forests	Zagros Mountains forest steppe	Photograph	All	no data	Arash Ghoddousi
Non-melanistic 199	*saxicolor*	Bandar-Gaz, Iran	26.6795	55.0549	Deserts and Xeric Shrublands	South Iran Nubo-Sindian desert and semi-desert	Photograph	All	no data	Asian Leopard Project
Non-melanistic 200	*saxicolor*	Birk Protected Area, Iran	29.6558	58.3126	Deserts and Xeric Shrublands	South Iran Nubo-Sindian desert and semi-desert	Photograph	All	no data	Asian Leopard Project
Non-melanistic 201	*saxicolor*	Birk Protected Area, Iran	29.5872	58.3913	Deserts and Xeric Shrublands	South Iran Nubo-Sindian desert and semi-desert	Photograph	All	no data	Asian Leopard Project
Non-melanistic 202	*saxicolor*	Dargaz, Khorasan Province, Iran	36.9854	58.6955	Deserts and Xeric Shrublands	Central Persian desert basins	Photograph	All	no data	Asian Leopard Project
Non-melanistic 203	*saxicolor*	Ghorkhod & Behkadeh Reserve, Iran	37.6015	56.5290	Temperate Broadleaf and Mixed Forests	Caucasus-Anatolian-Hyrcanian temperate forests	Photograph	All	no data	Farhadinia et al 2007
Non-melanistic 204	*saxicolor*	Golestan National Park, Iran	38.0852	56.4868	Montane Grasslands and Shrublands	Kopet Dag woodlands and forest steppe	Photograph	All	no data	Asian Leopard Project
Non-melanistic 205	*saxicolor*	Gorgan-Golestan, Iran	37.2019	54.6584	Deserts and Xeric Shrublands	Caspian lowland desert	Photograph	All	no data	Asian Leopard Project
Non-melanistic 206	*saxicolor*	Gorgan-Golestan, Iran	37.2709	54.3942	Deserts and Xeric Shrublands	Caspian lowland desert	Photograph	All	no data	Asian Leopard Project
Non-melanistic 207	*saxicolor*	Gouladah, Bujnurd, Iran	37.4797	57.2698	Deserts and Xeric Shrublands	Central Persian desert basins	Photograph	All	1938	American Museum of Natural History USA
Non-melanistic 208	*saxicolor*	Kerman, Iran	30.3739	57.3553	Montane Grasslands and Shrublands	Kuh Rud and Eastern Iran montane woodlands	Photograph	All	no data	Asian Leopard Project
Non-melanistic 209	*saxicolor*	Khaeez Area, Iran	28.7051	51.5107	Deserts and Xeric Shrublands	South Iran Nubo-Sindian desert and semi-desert	Photograph	All	no data	Abdoli et al 2008
Non-melanistic 210	*saxicolor*	Khojir National Park, Iran	35.5963	51.8186	Deserts and Xeric Shrublands	Central Persian desert basins	Photograph	All	no data	Asian Leopard Project
Non-melanistic 211	*saxicolor*	Khorasan Province, Iran	35.8595	60.1116	Deserts and Xeric Shrublands	Central Persian desert basins	Photograph	All	no data	Asian Leopard Project
Non-melanistic 212	*saxicolor*	Khorasan Province, Iran	33.2073	60.3131	Montane Grasslands and Shrublands	Kuh Rud and Eastern Iran montane woodlands	Photograph	All	no data	Asian Leopard Project
Non-melanistic 213	*saxicolor*	Khorasan Province, Iran	35.5268	59.2535	Montane Grasslands and Shrublands	Kuh Rud and Eastern Iran montane woodlands	Photograph	All	no data	Asian Leopard Project
Non-melanistic 214	*saxicolor*	Khorasan Province, Iran	34.2792	58.7812	Deserts and Xeric Shrublands	Central Persian desert basins	Photograph	All	no data	Asian Leopard Project
Non-melanistic 215	*saxicolor*	Khorasan Province, Iran	33.1146	59.2725	Montane Grasslands and Shrublands	Kuh Rud and Eastern Iran montane woodlands	Photograph	All	no data	Asian Leopard Project
Non-melanistic 216	*saxicolor*	Khorasan Province, Iran	35.6120	58.3048	Montane Grasslands and Shrublands	Kuh Rud and Eastern Iran montane woodlands	Photograph	All	no data	Asian Leopard Project
Non-melanistic 217	*saxicolor*	Khosh, Semnan Province, Iran	35.5550	55.3459	Deserts and Xeric Shrublands	Central Persian desert basins	Photograph	All	no data	Asian Leopard Project
Non-melanistic 218	*saxicolor*	Kiamaki Wildlife Reserve, Iran	38.7594	45.8547	Temperate Grasslands, Savannas and Shrublands	Eastern Anatolian montane steppe	Photograph	All	no data	Asian Leopard Project
Non-melanistic 219	*saxicolor*	Laristan, Bariz, Iran	33.5913	49.1922	Temperate Broadleaf and Mixed Forests	Zagros Mountains forest steppe	Photograph	All	1963	National Museum of Natural History USA
Non-melanistic 220	*saxicolor*	Laristan, Fars, Iran	33.3084	49.2420	Temperate Broadleaf and Mixed Forests	Zagros Mountains forest steppe	Photograph	All	1963	National Museum of Natural History USA
Non-melanistic 221	*saxicolor*	Mazandaran, Iran	36.3078	52.1673	Temperate Broadleaf and Mixed Forests	Caspian Hyrcanian mixed forests	Photograph	All	no data	Asian Leopard Project
Non-melanistic 222	*saxicolor*	Mazandaran, Iran	36.3296	53.0279	Temperate Broadleaf and Mixed Forests	Caspian Hyrcanian mixed forests	Photograph	All	no data	Asian Leopard Project
Non-melanistic 223	*saxicolor*	Neishabour, Khorasan Province, Iran	36.2544	58.8633	Temperate Coniferous Forest	Caucasus-Anatolian-Hyrcanian temperate forests	Photograph	All	no data	Asian Leopard Project
Non-melanistic 224	*saxicolor*	North Khorasan, Iran	35.8884	58.6493	Deserts and Xeric Shrublands	Central Persian desert basins	Photograph	All	2012	Asian Leopard Project
Non-melanistic 225	*saxicolor*	Qualanlu, Iran	37.5376	56.4123	Temperate Coniferous Forest	Caucasus-Anatolian-Hyrcanian temperate forests	Photograph	All	2013	Asian Leopard Project
Non-melanistic 226	*saxicolor*	Qualanlu, Iran	37.5425	56.1588	Temperate Broadleaf and Mixed Forests	Caspian Hyrcanian mixed forests	Photograph	All	2013	Asian Leopard Project
Non-melanistic 227	*saxicolor*	Sarigol National Park, Iran	37.8396	57.0308	Montane Grasslands and Shrublands	Kopet Dag woodlands and forest steppe	Photograph	All	2007	Farhadinia et al 2010
Non-melanistic 228	*saxicolor*	Sarigol National Park, Iran	37.8784	56.6037	Montane Grasslands and Shrublands	Kopet Dag woodlands and forest steppe	Photograph	All	2007	Farhadinia et al 2010
Non-melanistic 229	*saxicolor*	Talysh Mountains, Iran	38.8721	46.3442	Temperate Grasslands, Savannas and Shrublands	Eastern Anatolian montane steppe	Photograph	All	2007	Lukarevsky et al 2007
Non-melanistic 230	*saxicolor*	Tandoureh National Park, Iran	37.2770	58.4683	Montane Grasslands and Shrublands	Kopet Dag woodlands and forest steppe	Photograph	All	no data	Sayed Babak
Non-melanistic 231	*pardus*	Cheranganghi Hills, Kenya	-1.4972	36.6726	Tropical and Subtropical Grasslands, Savannas and Shrublands	East African Acacia Savannas	Photograph	All	no data	American Museum of Natural History USA
Non-melanistic 232	*pardus*	Cheranganghi Hills, Kenya	-1.6135	36.7174	Tropical and Subtropical Grasslands, Savannas and Shrublands	East African Acacia Savannas	Photograph	All	no data	American Museum of Natural History USA
Non-melanistic 233	*pardus*	Elgeyo Forest, Kenya	1.0730	35.2871	Tropical and Subtropical Moist Broadleaf Forests	East African montane forests	Photograph	All	no data	American Museum of Natural History USA
Non-melanistic 234	*pardus*	Endau, Kenya	-1.3345	38.6644	Tropical and Subtropical Grasslands, Savannas and Shrublands	East African Acacia Savannas	Photograph	All	no data	Smithsonian Institution

Id	Subspecies	Location	Deg. - WGS84		Biome	Ecoregion	Sample	Analysis	Year	Source
			Latitude	Longitude						
Non-melanistic 235	*pardus*	Ewaso Lions Camp, Kenya	-1.4226	36.8473	Tropical and Subtropical Grasslands, Savannas and Shrublands	East African Acacia Savannas	Photograph	All	no data	Phillip Henschel
Non-melanistic 236	*pardus*	Guaso Ngishu Plateau, Kenya	-1.1088	36.5234	Tropical and Subtropical Grasslands, Savannas and Shrublands	East African Acacia Savannas	Photograph	All	1909	National Museum of Natural History USA
Non-melanistic 237	*pardus*	Kampi Moto, Nakuru, Kenya	-0.4294	36.1020	Tropical and Subtropical Grasslands, Savannas and Shrublands	East African Acacia Savannas	Photograph	All	1909	National Museum of Natural History USA
Non-melanistic 238	*pardus*	Lake Naivasha, Kenya	-0.6986	36.3578	Tropical and Subtropical Grasslands, Savannas and Shrublands	East African Acacia Savannas	Photograph	All	1909	National Museum of Natural History USA
Non-melanistic 239	*pardus*	Lake Naivasha, S End, Kenya	-0.8683	36.2074	Tropical and Subtropical Grasslands, Savannas and Shrublands	East African Acacia Savannas	Photograph	All	1909	National Museum of Natural History USA
Non-melanistic 240	*pardus*	Masai Mara Game Reserve, Kenya	-1.4803	35.1052	Tropical and Subtropical Grasslands, Savannas and Shrublands	East African Acacia Savannas	Photograph	All	no data	Smithsonian Institution
Non-melanistic 241	*pardus*	Magadi, Kenya	-1.7446	36.3396	Tropical and Subtropical Grasslands, Savannas and Shrublands	East African Acacia Savannas	Photograph	All	2010	Smithsonian Institution
Non-melanistic 242	*pardus*	Magadi, Kenya	-1.7837	36.3508	Tropical and Subtropical Grasslands, Savannas and Shrublands	East African Acacia Savannas	Photograph	All	2010	Smithsonian Institution
Non-melanistic 243	*pardus*	Masai Mara National Park, Kenya	-1.2992	34.8167	Tropical and Subtropical Moist Broadleaf Forests	East African montane forests	Photograph	All	no data	Phillip Henschel
Non-melanistic 244	*pardus*	Masai Mara National Park, Kenya	-1.6613	35.3135	Tropical and Subtropical Grasslands, Savannas and Shrublands	East African Acacia Savannas	Photograph	All	no data	Phillip Henschel
Non-melanistic 245	*pardus*	Masai Mara National Park, Kenya	-1.4010	34.8578	Tropical and Subtropical Grasslands, Savannas and Shrublands	East African Acacia Savannas	Photograph	All	no data	Phillip Henschel
Non-melanistic 246	*pardus*	Mount Kenya's, Kenya	-0.3075	37.5967	Tropical and Subtropical Grasslands, Savannas and Shrublands	East African Acacia Savannas	Photograph	All	no data	Smithsonian Institution
Non-melanistic 247	*pardus*	Mount Kenya's, Kenya	0.0003	37.6178	Tropical and Subtropical Moist Broadleaf Forests	East African montane forests	Photograph	All	no data	Smithsonian Institution
Non-melanistic 248	*pardus*	Ol Pejeta Kenya’s Laikipia District, Kenya	0.1262	36.8511	Tropical and Subtropical Grasslands, Savannas and Shrublands	East African Acacia Savannas	Photograph	All	no data	Smithsonian Institution
Non-melanistic 249	*pardus*	South Samburu, Kenya	-3.6851	39.2473	Tropical and Subtropical Grasslands, Savannas and Shrublands	East African Acacia Savannas	Photograph	All	no data	Phillip Henschel
Non-melanistic 250	*pardus*	Samburu Natural Reserve, Kenya	1.0857	38.2465	Deserts and Xeric Shrublands	Masai xeric grasslands and shrublands	Photograph	All	no data	Andrew Stein
Non-melanistic 251	*pardus*	Selenkay Safari Camp, Kenya	-0.4115	36.0993	Tropical and Subtropical Grasslands, Savannas and Shrublands	East African Acacia Savannas	Photograph	All	no data	Selenkay Safari Camp
Non-melanistic 252	*pardus*	Shaba National Reserve, Kenya	0.5476	37.2778	Tropical and Subtropical Grasslands, Savannas and Shrublands	East African Acacia Savannas	Photograph	All	no data	Andrew Stein
Non-melanistic 253	*pardus*	Tumaren Ranch, Kenya	0.1099	36.7982	Tropical and Subtropical Grasslands, Savannas and Shrublands	East African Acacia Savannas	Photograph	All	2009	Tumaren Ranch
Non-melanistic 254	*pardus*	Tumaren Ranch, Kenya	0.1601	36.6890	Tropical and Subtropical Grasslands, Savannas and Shrublands	East African Acacia Savannas	Photograph	All	2000	Tumaren Ranch
Non-melanistic 255	*pardus*	Tumaren Ranch, Kenya	0.1252	36.8827	Tropical and Subtropical Grasslands, Savannas and Shrublands	East African Acacia Savannas	Photograph	All	2010	Tumaren Ranch
Non-melanistic 256	*pardus*	Tumaren Ranch, Kenya	0.1113	36.8903	Tropical and Subtropical Grasslands, Savannas and Shrublands	East African Acacia Savannas	Photograph	All	2009	Tumaren Ranch
Non-melanistic 257	*pardus*	Tumaren Ranch, Kenya	-0.0001	36.8788	Tropical and Subtropical Grasslands, Savannas and Shrublands	East African Acacia Savannas	Photograph	All	2008	Tumaren Ranch
Non-melanistic 258	*pardus*	Voi, Coast Province, Kenya	-3.3767	38.5138	Tropical and Subtropical Grasslands, Savannas and Shrublands	East African Acacia Savannas	Photograph	All	1911	National Museum of Natural History USA
Non-melanistic 259	*delacouri*	Nam Et-Phou Louey, Laos	20.6599	103.2908	Tropical and Subtropical Moist Broadleaf Forests	North Indochina subtropical moist forests	Photograph	All	2009	Bruce Kekule
Non-melanistic 260	*delacouri*	Plateau Bolovens, Laos	15.3908	106.3461	Tropical and Subtropical Moist Broadleaf Forests	Annamite Range moist forests	Photograph	All	1932	American Museum of Natural History USA
Non-melanistic 261	*pardus*	Monrovia, Liberia	6.3345	-10.6443	Tropical and Subtropical Moist Broadleaf Forests	Guinean Moist Forests	Photograph	All	no data	American Museum of Natural History USA
Non-melanistic 262	*pardus*	Lifupa Game Camp, Malawi	-13.0872	33.1512	Tropical and Subtropical Grasslands, Savannas and Shrublands	Southern Miombo woodlands	Photograph	All	no data	Smithsonian Institution
Non-melanistic 263	*pardus*	Lifupa Game Camp, Malawi	-13.1053	33.1605	Tropical and Subtropical Grasslands, Savannas and Shrublands	Southern Miombo woodlands	Photograph	All	no data	Smithsonian Institution
Non-melanistic 264	*pardus*	Mbobo, Malawi	-13.0286	33.9612	Tropical and Subtropical Grasslands, Savannas and Shrublands	Central Zambezian Miombo woodlands	Photograph	All	1946	American Museum of Natural History USA
Non-melanistic 265	*delacouri*	Endau-Rompin National Park, Malaysia	6.3561	101.3821	Tropical and Subtropical Moist Broadleaf Forests	Peninsular Malaysian rain forests	Photograph	All	2003	Asian Leopard Project
Non-melanistic 266	*delacouri*	Endau-Rompin National Park, Malaysia	6.0600	101.5340	Tropical and Subtropical Moist Broadleaf Forests	Peninsular Malaysian rain forests	Photograph	All	2010	Bruce Kekule
Non-melanistic 267	*delacouri*	Salween River, Myanmar	20.0657	98.4779	Tropical and Subtropical Moist Broadleaf Forests	Kayah-Karen/Tenasserim moist forests	Photograph	All	1899	Bertha Ferrars
Non-melanistic 268	*pardus*	Chiputo, Mocambique	-14.8612	32.2915	Tropical and Subtropical Grasslands, Savannas and Shrublands	Southern Miombo woodlands	Photograph	All	no data	American Museum of Natural History USA
Non-melanistic 269	*pardus*	Lake Malawi, Mocambique	-12.5962	34.9996	Tropical and Subtropical Grasslands, Savannas and Shrublands	Eastern Miombo woodlands	Photograph	All	no data	National Museum of Natural History USA
Non-melanistic 270	*pardus*	Niassa, Mocambique	-12.1415	36.1377	Tropical and Subtropical Grasslands, Savannas and Shrublands	Eastern Miombo woodlands	Photograph	All	2013	Niassa Lion Project
Non-melanistic 271	*pardus*	Chasie, Karakuwisa, Namibia	-19.1400	20.1400	Tropical and Subtropical Grasslands, Savannas and Shrublands	Kalahari Acacia-Baikiaea woodlands	Photograph	All	1952	National Museum of Natural History USA
Non-melanistic 272	*pardus*	Chasie, Karakuwisa, Namibia	-19.1400	20.1400	Tropical and Subtropical Grasslands, Savannas and Shrublands	Kalahari Acacia-Baikiaea woodlands	Photograph	All	1952	National Museum of Natural History USA
Non-melanistic 273	*pardus*	Epukiro, Namibia	-21.6196	20.0000	Deserts and Xeric Shrublands	Kalahari xeric savanna	Photograph	All	2012	Andrew Stein
Non-melanistic 274	*pardus*	Erindi Game Reserve, Namibia	-21.4790	16.4574	Deserts and Xeric Shrublands	Kalahari xeric savanna	Photograph	All	no data	Andrew Stein
Non-melanistic 275	*pardus*	Erindi Game Reserve, Namibia	-21.6078	16.4067	Deserts and Xeric Shrublands	Kalahari xeric savanna	Photograph	All	2010	Andrew Stein
Non-melanistic 276	*pardus*	Ettien Reserve, Namibia	-20.7896	19.9967	Tropical and Subtropical Grasslands, Savannas and Shrublands	Kalahari Acacia-Baikiaea woodlands	Photograph	All	no data	Andrew Stein
Non-melanistic 277	*pardus*	Ku Game Ranch, Namibia	-19.9140	16.1918	Tropical and Subtropical Grasslands, Savannas and Shrublands	Angolan Mopane woodlands	Photograph	All	no data	Ezekiel Fabiano Chimbioputo
Non-melanistic 278	*pardus*	Naukluft Mountains Park, Namibia	-24.1521	16.2544	Deserts and Xeric Shrublands	Namibian savanna woodlands	Photograph	All	2013	Duke University
Non-melanistic 279	*pardus*	Naukluft Mountains Park, Namibia	-24.1954	16.1514	Deserts and Xeric Shrublands	Namibian savanna woodlands	Photograph	All	2013	Duke University
Non-melanistic 280	*pardus*	Okaputa, Namibia	-20.0934	17.5168	Tropical and Subtropical Grasslands, Savannas and Shrublands	Kalahari Acacia-Baikiaea woodlands	Photograph	All	no data	Andrew Stein
Non-melanistic 281	*pardus*	Okaputa, Namibia	-20.0934	17.5168	Tropical and Subtropical Grasslands, Savannas and Shrublands	Kalahari Acacia-Baikiaea woodlands	Photograph	All	no data	Andrew Stein
Non-melanistic 282	*pardus*	Okaputa, Namibia	-20.0934	17.5168	Tropical and Subtropical Grasslands, Savannas and Shrublands	Kalahari Acacia-Baikiaea woodlands	Photograph	All	no data	Andrew Stein
Non-melanistic 283	*pardus*	Okaputa, Namibia	-20.0934	17.5168	Tropical and Subtropical Grasslands, Savannas and Shrublands	Kalahari Acacia-Baikiaea woodlands	Photograph	All	no data	Andrew Stein
Non-melanistic 284	*pardus*	Otjinene, Namibia	-20.8011	20.1926	Tropical and Subtropical Grasslands, Savannas and Shrublands	Kalahari Acacia-Baikiaea woodlands	Photograph	All	2012	Andrew Stein
Non-melanistic 285	*pardus*	Otjiwarongo, Namibia	-20.5101	16.7498	Deserts and Xeric Shrublands	Kalahari xeric savanna	Photograph	All	2009	Ezekiel Fabiano Chimbioputo
Non-melanistic 286	*pardus*	Otjiwarongo, Namibia	-20.4627	17.1710	Deserts and Xeric Shrublands	Kalahari xeric savanna	Photograph	All	2010	Ezekiel Fabiano Chimbioputo
Non-melanistic 287	*pardus*	Otjiwarongo, Namibia	-20.4630	17.1714	Deserts and Xeric Shrublands	Kalahari xeric savanna	Photograph	All	2010	Ezekiel Fabiano Chimbioputo
Non-melanistic 288	*pardus*	Otjiwarongo, Namibia	-20.4364	17.1139	Deserts and Xeric Shrublands	Kalahari xeric savanna	Photograph	All	2010	Ezekiel Fabiano Chimbioputo
Non-melanistic 289	*pardus*	Otjiwarongo, Namibia	-20.3995	17.0962	Deserts and Xeric Shrublands	Kalahari xeric savanna	Photograph	All	2010	Ezekiel Fabiano Chimbioputo
Non-melanistic 290	*pardus*	Otjiwarongo, Namibia	-20.4582	17.0907	Deserts and Xeric Shrublands	Kalahari xeric savanna	Photograph	All	2010	Ezekiel Fabiano Chimbioputo
Non-melanistic 291	*pardus*	Otjiwarongo, Namibia	-20.3890	17.0827	Deserts and Xeric Shrublands	Kalahari xeric savanna	Photograph	All	2010	Ezekiel Fabiano Chimbioputo
Non-melanistic 292	*pardus*	Otjiwarongo, Namibia	-20.4587	17.1401	Deserts and Xeric Shrublands	Kalahari xeric savanna	Photograph	All	2010	Ezekiel Fabiano Chimbioputo
Non-melanistic 293	*pardus*	Otjiwarongo, Namibia	-20.4674	16.9741	Deserts and Xeric Shrublands	Kalahari xeric savanna	Photograph	All	2010	Ezekiel Fabiano Chimbioputo
Non-melanistic 294	*pardus*	Otjiwarongo, Namibia	-20.4788	17.1425	Deserts and Xeric Shrublands	Kalahari xeric savanna	Photograph	All	2011	Ezekiel Fabiano Chimbioputo
Non-melanistic 295	*pardus*	Otjiwarongo, Namibia	-20.4331	17.0815	Deserts and Xeric Shrublands	Kalahari xeric savanna	Photograph	All	2011	Ezekiel Fabiano Chimbioputo

Id	Subspecies	Location	Deg. - WGS84		Biome	Ecorregion	Sample	Analysis	Year	Source
			Latitude	Longitude						
Non-melanistic 296	*pardus*	Reitfonten, Namibia	-20.8792	20.8462	Tropical and Subtropical Grasslands, Savannas and Shrublands	Kalahari Acacia-Baikiaea woodlands	Photograph	All	2011	Smithsonian Institution
Non-melanistic 297	*pardus*	Reitfonten, Namibia	-21.4373	20.6209	Tropical and Subtropical Grasslands, Savannas and Shrublands	Kalahari Acacia-Baikiaea woodlands	Photograph	All	2011	Smithsonian Institution
Non-melanistic 298	*pardus*	Tsumkwe, Namibia	-19.7844	20.5430	Tropical and Subtropical Grasslands, Savannas and Shrublands	Kalahari Acacia-Baikiaea woodlands	Photograph	All	2011	Smithsonian Institution
Non-melanistic 299	*pardus*	Waterberg, Namibia	-20.4613	17.2081	Deserts and Xeric Shrublands	Kalahari xeric savanna	Photograph	All	no data	Andrew Stein
Non-melanistic 300	*pardus*	Waterberg, Namibia	-20.4613	17.2081	Deserts and Xeric Shrublands	Kalahari xeric savanna	Photograph	All	no data	Andrew Stein
Non-melanistic 301	*pardus*	Waterberg, Namibia	-20.4613	17.2081	Deserts and Xeric Shrublands	Kalahari xeric savanna	Photograph	All	no data	Andrew Stein
Non-melanistic 302	*pardus*	Waterberg, Namibia	-20.4613	17.2081	Deserts and Xeric Shrublands	Kalahari xeric savanna	Photograph	All	no data	Andrew Stein
Non-melanistic 303	*pardus*	Waterberg, Namibia	-20.4613	17.2081	Deserts and Xeric Shrublands	Kalahari xeric savanna	Photograph	All	no data	Andrew Stein
Non-melanistic 304	*fusca*	Bardia National Park, Nepal	28.7941	81.1997	Tropical and Subtropical Moist Broadleaf Forests	Himalayan subtropical broadleaf forests	Photograph	All	no data	Smithsonian Institution
Non-melanistic 305	*fusca*	Chitwan National Park, Nepal	27.4529	84.4404	Tropical and Subtropical Moist Broadleaf Forests	Himalayan subtropical broadleaf forests	Photograph	All	no data	Michigan State University
Non-melanistic 306	*fusca*	Chitwan National Park, Nepal	27.4979	84.0197	Tropical and Subtropical Moist Broadleaf Forests	Himalayan subtropical broadleaf forests	Photograph	All	2010	Michigan State University
Non-melanistic 307	*fusca*	Ghansa, Nepal	28.7834	83.7560	Temperate Broadleaf and Mixed Forests	Western Himalayan broadleaf forests	Photograph	All	no data	Ghimirey 2006
Non-melanistic 308	*pardus*	Donga, Nigeria	7.6133	10.0462	Tropical and Subtropical Grasslands, Savannas and Shrublands	Guinean forest-savanna mosaic	Photograph	All	no data	National Geographic Society
Non-melanistic 309	*pardus*	Niger Delta, Nigeria	5.1980	6.3856	Tropical and Subtropical Moist Broadleaf Forests	Niger Delta swamp forests	Photograph	All	no data	Ikemeh 2007
Non-melanistic 310	*pardus*	Otuan, Nigeria	4.8771	6.0880	Tropical and Subtropical Moist Broadleaf Forests	Niger Delta swamp forests	Photograph	All	1961	National Museum of Natural History USA
Non-melanistic 311	*nimr*	Dofar Mountains, Oman	17.2705	53.6453	Deserts and Xeric Shrublands	Red Sea Nubo-Sindian tropical desert and semi-desert	Photograph	All	no data	British Exploring Society
Non-melanistic 312	*nimr*	Jabal Samhan Nature Reserve, Oman	17.2712	54.8702	Deserts and Xeric Shrublands	Arabian Highlands woodlands and shrublands	Photograph	All	no data	Jane Budd
Non-melanistic 313	*nimr*	Jabal Samhan Nature Reserve, Oman	17.2246	55.1100	Deserts and Xeric Shrublands	Arabian Highlands woodlands and shrublands	Photograph	All	no data	Spalton et al 2006
Non-melanistic 314	*nimr*	Samhan Nature Reserve, Oman	17.0013	54.8161	Deserts and Xeric Shrublands	Arabian Highlands woodlands and shrublands	Photograph	All	no data	Jane Budd
Non-melanistic 315	*nimr*	Samhan Nature Reserve, Oman	17.0792	54.8472	Deserts and Xeric Shrublands	Arabian Highlands woodlands and shrublands	Photograph	All	no data	Jane Budd
Non-melanistic 316	*fusca*	Machiara National Park, Pakistan	35.6015	74.2158	Temperate Coniferous Forest	Western Himalayan temperate forests	Photograph	All	2012	World Wild Fund
Non-melanistic 317	*fusca*	Pir Lasora National Park, Pakistan	33.4133	74.0126	Tropical and Subtropical Coniferous Forests	Himalayan subtropical pine forests	Photograph	All	no data	World Wild Fund
Non-melanistic 318	*fusca*	Pir Lasora National Park, Pakistan	33.3329	74.0563	Tropical and Subtropical Coniferous Forests	Himalayan subtropical pine forests	Photograph	All	no data	World Wild Fund
Non-melanistic 319	*orientalis*	Ayandeki, Russia	43.4387	131.3109	Temperate Broadleaf and Mixed Forests	Manchurian mixed forests	Photograph	All	no data	World Wild Fund
Non-melanistic 320	*orientalis*	Kedrovaya Pad Reserve, Russia	43.8189	131.5116	Temperate Broadleaf and Mixed Forests	Manchurian mixed forests	Photograph	All	no data	Yury Shibnev
Non-melanistic 321	*orientalis*	Kedrovaya Pad Reserve, Russia	43.5906	131.5544	Temperate Broadleaf and Mixed Forests	Manchurian mixed forests	Photograph	All	no data	Yury Shibnev
Non-melanistic 322	*orientalis*	Primorskiy Krai, Nezhino Hunting Lease, Russia	43.7012	131.7596	Temperate Broadleaf and Mixed Forests	Manchurian mixed forests	Photograph	All	no data	Ekaterina Nicolaeva/Dale Miquelle
Non-melanistic 323	*orientalis*	Primorskiy Krai, Nezhino Hunting Lease, Russia	43.7012	131.7596	Temperate Broadleaf and Mixed Forests	Manchurian mixed forests	Photograph	All	no data	Ekaterina Nicolaeva/Dale Miquelle
Non-melanistic 324	*orientalis*	Primorskiy Krai, Nezhino Hunting Lease, Russia	43.7012	131.7596	Temperate Broadleaf and Mixed Forests	Manchurian mixed forests	Photograph	All	no data	Ekaterina Nicolaeva/Dale Miquelle
Non-melanistic 325	*orientalis*	Primorskiy Krai, Nezhino Hunting Lease, Russia	43.7012	131.7596	Temperate Broadleaf and Mixed Forests	Manchurian mixed forests	Photograph	All	no data	Ekaterina Nicolaeva/Dale Miquelle
Non-melanistic 326	*orientalis*	Primorskiy Krai, Nezhino Hunting Lease, Russia	43.3135	131.2976	Temperate Broadleaf and Mixed Forests	Manchurian mixed forests	Photograph	All	no data	Ekaterina Nicolaeva/Dale Miquelle
Non-melanistic 327	*orientalis*	Primorskiy Krai, Nezhino Hunting Lease, Russia	43.3135	131.2976	Temperate Broadleaf and Mixed Forests	Manchurian mixed forests	Photograph	All	no data	Ekaterina Nicolaeva/Dale Miquelle
Non-melanistic 328	*orientalis*	Primorskiy Krai, Nezhino Hunting Lease, Russia	43.3135	131.2976	Temperate Broadleaf and Mixed Forests	Manchurian mixed forests	Photograph	All	no data	Ekaterina Nicolaeva/Dale Miquelle
Non-melanistic 329	*orientalis*	Primorskiy Krai, Nezhino Hunting Lease, Russia	43.3135	131.2976	Temperate Broadleaf and Mixed Forests	Manchurian mixed forests	Photograph	All	no data	Ekaterina Nicolaeva/Dale Miquelle
Non-melanistic 330	*orientalis*	Primorskiy Krai, Nezhino Hunting Lease, Russia	43.3135	131.2976	Temperate Broadleaf and Mixed Forests	Manchurian mixed forests	Photograph	All	no data	Ekaterina Nicolaeva/Dale Miquelle
Non-melanistic 331	*orientalis*	Primorskiy Krai, Nezhino Hunting Lease, Russia	43.0242	131.1564	Temperate Broadleaf and Mixed Forests	Manchurian mixed forests	Photograph	All	no data	Ekaterina Nicolaeva/Dale Miquelle
Non-melanistic 332	*orientalis*	Primorskiy Krai, Nezhino Hunting Lease, Russia	43.0242	131.1564	Temperate Broadleaf and Mixed Forests	Manchurian mixed forests	Photograph	All	no data	Ekaterina Nicolaeva/Dale Miquelle
Non-melanistic 333	*orientalis*	Primorskiy Krai, Nezhino Hunting Lease, Russia	43.0242	131.1564	Temperate Broadleaf and Mixed Forests	Manchurian mixed forests	Photograph	All	no data	Ekaterina Nicolaeva/Dale Miquelle
Non-melanistic 334	*orientalis*	Primorskiy Krai, Nezhino Hunting Lease, Russia	43.0242	131.1564	Temperate Broadleaf and Mixed Forests	Manchurian mixed forests	Photograph	All	no data	Ekaterina Nicolaeva/Dale Miquelle
Non-melanistic 335	*orientalis*	Primorskiy Krai, Nezhino Hunting Lease, Russia	43.0242	131.1564	Temperate Broadleaf and Mixed Forests	Manchurian mixed forests	Photograph	All	no data	Ekaterina Nicolaeva/Dale Miquelle
Non-melanistic 336	*orientalis*	Primorskiy Krai, Nezhino Hunting Lease, Russia	42.8721	131.1602	Temperate Broadleaf and Mixed Forests	Manchurian mixed forests	Photograph	All	no data	Ekaterina Nicolaeva/Dale Miquelle
Non-melanistic 337	*orientalis*	Primorskiy Krai, Nezhino Hunting Lease, Russia	42.8721	131.1602	Temperate Broadleaf and Mixed Forests	Manchurian mixed forests	Photograph	All	no data	Ekaterina Nicolaeva/Dale Miquelle
Non-melanistic 338	*orientalis*	Primorskiy Krai, Nezhino Hunting Lease, Russia	42.8721	131.1602	Temperate Broadleaf and Mixed Forests	Manchurian mixed forests	Photograph	All	no data	Ekaterina Nicolaeva/Dale Miquelle
Non-melanistic 339	*orientalis*	Primorskiy Krai, Nezhino Hunting Lease, Russia	42.8721	131.1602	Temperate Broadleaf and Mixed Forests	Manchurian mixed forests	Photograph	All	no data	Ekaterina Nicolaeva/Dale Miquelle
Non-melanistic 340	*orientalis*	Primorskiy Krai, Nezhino Hunting Lease, Russia	42.8721	131.1602	Temperate Broadleaf and Mixed Forests	Manchurian mixed forests	Photograph	All	no data	Ekaterina Nicolaeva/Dale Miquelle
Non-melanistic 341	*orientalis*	Primorskiy Krai, Nezhino Hunting Lease, Russia	42.8721	131.1602	Temperate Broadleaf and Mixed Forests	Manchurian mixed forests	Photograph	All	no data	Ekaterina Nicolaeva/Dale Miquelle
Non-melanistic 342	*orientalis*	Primorskiy Krai, Nezhino Hunting Lease, Russia	42.8721	131.1602	Temperate Broadleaf and Mixed Forests	Manchurian mixed forests	Photograph	All	no data	Ekaterina Nicolaeva/Dale Miquelle
Non-melanistic 343	*orientalis*	Primorskiy Krai, Nezhino Hunting Lease, Russia	42.8094	130.7478	Temperate Broadleaf and Mixed Forests	Manchurian mixed forests	Photograph	All	no data	Ekaterina Nicolaeva/Dale Miquelle
Non-melanistic 344	*orientalis*	Primorskiy Krai, Nezhino Hunting Lease, Russia	42.8094	130.7478	Temperate Broadleaf and Mixed Forests	Manchurian mixed forests	Photograph	All	no data	Ekaterina Nicolaeva/Dale Miquelle
Non-melanistic 345	*orientalis*	Primorskiy Krai, Nezhino Hunting Lease, Russia	42.8094	130.7478	Temperate Broadleaf and Mixed Forests	Manchurian mixed forests	Photograph	All	no data	Ekaterina Nicolaeva/Dale Miquelle
Non-melanistic 346	*orientalis*	Primorskiy Krai, Nezhino Hunting Lease, Russia	42.8094	130.7478	Temperate Broadleaf and Mixed Forests	Manchurian mixed forests	Photograph	All	no data	Ekaterina Nicolaeva/Dale Miquelle
Non-melanistic 347	*orientalis*	Southwest Primorye, Russia	43.5209	131.7363	Temperate Broadleaf and Mixed Forests	Manchurian mixed forests	Photograph	All	2004	Wildlife Conservation Society - Alexander Reebin
Non-melanistic 348	*orientalis*	Southwest Primorye, Russia	43.4308	131.5017	Temperate Broadleaf and Mixed Forests	Manchurian mixed forests	Photograph	All	2007	Wildlife Conservation Society - Alexander Reebin
Non-melanistic 349	*orientalis*	Southwest Primorye, Russia	43.4968	131.6842	Temperate Broadleaf and Mixed Forests	Manchurian mixed forests	Photograph	All	2003	Wildlife Conservation Society - Alexander Reebin
Non-melanistic 350	*orientalis*	Southwest Primorye, Russia	43.4772	131.6761	Temperate Broadleaf and Mixed Forests	Manchurian mixed forests	Photograph	All	no data	Wildlife Conservation Society - Alexander Reebin
Non-melanistic 351	*orientalis*	Southwest Primorye, Russia	43.4405	131.4546	Temperate Broadleaf and Mixed Forests	Manchurian mixed forests	Photograph	All	2004	Wildlife Conservation Society - Alexander Reebin
Non-melanistic 352	*orientalis*	Southwest Primorye, Russia	43.3997	131.6481	Temperate Broadleaf and Mixed Forests	Manchurian mixed forests	Photograph	All	2004	Wildlife Conservation Society - Alexander Reebin
Non-melanistic 353	*orientalis*	Southwest Primorye, Russia	43.3997	131.6481	Temperate Broadleaf and Mixed Forests	Manchurian mixed forests	Photograph	All	2004	Wildlife Conservation Society - Alexander Reebin
Non-melanistic 354	*orientalis*	Southwest Primorye, Russia	43.3997	131.6481	Temperate Broadleaf and Mixed Forests	Manchurian mixed forests	Photograph	All	2004	Wildlife Conservation Society - Alexander Reebin
Non-melanistic 355	*orientalis*	Southwest Primorye, Russia	43.3997	131.6481	Temperate Broadleaf and Mixed Forests	Manchurian mixed forests	Photograph	All	2004	Wildlife Conservation Society - Alexander Reebin

Id	Subspecies	Location	Deg. - WGS84		Biome	Ecoregion	Sample	Analysis	Year	Source
			Latitude	Longitude						
Non-melanistic 356	*orientalis*	Southwest Primorye, Russia	43.5018	131.5278	Temperate Broadleaf and Mixed Forests	Manchurian mixed forests	Photograph	All	2004	Wildlife Conservation Society - Alexander Reebin
Non-melanistic 357	*orientalis*	Southwest Primorye, Russia	43.4375	131.7086	Temperate Broadleaf and Mixed Forests	Manchurian mixed forests	Photograph	All	2001	Wildlife Conservation Society - Alexander Reebin
Non-melanistic 358	*orientalis*	Southwest Primorye, Russia	43.4375	131.7086	Temperate Broadleaf and Mixed Forests	Manchurian mixed forests	Photograph	All	2004	Wildlife Conservation Society - Alexander Reebin
Non-melanistic 359	*orientalis*	Southwest Primorye, Russia	43.4765	131.6034	Temperate Broadleaf and Mixed Forests	Manchurian mixed forests	Photograph	All	2004	Wildlife Conservation Society - Alexander Reebin
Non-melanistic 360	*orientalis*	Southwest Primorye, Russia	43.4765	131.6034	Temperate Broadleaf and Mixed Forests	Manchurian mixed forests	Photograph	All	2007	Wildlife Conservation Society - Alexander Reebin
Non-melanistic 361	*orientalis*	Southwest Primorye, Russia	43.5164	131.6544	Temperate Broadleaf and Mixed Forests	Manchurian mixed forests	Photograph	All	2005	Wildlife Conservation Society - Alexander Reebin
Non-melanistic 362	*orientalis*	Southwest Primorye, Russia	43.4693	131.5529	Temperate Broadleaf and Mixed Forests	Manchurian mixed forests	Photograph	All	2005	Wildlife Conservation Society - Alexander Reebin
Non-melanistic 363	*orientalis*	Southwest Primorye, Russia	43.4821	131.5401	Temperate Broadleaf and Mixed Forests	Manchurian mixed forests	Photograph	All	2005	Wildlife Conservation Society - Alexander Reebin
Non-melanistic 364	*orientalis*	Southwest Primorye, Russia	43.4511	131.5435	Temperate Broadleaf and Mixed Forests	Manchurian mixed forests	Photograph	All	2011	Wildlife Conservation Society - Alexander Reebin
Non-melanistic 365	*orientalis*	Southwest Primorye, Russia	43.4189	131.5842	Temperate Broadleaf and Mixed Forests	Manchurian mixed forests	Photograph	All	2007	Wildlife Conservation Society - Alexander Reebin
Non-melanistic 366	*orientalis*	Southwest Primorye, Russia	43.4096	131.4165	Temperate Broadleaf and Mixed Forests	Manchurian mixed forests	Photograph	All	2011	Wildlife Conservation Society - Alexander Reebin
Non-melanistic 367	*orientalis*	Southwest Primorye, Russia	43.4096	131.4165	Temperate Broadleaf and Mixed Forests	Manchurian mixed forests	Photograph	All	2007	Wildlife Conservation Society - Alexander Reebin
Non-melanistic 368	*orientalis*	Southwest Primorye, Russia	43.4096	131.4165	Temperate Broadleaf and Mixed Forests	Manchurian mixed forests	Photograph	All	2011	Wildlife Conservation Society - Alexander Reebin
Non-melanistic 369	*orientalis*	Southwest Primorye, Russia	43.3791	131.5503	Temperate Broadleaf and Mixed Forests	Manchurian mixed forests	Photograph	All	2007	Wildlife Conservation Society - Alexander Reebin
Non-melanistic 370	*orientalis*	Southwest Primorye, Russia	43.5060	131.6388	Temperate Broadleaf and Mixed Forests	Manchurian mixed forests	Photograph	All	2007	Wildlife Conservation Society - Alexander Reebin
Non-melanistic 371	*orientalis*	Southwest Primorye, Russia	43.5333	131.7144	Temperate Broadleaf and Mixed Forests	Manchurian mixed forests	Photograph	All	2007	Wildlife Conservation Society - Alexander Reebin
Non-melanistic 372	*orientalis*	Southwest Primorye, Russia	43.5333	131.7144	Temperate Broadleaf and Mixed Forests	Manchurian mixed forests	Photograph	All	2007	Wildlife Conservation Society - Alexander Reebin
Non-melanistic 373	*orientalis*	Southwest Primorye, Russia	43.5333	131.7144	Temperate Broadleaf and Mixed Forests	Manchurian mixed forests	Photograph	All	2008	Wildlife Conservation Society - Alexander Reebin
Non-melanistic 374	*orientalis*	Southwest Primorye, Russia	43.5333	131.7144	Temperate Broadleaf and Mixed Forests	Manchurian mixed forests	Photograph	All	2008	Wildlife Conservation Society - Alexander Reebin
Non-melanistic 375	*orientalis*	Southwest Primorye, Russia	43.3238	131.5431	Temperate Broadleaf and Mixed Forests	Manchurian mixed forests	Photograph	All	2008	Wildlife Conservation Society - Alexander Reebin
Non-melanistic 376	*orientalis*	Southwest Primorye, Russia	43.3506	131.4963	Temperate Broadleaf and Mixed Forests	Manchurian mixed forests	Photograph	All	2009	Wildlife Conservation Society - Alexander Reebin
Non-melanistic 377	*orientalis*	Southwest Primorye, Russia	43.3506	131.4963	Temperate Broadleaf and Mixed Forests	Manchurian mixed forests	Photograph	All	2011	Wildlife Conservation Society - Alexander Reebin
Non-melanistic 378	*orientalis*	Southwest Primorye, Russia	43.3506	131.4963	Temperate Broadleaf and Mixed Forests	Manchurian mixed forests	Photograph	All	2010	Wildlife Conservation Society - Alexander Reebin
Non-melanistic 379	*orientalis*	Southwest Primorye, Russia	43.3506	131.4963	Temperate Broadleaf and Mixed Forests	Manchurian mixed forests	Photograph	All	2010	Wildlife Conservation Society - Alexander Reebin
Non-melanistic 380	*orientalis*	Southwest Primorye, Russia	43.3506	131.4963	Temperate Broadleaf and Mixed Forests	Manchurian mixed forests	Photograph	All	2010	Wildlife Conservation Society - Alexander Reebin
Non-melanistic 381	*orientalis*	Southwest Primorye, Russia	43.3506	131.4963	Temperate Broadleaf and Mixed Forests	Manchurian mixed forests	Photograph	All	2011	Wildlife Conservation Society - Alexander Reebin
Non-melanistic 382	*orientalis*	Southwest Primorye, Russia	43.3506	131.4963	Temperate Broadleaf and Mixed Forests	Manchurian mixed forests	Photograph	All	2010	Wildlife Conservation Society - Alexander Reebin
Non-melanistic 383	*orientalis*	Southwest Primorye, Russia	43.3506	131.4963	Temperate Broadleaf and Mixed Forests	Manchurian mixed forests	Photograph	All	2011	Wildlife Conservation Society - Alexander Reebin
Non-melanistic 384	*orientalis*	Southwest Primorye, Russia	43.3506	131.4963	Temperate Broadleaf and Mixed Forests	Manchurian mixed forests	Photograph	All	2011	Wildlife Conservation Society - Alexander Reebin
Non-melanistic 385	*orientalis*	Southwest Primorye, Russia	43.3506	131.4963	Temperate Broadleaf and Mixed Forests	Manchurian mixed forests	Photograph	All	2011	Wildlife Conservation Society - Alexander Reebin
Non-melanistic 386	*orientalis*	Southwest Primorye, Russia	43.4439	131.5999	Temperate Broadleaf and Mixed Forests	Manchurian mixed forests	Photograph	All	2011	Wildlife Conservation Society - Alexander Reebin
Non-melanistic 387	*orientalis*	Southwest Primorye, Russia	43.4439	131.5999	Temperate Broadleaf and Mixed Forests	Manchurian mixed forests	Photograph	All	2011	Wildlife Conservation Society - Alexander Reebin
Non-melanistic 388	*orientalis*	Ussuri, Nicolsk, Russia	43.8230	132.0324	Tundra	Suiphun-Khanka meadows and forest meadows	Photograph	All	1930	American Museum of Natural History USA
Non-melanistic 389	*orientalis*	Ussuri, Okransk, Russia	43.8283	131.8699	Tundra	Suiphun-Khanka meadows and forest meadows	Photograph	All	1930	American Museum of Natural History USA
Non-melanistic 390	*pardus*	Kigali, Rwanda	-1.9764	30.0001	Tropical and Subtropical Grasslands, Savannas and Shrublands	Victoria Basin forest-savanna mosaic	Photograph	All	no data	Smithsonian Institution
Non-melanistic 391	*pardus*	Agulhas Plain Great Hermanus Area, South Africa	-34.6572	19.7481	Mediterranean Forests, Woodlands, and Scrub	Lowland fynbos and renosterveld	Photograph	All	2012	Landmark Foundation
Non-melanistic 392	*pardus*	Agulhas Plain Great Hermanus Area, South Africa	-34.6418	19.7119	Mediterranean Forests, Woodlands, and Scrub	Lowland fynbos and renosterveld	Photograph	All	2012	Landmark Foundation
Non-melanistic 393	*pardus*	Cederberg Mountains, Western Cape, South Africa	-32.2916	19.4120	Deserts and Xeric Shrublands	Namib-Karoo-Kaokoveld Deserts and Shrublands	Photograph	All	2010	Guy Balme
Non-melanistic 394	*pardus*	Cederberg Mountains, Western Cape, South Africa	-32.3383	19.1853	Mediterranean Forests, Woodlands, and Scrub	Montane fynbos and renosterveld	Photograph	All	no data	Guy Balme
Non-melanistic 395	*pardus*	Franschhoek La Motte Hiking Trail, South Africa	-33.9428	19.1925	Mediterranean Forests, Woodlands, and Scrub	Montane fynbos and renosterveld	Photograph	All	2011	Cape Leopard Trust
Non-melanistic 396	*pardus*	Franschhoek La Motte Hiking Trail, South Africa	-33.9687	19.2022	Mediterranean Forests, Woodlands, and Scrub	Montane fynbos and renosterveld	Photograph	All	2011	Cape Leopard Trust
Non-melanistic 397	*pardus*	Hoedspruit Game Reserve, South Africa	-24.1221	31.1049	Tropical and Subtropical Grasslands, Savannas and Shrublands	Zambezian and Mopane woodlands	Photograph	All	no data	Guy Balme
Non-melanistic 398	*pardus*	Karatara, South Africa	-33.9502	22.3380	Mediterranean Forests, Woodlands, and Scrub	Fynbos	Photograph	All	2013	Braczkowski & Watson 2013
Non-melanistic 399	*pardus*	Karatara, South Africa	-33.8570	22.2120	Mediterranean Forests, Woodlands, and Scrub	Fynbos	Photograph	All	2013	Braczkowski & Watson 2013
Non-melanistic 400	*pardus*	Karongwe Reserve, South Africa	-24.5092	30.5079	Montane Grasslands and Shrublands	Drakensberg Montane Woodlands and Grasslands	Photograph	All	no data	Karongwe Reserve Group
Non-melanistic 401	*pardus*	Kruger National Park, South Africa	-24.5210	31.2337	Tropical and Subtropical Grasslands, Savannas and Shrublands	Zambezian and Mopane woodlands	Photograph	All	2013	Eduardo Eizirik
Non-melanistic 402	*pardus*	Kruger National Park, South Africa	-23.6331	30.6962	Tropical and Subtropical Grasslands, Savannas and Shrublands	Zambezian and Mopane woodlands	Photograph	All	2012	Daniel Rocha
Non-melanistic 403	*pardus*	Kruger National Park, South Africa	-22.8847	31.3549	Tropical and Subtropical Grasslands, Savannas and Shrublands	Zambezian and Mopane woodlands	Photograph	All	no data	Guy Balme
Non-melanistic 404	*pardus*	Kudu Game Ranch, South Africa	-24.7933	30.5083	Montane Grasslands and Shrublands	Drakensberg Montane Woodlands and Grasslands	Photograph	All	no data	Conservation International
Non-melanistic 405	*pardus*	KwaZulu-Natal, South Africa	-28.4606	30.6808	Montane Grasslands and Shrublands	Drakensberg Montane Woodlands and Grasslands	Photograph	All	no data	Conservation International
Non-melanistic 406	*pardus*	KwaZulu-Natal, South Africa	-28.4766	30.7523	Montane Grasslands and Shrublands	Drakensberg Montane Woodlands and Grasslands	Photograph	All	no data	Conservation International
Non-melanistic 407	*pardus*	Leap Vineyards, South Africa	-33.9187	19.6263	Deserts and Xeric Shrublands	Namib-Karoo-Kaokoveld Deserts and Shrublands	Photograph	All	2013	Cape Leopard Trust
Non-melanistic 408	*pardus*	Leap Vineyards, South Africa	-33.9525	19.6478	Deserts and Xeric Shrublands	Namib-Karoo-Kaokoveld Deserts and Shrublands	Photograph	All	2010	Cape Leopard Trust
Non-melanistic 409	*pardus*	Letaba, South Africa	-23.6260	31.5994	Tropical and Subtropical Grasslands, Savannas and Shrublands	Zambezian and Mopane woodlands	Photograph	All	2010	Michelle Altenkirk
Non-melanistic 410	*pardus*	Limpopo, South Africa	-22.5536	30.9343	Tropical and Subtropical Grasslands, Savannas and Shrublands	Southern Africa bushveld	Photograph	All	2004	Conservation International
Non-melanistic 411	*pardus*	Limpopo Waterberg Area, South Africa	-22.1575	29.6163	Tropical and Subtropical Grasslands, Savannas and Shrublands	Zambezian and Mopane woodlands	Photograph	All	no data	Andrew Stein
Non-melanistic 412	*pardus*	Loskop Dam Protected Area, South Africa	-25.4369	29.2630	Tropical and Subtropical Grasslands, Savannas and Shrublands	Southern Africa bushveld	Photograph	All	2004	Guy Balme
Non-melanistic 413	*pardus*	Lydenburg, South Africa	-24.8234	30.8106	Montane Grasslands and Shrublands	Drakensberg Montane Woodlands and Grasslands	Photograph	All	no data	Guy Balme
Non-melanistic 414	*pardus*	Lydenburg, South Africa	-24.6917	30.7609	Montane Grasslands and Shrublands	Drakensberg Montane Woodlands and Grasslands	Photograph	All	2011	Guy Balme
Non-melanistic 415	*pardus*	Lydenburg, South Africa	-24.8012	30.7291	Montane Grasslands and Shrublands	Drakensberg Montane Woodlands and Grasslands	Photograph	All	2010	Sharon Hammond
Non-melanistic 416	*pardus*	Lydenburg, South Africa	-24.8079	30.6673	Montane Grasslands and Shrublands	Drakensberg Montane Woodlands and Grasslands	Photograph	All	2010	Sharon Hammond

Id	Subspecies	Location	Deg. - WGS84		Biome	Ecoregion	Sample	Analysis	Year	Source
			Latitude	Longitude						
Non-melanistic 417	*pardus*	Lydenburg, South Africa	-24.6807	30.8312	Montane Grasslands and Shrublands	Drakensberg Montane Woodlands and Grasslands	Photograph	All	2011	Sharon Hammond
Non-melanistic 418	*pardus*	Lydenburg Leopard Camp, South Africa	-24.9668	30.3398	Montane Grasslands and Shrublands	Drakensberg Montane Woodlands and Grasslands	Photograph	All	2011	Daniel Rocha
Non-melanistic 419	*pardus*	Lydenburg Leopard Camp, South Africa	-25.0000	30.7428	Montane Grasslands and Shrublands	Drakensberg Montane Woodlands and Grasslands	Photograph	All	no data	Daniel Rocha
Non-melanistic 420	*pardus*	Mkhuze Game Reserve, South Africa	-27.8100	32.0030	Montane Grasslands and Shrublands	Drakensberg Montane Woodlands and Grasslands	Photograph	All	no data	Guy Balme
Non-melanistic 421	*pardus*	Mkhuze Game Reserve, South Africa	-27.7886	31.8152	Montane Grasslands and Shrublands	Drakensberg Montane Woodlands and Grasslands	Photograph	All	2008	Smithsonian Institution
Non-melanistic 422	*pardus*	Mpumalanga, South Africa	-25.7211	31.6606	Tropical and Subtropical Grasslands, Savannas and Shrublands	Zambezian and Mopane woodlands	Photograph	All	2011	Michelle Altenkirk
Non-melanistic 423	*pardus*	Munyawana Leopard Project, South Africa	-27.7829	32.0600	Montane Grasslands and Shrublands	Drakensberg Montane Woodlands and Grasslands	Photograph	All	no data	Guy Balme
Non-melanistic 424	*pardus*	Phinda Private Game Reserve, South Africa	-27.8846	31.9637	Montane Grasslands and Shrublands	Drakensberg Montane Woodlands and Grasslands	Photograph	All	no data	Panthera
Non-melanistic 425	*pardus*	Riversdale, Western Cape, South Africa	-34.2139	21.1682	Mediterranean Forests, Woodlands, and Scrub	Fynbos	Photograph	All	2011	Landmark Foundation
Non-melanistic 426	*pardus*	Skukuza Road, South Africa	-24.9919	31.6263	Tropical and Subtropical Grasslands, Savannas and Shrublands	Zambezian and Mopane woodlands	Photograph	All	no data	Guy Balme
Non-melanistic 427	*pardus*	Somkhanda Game Reserve, South Africa	-27.7108	31.9462	Montane Grasslands and Shrublands	Drakensberg Montane Woodlands and Grasslands	Photograph	All	no data	Panthera
Non-melanistic 428	*pardus*	Somkhanda Game Reserve, South Africa	-27.7063	31.9791	Montane Grasslands and Shrublands	Drakensberg Montane Woodlands and Grasslands	Photograph	All	no data	Panthera
Non-melanistic 429	*pardus*	Somkhanda Game Reserve, South Africa	-27.7361	31.9650	Montane Grasslands and Shrublands	Drakensberg Montane Woodlands and Grasslands	Photograph	All	no data	Panthera
Non-melanistic 430	*pardus*	Somkhanda Game Reserve, South Africa	-27.8122	31.9274	Montane Grasslands and Shrublands	Drakensberg Montane Woodlands and Grasslands	Photograph	All	no data	Panthera
Non-melanistic 431	*pardus*	Steenkampsberg Mountains, Lydenburg, South Africa	-25.2164	30.5472	Montane Grasslands and Shrublands	Drakensberg Montane Woodlands and Grasslands	Photograph	All	2010	Panthera
Non-melanistic 432	*pardus*	Swartberg Area, South Africa	-30.1610	29.2539	Montane Grasslands and Shrublands	Drakensberg Montane Woodlands and Grasslands	Photograph	All	no data	Wildlife Conservation Society
Non-melanistic 433	*pardus*	Thaba Tholo Wilderness Reserve, South Africa	-24.9169	30.4675	Montane Grasslands and Shrublands	Drakensberg Montane Woodlands and Grasslands	Photograph	All	no data	TTWR
Non-melanistic 434	*pardus*	Thaba Tholo Wilderness Reserve, South Africa	-24.8565	30.5184	Montane Grasslands and Shrublands	Drakensberg Montane Woodlands and Grasslands	Photograph	All	2012	TTWR
Non-melanistic 435	*pardus*	Thaba Tholo Wilderness Reserve, South Africa	-24.8855	30.5195	Montane Grasslands and Shrublands	Drakensberg Montane Woodlands and Grasslands	Photograph	All	2012	TTWR
Non-melanistic 436	*pardus*	Thaba Tholo Wilderness Reserve, South Africa	-24.8700	30.4386	Montane Grasslands and Shrublands	Drakensberg Montane Woodlands and Grasslands	Photograph	All	no data	TTWR
Non-melanistic 437	*pardus*	Thaba Tholo Wilderness Reserve, South Africa	-24.6122	30.4586	Montane Grasslands and Shrublands	Drakensberg Montane Woodlands and Grasslands	Photograph	All	2011	TTWR
Non-melanistic 438	*pardus*	Thaba Tholo Wilderness Reserve, South Africa	-24.6237	30.3404	Tropical and Subtropical Grasslands, Savannas and Shrublands	Southern Africa bushveld	Photograph	All	2012	TTWR
Non-melanistic 439	*pardus*	Thaba Game Reserve, South Africa	-28.4257	30.6239	Montane Grasslands and Shrublands	Drakensberg Montane Woodlands and Grasslands	Photograph	All	no data	Panthera
Non-melanistic 440	*pardus*	Wemmershoek, South Africa	-33.7523	19.2081	Mediterranean Forests, Woodlands, and Scrub	Fynbos	Photograph	All	2011	Cape Leopard Trust
Non-melanistic 441	*pardus*	Wemmershoek, South Africa	-33.7523	19.2081	Mediterranean Forests, Woodlands, and Scrub	Fynbos	Photograph	All	2010	Cape Leopard Trust
Non-melanistic 442	*pardus*	Wemmershoek, South Africa	-33.7537	19.2903	Mediterranean Forests, Woodlands, and Scrub	Fynbos	Photograph	All	2011	Cape Leopard Trust
Non-melanistic 443	*pardus*	Wemmershoek, South Africa	-33.7895	19.2485	Mediterranean Forests, Woodlands, and Scrub	Fynbos	Photograph	All	2011	Cape Leopard Trust
Non-melanistic 444	*pardus*	Wemmershoek Mountains, South Africa	-33.7281	19.3583	Mediterranean Forests, Woodlands, and Scrub	Fynbos	Photograph	All	2010	Cape Leopard Trust
Non-melanistic 445	*kotiya*	Agrapatana, Sri Lanka	6.8015	80.6216	Tropical and Subtropical Moist Broadleaf Forests	Sri Lankan moist forest	Photograph	All	no data	Andrew Kittle
Non-melanistic 446	*kotiya*	Colombo, Western Province, Sri Lanka	6.9373	79.8917	Tropical and Subtropical Moist Broadleaf Forests	Sri Lankan moist forest	Photograph	All	1908	National Museum of Natural History USA
Non-melanistic 447	*kotiya*	Vavuniya Forest, Sri Lanka	8.8836	80.3568	Tropical and Subtropical Dry Broadleaf Forests	Sri Lanka dry-zone dry evergreen forests	Photograph	All	2004	Watson & Kittle 2004
Non-melanistic 448	*kotiya*	Dunumadalawa Forest, Sri Lanka	7.2034	80.6151	Tropical and Subtropical Moist Broadleaf Forests	Sri Lankan moist forest	Photograph	All	no data	Andrew Kittle
Non-melanistic 449	*kotiya*	Hantane, Kandy District, Sri Lanka	7.2709	80.6422	Tropical and Subtropical Moist Broadleaf Forests	Sri Lankan moist forest	Photograph	All	no data	Andrew Kittle
Non-melanistic 450	*kotiya*	Hantane, Kandy District, Sri Lanka	7.2553	80.6325	Tropical and Subtropical Moist Broadleaf Forests	Sri Lankan moist forest	Photograph	All	no data	Andrew Kittle
Non-melanistic 451	*kotiya*	Kantalai, Sri Lanka	8.3076	80.8441	Tropical and Subtropical Dry Broadleaf Forests	Sri Lanka dry-zone dry evergreen forests	Photograph	All	no data	Watson & Kittle 2004
Non-melanistic 452	*kotiya*	Nuwara Eliya, Sri Lanka	7.0260	80.7864	Tropical and Subtropical Moist Broadleaf Forests	Sri Lankan moist forest	Photograph	All	2013	Smithsonian Institution
Non-melanistic 453	*kotiya*	Wasgamuwa National Park, Sri Lanka	7.6389	80.9393	Tropical and Subtropical Dry Broadleaf Forests	Sri Lanka dry-zone dry evergreen forests	Photograph	All	no data	Andrew Kittle
Non-melanistic 454	*kotiya*	Yala National Park, Sri Lanka	6.4632	81.3839	Tropical and Subtropical Dry Broadleaf Forests	Sri Lanka dry-zone dry evergreen forests	Photograph	All	no data	Andrew Kittle
Non-melanistic 455	*kotiya*	Yala National Park, Sri Lanka	6.4632	81.3839	Tropical and Subtropical Dry Broadleaf Forests	Sri Lanka dry-zone dry evergreen forests	Photograph	All	no data	Andrew Kittle
Non-melanistic 456	*kotiya*	Yala National Park, Sri Lanka	6.4644	81.3266	Tropical and Subtropical Dry Broadleaf Forests	Sri Lanka dry-zone dry evergreen forests	Photograph	All	no data	Andrew Kittle
Non-melanistic 457	*kotiya*	Yala National Park, Sri Lanka	6.5198	81.4115	Tropical and Subtropical Dry Broadleaf Forests	Sri Lanka dry-zone dry evergreen forests	Photograph	All	no data	Andrew Kittle
Non-melanistic 458	*kotiya*	Yala National Park, Sri Lanka	6.5303	81.4220	Tropical and Subtropical Dry Broadleaf Forests	Sri Lanka dry-zone dry evergreen forests	Photograph	All	no data	Andrew Kittle
Non-melanistic 459	*kotiya*	Yala National Park, Sri Lanka	6.5079	81.4753	Tropical and Subtropical Dry Broadleaf Forests	Sri Lanka dry-zone dry evergreen forests	Photograph	All	no data	Andrew Kittle
Non-melanistic 460	*kotiya*	Yala National Park, Sri Lanka	6.4714	81.4582	Tropical and Subtropical Dry Broadleaf Forests	Sri Lanka dry-zone dry evergreen forests	Photograph	All	no data	Andrew Kittle
Non-melanistic 461	*kotiya*	Yala National Park, Sri Lanka	6.4813	81.5338	Tropical and Subtropical Dry Broadleaf Forests	Sri Lanka dry-zone dry evergreen forests	Photograph	All	no data	Andrew Kittle
Non-melanistic 462	*kotiya*	Yala National Park, Sri Lanka	6.4686	81.5292	Tropical and Subtropical Dry Broadleaf Forests	Sri Lanka dry-zone dry evergreen forests	Photograph	All	no data	Andrew Kittle
Non-melanistic 463	*kotiya*	Yala National Park, Sri Lanka	6.4785	81.5206	Tropical and Subtropical Dry Broadleaf Forests	Sri Lanka dry-zone dry evergreen forests	Photograph	All	no data	Andrew Kittle
Non-melanistic 464	*kotiya*	Yala National Park, Sri Lanka	6.4112	81.4706	Tropical and Subtropical Dry Broadleaf Forests	Sri Lanka dry-zone dry evergreen forests	Photograph	All	no data	Andrew Kittle
Non-melanistic 465	*kotiya*	Yala National Park, Sri Lanka	6.4077	81.4818	Tropical and Subtropical Dry Broadleaf Forests	Sri Lanka dry-zone dry evergreen forests	Photograph	All	no data	Andrew Kittle
Non-melanistic 466	*kotiya*	Yala National Park, Sri Lanka	6.4231	81.4825	Tropical and Subtropical Dry Broadleaf Forests	Sri Lanka dry-zone dry evergreen forests	Photograph	All	no data	Andrew Kittle
Non-melanistic 467	*kotiya*	Yala National Park, Sri Lanka	6.4239	81.4812	Tropical and Subtropical Dry Broadleaf Forests	Sri Lanka dry-zone dry evergreen forests	Photograph	All	no data	Andrew Kittle
Non-melanistic 468	*kotiya*	Yala National Park, Sri Lanka	6.4364	81.5556	Tropical and Subtropical Dry Broadleaf Forests	Sri Lanka dry-zone dry evergreen forests	Photograph	All	no data	Andrew Kittle
Non-melanistic 469	*kotiya*	Yala National Park, Sri Lanka	6.4077	81.5530	Tropical and Subtropical Dry Broadleaf Forests	Sri Lanka dry-zone dry evergreen forests	Photograph	All	no data	Andrew Kittle
Non-melanistic 470	*pardus*	El Dueim, Ash Shamaliyah, Sudan	14.0298	32.2618	Tropical and Subtropical Grasslands, Savannas and Shrublands	Sahelian Acacia savanna	Photograph	All	1910	National Museum of Natural History USA
Non-melanistic 471	*pardus*	Nimule National Park, Sudan	3.6133	32.1361	Tropical and Subtropical Grasslands, Savannas and Shrublands	East Sudanian savanna	Photograph	All	1962	American Museum of Natural History USA
Non-melanistic 472	*pardus*	Serengeti Plains, Tanzania	-2.1623	34.4294	Tropical and Subtropical Grasslands, Savannas and Shrublands	East African Acacia Savannas	Photograph	All	no data	American Museum of Natural History USA
Non-melanistic 473	*pardus*	Tanganyika, Rungwe, Tanzania	-5.4804	29.9287	Tropical and Subtropical Grasslands, Savannas and Shrublands	Central Zambezian Miombo woodlands	Photograph	All	1929	American Museum of Natural History USA
Non-melanistic 474	*pardus*	Tanganyika, Rungwe, Tanzania	-5.7615	30.3734	Tropical and Subtropical Grasslands, Savannas and Shrublands	Central Zambezian Miombo woodlands	Photograph	All	1929	American Museum of Natural History USA
Non-melanistic 475	*pardus*	Tanganyika, Rungwe, Tanzania	-6.1077	30.4335	Tropical and Subtropical Grasslands, Savannas and Shrublands	Central Zambezian Miombo woodlands	Photograph	All	1929	American Museum of Natural History USA
Non-melanistic 476	*pardus*	Serengeti National Park, Tanzania	-2.5763	34.3123	Tropical and Subtropical Grasslands, Savannas and Shrublands	East African Acacia Savannas	Photograph	All	no data	National Geographic Society
Non-melanistic 477	*pardus*	Serengeti National Park, Tanzania	-2.4335	34.5247	Tropical and Subtropical Grasslands, Savannas and Shrublands	East African Acacia Savannas	Photograph	All	no data	National Geographic Society

Id	Subspecies	Location	Deg. - WGS84		Biome	Ecorregion	Sample	Analysis	Year	Source
			Latitude	Longitude						
Non-melanistic 478	*pardus*	Udzungwa Benjamin Drummond Mountains, Tanzania	-7.7762	36.6822	Tropical and Subtropical Moist Broadleaf Forests	Eastern Arc Montane Forests	Photograph	All	2011	Wildlife Conservation Society
Non-melanistic 479	*pardus*	Zanzibar, Tanzania	-6.0133	39.2655	Tropical and Subtropical Moist Broadleaf Forests	Northern Zanzibar-Inhambane coastal forest mosaic	Photograph	Filtered	1923	Zanzibar Museum
Non-melanistic 480	*pardus*	Zanzibar, Tanzania	-5.9007	39.3223	Tropical and Subtropical Moist Broadleaf Forests	Northern Zanzibar-Inhambane coastal forest mosaic	Photograph	Filtered	1924	Zanzibar Museum
Non-melanistic 481	*pardus*	Zanzibar, Tanzania	-5.9603	39.3396	Tropical and Subtropical Moist Broadleaf Forests	Northern Zanzibar-Inhambane coastal forest mosaic	Photograph	Filtered	1924	Zanzibar Museum
Non-melanistic 482	*delacouri*	Huai Kha Khaeng Wildlife Sanctuary, Thailand	15.8022	99.7023	Tropical and Subtropical Dry Broadleaf Forests	Indochina dry forests	Photograph	All	2009	Kae Kawanishi
Non-melanistic 483	*delacouri*	Huai Kha Khaeng Wildlife Sanctuary, Thailand	15.4968	99.6261	Tropical and Subtropical Dry Broadleaf Forests	Indochina dry forests	Photograph	All	2009	Kae Kawanishi
Non-melanistic 484	*delacouri*	Huai Kha Khaeng Wildlife Sanctuary, Thailand	15.2545	98.7483	Tropical and Subtropical Moist Broadleaf Forests	Kayah-Karen/Tenasserim moist forests	Photograph	All	2012	Bruce Kekule
Non-melanistic 485	*delacouri*	Huai Kha Khaeng Wildlife Sanctuary, Thailand	14.7850	99.2517	Tropical and Subtropical Moist Broadleaf Forests	Kayah-Karen/Tenasserim moist forests	Photograph	All	2012	Bruce Kekule
Non-melanistic 486	*delacouri*	Huai Kha Khaeng Wildlife Sanctuary, Thailand	14.7850	99.2517	Tropical and Subtropical Moist Broadleaf Forests	Kayah-Karen/Tenasserim moist forests	Photograph	All	no data	Bruce Kekule
Non-melanistic 487	*delacouri*	Huai Kha Khaeng Wildlife Sanctuary, Thailand	15.5370	99.1422	Tropical and Subtropical Moist Broadleaf Forests	Kayah-Karen/Tenasserim moist forests	Photograph	All	2013	Bruce Kekule
Non-melanistic 488	*delacouri*	Huai Kha Khaeng Wildlife Sanctuary, Thailand	16.0221	98.6581	Tropical and Subtropical Moist Broadleaf Forests	Kayah-Karen/Tenasserim moist forests	Photograph	All	2013	Bruce Kekule
Non-melanistic 489	*delacouri*	Huai Kha Khaeng Wildlife Sanctuary, Thailand	15.5751	99.1429	Tropical and Subtropical Moist Broadleaf Forests	Kayah-Karen/Tenasserim moist forests	Photograph	All	2012	Wildlife Conservation Society
Non-melanistic 490	*delacouri*	Huai Kha Khaeng Wildlife Sanctuary, Thailand	15.0925	98.9792	Tropical and Subtropical Moist Broadleaf Forests	Kayah-Karen/Tenasserim moist forests	Photograph	All	2012	Wildlife Conservation Society
Non-melanistic 491	*delacouri*	Huai Kha Khaeng Wildlife Sanctuary, Thailand	14.7990	98.9247	Tropical and Subtropical Moist Broadleaf Forests	Kayah-Karen/Tenasserim moist forests	Photograph	All	2012	Wildlife Conservation Society
Non-melanistic 492	*delacouri*	Huai Kha Khaeng Wildlife Sanctuary, Thailand	14.8046	98.5320	Tropical and Subtropical Moist Broadleaf Forests	Kayah-Karen/Tenasserim moist forests	Photograph	All	2013	Wildlife Conservation Society
Non-melanistic 493	*delacouri*	Huai Kha Khaeng Wildlife Sanctuary, Thailand	14.3872	99.2542	Tropical and Subtropical Moist Broadleaf Forests	Kayah-Karen/Tenasserim moist forests	Photograph	All	2013	Wildlife Conservation Society
Non-melanistic 494	*delacouri*	Kaeng Krachan National Park, Thailand	12.8847	99.6302	Tropical and Subtropical Moist Broadleaf Forests	Kayah-Karen/Tenasserim moist forests	Photograph	All	no data	Bruce Kekule
Non-melanistic 495	*delacouri*	Kaeng Krachan National Park, Thailand	12.0023	99.6938	Tropical and Subtropical Moist Broadleaf Forests	Kayah-Karen/Tenasserim moist forests	Photograph	All	no data	Bruce Kekule
Non-melanistic 496	*delacouri*	Kaeng Krachan National Park, Thailand	12.9500	99.2334	Tropical and Subtropical Moist Broadleaf Forests	Kayah-Karen/Tenasserim moist forests	Photograph	All	no data	Bruce Kekule
Non-melanistic 497	*delacouri*	Kaeng Krachan National Park, Thailand	12.9500	99.2334	Tropical and Subtropical Moist Broadleaf Forests	Kayah-Karen/Tenasserim moist forests	Photograph	All	2009	Bruce Kekule
Non-melanistic 498	*delacouri*	Kaeng Krachan National Park, Thailand	13.0527	99.1907	Tropical and Subtropical Moist Broadleaf Forests	Kayah-Karen/Tenasserim moist forests	Photograph	All	2009	Bruce Kekule
Non-melanistic 499	*delacouri*	Kaeng Krachan National Park, Thailand	12.9500	99.2334	Tropical and Subtropical Moist Broadleaf Forests	Kayah-Karen/Tenasserim moist forests	Photograph	All	no data	Bruce Kekule
Non-melanistic 500	*delacouri*	Kaeng Krachan National Park, Thailand	13.3983	99.5686	Tropical and Subtropical Moist Broadleaf Forests	Kayah-Karen/Tenasserim moist forests	Photograph	All	2012	Bruce Kekule
Non-melanistic 501	*delacouri*	Kanchanaburi, Thailand	14.0507	99.5436	Tropical and Subtropical Moist Broadleaf Forests	Kayah-Karen/Tenasserim moist forests	Photograph	All	no data	Allwen Jesudasan
Non-melanistic 502	*delacouri*	Malay Peninsula, Thailand	13.1955	99.2677	Tropical and Subtropical Moist Broadleaf Forests	Kayah-Karen/Tenasserim moist forests	Photograph	All	2009	Kae Kawanishi
Non-melanistic 503	*delacouri*	Malay Peninsula, Thailand	12.9322	99.3147	Tropical and Subtropical Moist Broadleaf Forests	Kayah-Karen/Tenasserim moist forests	Photograph	All	2009	Kae Kawanishi
Non-melanistic 504	*saxicolor*	Sukavuşumu, Yusufeli County, Turkey	41.3611	42.1203	Temperate Broadleaf and Mixed Forests	Caucasus mixed forests	Photograph	All	1999	Igor Khorozian
Non-melanistic 505	*pardus*	Masindi, Uganda	1.7148	31.6919	Tropical and Subtropical Grasslands, Savannas and Shrublands	Victoria Basin forest-savanna mosaic	Photograph	All	1920	National Museum of Natural History USA
Non-melanistic 506	*pardus*	Murchison Falls National Park, Uganda	2.1606	31.5837	Tropical and Subtropical Grasslands, Savannas and Shrublands	Victoria Basin forest-savanna mosaic	Photograph	All	no data	Smithsonian Institution
Non-melanistic 507	*pardus*	Queen Elizabeth National Park, Uganda	-0.2880	29.9489	Tropical and Subtropical Grasslands, Savannas and Shrublands	Victoria Basin forest-savanna mosaic	Photograph	All	no data	Smithsonian Institution
Non-melanistic 508	*delacouri*	Plateau Du Kontum, Gia Lai-Kon Tum, Vietnam	13.9174	108.1171	Tropical and Subtropical Dry Broadleaf Forests	Indochina dry forests	Photograph	All	1963	National Museum of Natural History USA
Non-melanistic 509	*nimr*	Hawf Protected Area, Yemen	16.6663	44.4431	Deserts and Xeric Shrublands	Arabian Highlands woodlands and shrublands	Photograph	All	no data	David Stanton
Non-melanistic 510	*nimr*	Hawf Protected Area, Yemen	16.2698	43.2391	Deserts and Xeric Shrublands	Arabian Highlands woodlands and shrublands	Photograph	All	2011	David Stanton
Non-melanistic 511	*nimr*	Hawf Protected Area, Yemen	16.7597	44.1937	Deserts and Xeric Shrublands	Arabian Highlands woodlands and shrublands	Photograph	All	2011	David Stanton
Non-melanistic 512	*nimr*	Wada'i, Yemen	16.0167	43.8932	Deserts and Xeric Shrublands	Arabian Highlands woodlands and shrublands	Photograph	All	no data	Jane Budd
Non-melanistic 513	*nimr*	Wada'i Boundaries, Yemen	15.8774	43.6560	Deserts and Xeric Shrublands	Arabian Highlands woodlands and shrublands	Photograph	All	2002	Jane Budd
Non-melanistic 514	*pardus*	Akenge, Congo	-4.8484	21.9107	Tropical and Subtropical Grasslands, Savannas and Shrublands	Southern Congolian forest-savanna mosaic	Photograph	All	1913	American Museum of Natural History USA
Non-melanistic 515	*pardus*	Akenge, Congo	-4.6869	21.9299	Tropical and Subtropical Grasslands, Savannas and Shrublands	Southern Congolian forest-savanna mosaic	Photograph	All	1913	American Museum of Natural History USA
Non-melanistic 516	*pardus*	Bafuka, Haut, Congo	3.7467	28.6836	Tropical and Subtropical Grasslands, Savannas and Shrublands	Northern Congolian forest-savanna mosaic	Photograph	All	1911	American Museum of Natural History USA
Non-melanistic 517	*pardus*	Faradje, Haut, Congo	3.7022	29.7332	Tropical and Subtropical Grasslands, Savannas and Shrublands	Northern Congolian forest-savanna mosaic	Photograph	All	1911	American Museum of Natural History USA
Non-melanistic 518	*pardus*	Faradje, Haut, Congo	3.7022	29.7332	Tropical and Subtropical Grasslands, Savannas and Shrublands	Northern Congolian forest-savanna mosaic	Photograph	All	1911	American Museum of Natural History USA
Non-melanistic 519	*pardus*	Faradje, Haut, Congo	3.9682	29.6295	Tropical and Subtropical Grasslands, Savannas and Shrublands	Northern Congolian forest-savanna mosaic	Photograph	All	1911	American Museum of Natural History USA
Non-melanistic 520	*pardus*	Faradje, Haut, Congo	3.8220	29.5090	Tropical and Subtropical Grasslands, Savannas and Shrublands	Northern Congolian forest-savanna mosaic	Photograph	All	1911	American Museum of Natural History USA
Non-melanistic 521	*pardus*	Faradje, Haut, Congo	3.8220	29.5090	Tropical and Subtropical Grasslands, Savannas and Shrublands	Northern Congolian forest-savanna mosaic	Photograph	All	1911	American Museum of Natural History USA
Non-melanistic 522	*pardus*	Faradje, Haut, Congo	3.8220	29.5090	Tropical and Subtropical Grasslands, Savannas and Shrublands	Northern Congolian forest-savanna mosaic	Photograph	All	1911	American Museum of Natural History USA
Non-melanistic 523	*pardus*	Faradje, Haut, Congo	3.8032	29.9086	Tropical and Subtropical Grasslands, Savannas and Shrublands	Northern Congolian forest-savanna mosaic	Photograph	All	1911	American Museum of Natural History USA
Non-melanistic 524	*pardus*	Faradje, Haut, Congo	3.7623	29.9336	Tropical and Subtropical Grasslands, Savannas and Shrublands	Northern Congolian forest-savanna mosaic	Photograph	All	1911	American Museum of Natural History USA
Non-melanistic 525	*pardus*	Faradje, Haut, Congo	3.6523	29.8528	Tropical and Subtropical Grasslands, Savannas and Shrublands	Northern Congolian forest-savanna mosaic	Photograph	All	1911	American Museum of Natural History USA
Non-melanistic 526	*pardus*	Faradje, Haut, Congo	3.6523	29.8528	Tropical and Subtropical Grasslands, Savannas and Shrublands	Northern Congolian forest-savanna mosaic	Photograph	All	1911	American Museum of Natural History USA
Non-melanistic 527	*pardus*	Faradje, Haut, Congo	3.8000	29.6956	Tropical and Subtropical Grasslands, Savannas and Shrublands	Northern Congolian forest-savanna mosaic	Photograph	All	1911	American Museum of Natural History USA
Non-melanistic 528	*pardus*	Faradje, Haut, Congo	3.6790	29.6251	Tropical and Subtropical Grasslands, Savannas and Shrublands	Northern Congolian forest-savanna mosaic	Photograph	All	1911	American Museum of Natural History USA
Non-melanistic 529	*pardus*	Faradje, Haut, Congo	3.6790	29.6251	Tropical and Subtropical Grasslands, Savannas and Shrublands	Northern Congolian forest-savanna mosaic	Photograph	All	1911	American Museum of Natural History USA
Non-melanistic 530	*pardus*	Faradje, Haut, Congo	3.6271	29.6647	Tropical and Subtropical Grasslands, Savannas and Shrublands	Northern Congolian forest-savanna mosaic	Photograph	All	1911	American Museum of Natural History USA
Non-melanistic 531	*pardus*	Faradje, Haut, Congo	3.6271	29.6647	Tropical and Subtropical Grasslands, Savannas and Shrublands	Northern Congolian forest-savanna mosaic	Photograph	All	1911	American Museum of Natural History USA
Non-melanistic 532	*pardus*	Faradje, Haut, Congo	3.6271	29.6647	Tropical and Subtropical Grasslands, Savannas and Shrublands	Northern Congolian forest-savanna mosaic	Photograph	All	1911	American Museum of Natural History USA
Non-melanistic 533	*pardus*	Gamangui, Congo	3.6631	27.3678	Tropical and Subtropical Grasslands, Savannas and Shrublands	Northern Congolian forest-savanna mosaic	Photograph	All	1913	American Museum of Natural History USA
Non-melanistic 534	*pardus*	Madge, Congo	3.5396	27.0286	Tropical and Subtropical Grasslands, Savannas and Shrublands	Northern Congolian forest-savanna mosaic	Photograph	All	1914	American Museum of Natural History USA
Non-melanistic 535	*pardus*	Niapu, Congo	2.4361	26.4438	Tropical and Subtropical Moist Broadleaf Forests	Northeastern Congo Basin moist forests	Photograph	All	1913	American Museum of Natural History USA
Non-melanistic 536	*pardus*	Niapu, Congo	2.3027	26.4826	Tropical and Subtropical Moist Broadleaf Forests	Northeastern Congo Basin moist forests	Photograph	All	1913	American Museum of Natural History USA
Non-melanistic 537	*pardus*	Niapu, Congo	2.3001	26.2433	Tropical and Subtropical Moist Broadleaf Forests	Northeastern Congo Basin moist forests	Photograph	All	1913	American Museum of Natural History USA
Non-melanistic 538	*pardus*	Niapu, Congo	2.4914	26.3490	Tropical and Subtropical Moist Broadleaf Forests	Northeastern Congo Basin moist forests	Photograph	All	1913	American Museum of Natural History USA
Non-melanistic 539	*pardus*	Poko, Garamba, Congo	3.1303	26.9121	Tropical and Subtropical Grasslands, Savannas and Shrublands	Northern Congolian forest-savanna mosaic	Photograph	All	1913	American Museum of Natural History USA
Non-melanistic 540	*pardus*	Vankerckhovenville, Haut, Congo	3.0746	30.3358	Tropical and Subtropical Grasslands, Savannas and Shrublands	Northern Congolian forest-savanna mosaic	Photograph	All	1911	American Museum of Natural History USA
Non-melanistic 541	*pardus*	Chobe National Park, Zambia	-17.6599	25.2459	Tropical and Subtropical Grasslands, Savannas and Shrublands	Southern Miombo woodlands	Photograph	All	no data	Chobe Safari Lodge
Non-melanistic 542	*pardus*	Luangwa, Zambia	-15.6191	30.2616	Tropical and Subtropical Grasslands, Savannas and Shrublands	Zambezian and Mopane woodlands	Photograph	All	no data	Project Luangwa

Id	Subspecies	Location	Deg. - WGS84		Biome	Ecorregion	Sample	Analysis	Year	Source
			Latitude	Longitude						
Non-melanistic 543	*pardus*	Luangwa, Zambia	-15.5797	30.3073	Tropical and Subtropical Grasslands, Savannas and Shrublands	Zambezian and Mopane woodlands	Photograph	All	no data	Project Luangwa
Non-melanistic 544	*pardus*	Luangwa, Zambia	-15.4602	30.2923	Tropical and Subtropical Grasslands, Savannas and Shrublands	Zambezian and Mopane woodlands	Photograph	All	no data	Project Luangwa
Non-melanistic 545	*pardus*	Luangwa, Zambia	-15.4425	30.1700	Tropical and Subtropical Grasslands, Savannas and Shrublands	Zambezian and Mopane woodlands	Photograph	All	no data	Project Luangwa
Non-melanistic 546	*pardus*	South Luangwa National Park, Zambia	-12.5524	31.7476	Tropical and Subtropical Grasslands, Savannas and Shrublands	Zambezian and Mopane woodlands	Photograph	All	no data	Bushcamp Project
Non-melanistic 547	*pardus*	South Luangwa National Park, Zambia	-12.6344	31.7317	Tropical and Subtropical Grasslands, Savannas and Shrublands	Zambezian and Mopane woodlands	Photograph	All	no data	Andrew Stein
Non-melanistic 548	*pardus*	Mashonaland, Zimbabwe	-16.5912	31.0548	Tropical and Subtropical Grasslands, Savannas and Shrublands	Southern Miombo woodlands	Photograph	All	1892	National Museum of Natural History USA
Non-melanistic 549	*pardus*	Ruaha National Park, Tanzania	-7.5826	34.3244	Tropical and Subtropical Grasslands, Savannas and Shrublands	Central Zambezian Miombo woodlands	Photograph	All	2012	WildCru
Non-melanistic 550	*pardus*	Welgevonden Game Reserve, South Africa	-24.3418	28.0210	Tropical and Subtropical Grasslands, Savannas and Shrublands	Southern Africa bushveld	Photograph	All	2011	Panthera
Non-melanistic 551	*pardus*	Welgevonden Game Reserve, South Africa	-24.3418	28.0210	Tropical and Subtropical Grasslands, Savannas and Shrublands	Southern Africa bushveld	Photograph	All	2011	Panthera
Non-melanistic 552	*orientalis*	Wangqing, China	43.0140	130.3603	Temperate Broadleaf and Mixed Forests	Manchurian mixed forests	Photograph	All	2010	World Wild Fund
