# Supplementary material for: Mapping black panthers: Macroecological modeling of melanism in leopards (Panthera pardus)
Source: PLoS One. 2017 Apr 5;12(4):e0170378. doi: 10.1371/journal.pone.0170378 (PMC5381760; doi:10.1371/journal.pone.0170378)
Supplement: S2 Table — (PDF) [file pone.0170378.s002.pdf]

S2 Table - Environmental predictors used in the initial analysis and selected with Pearson's test (in red).

Code	Environmental predictor
Land	Landscape conformation (NDVI+GlobCover)
**Alt**	**Altitude**
**Bio01**	**Annual mean temperature**
Bio02	Mean diurnal temperature range (mean(period max-min))
Bio03	Isothermality (Bio02 ÷ Bio07)
Bio04	Temperature seasonality
**Bio05**	**Max temperature of warmest week**
**Bio06**	**Min temperature of coldest week**
Bio07	Temperature annual range (Bio05-Bio06)
Bio08	Mean temperature of wettest quarter
Bio09	Mean temperature of driest quarter
Bio10	Mean temperature of warmest quarter
Bio11	Mean temperature of coldest quarter
**Bio12**	**Annual precipitation**
**Bio13**	**Precipitation of wettest week**
Bio14	Precipitation of driest week
**Bio15**	**Precipitation seasonality**
Bio16	Precipitation of wettest quarter
Bio17	Precipitation of driest quarter
Bio18	Precipitation of warmest quarter
Bio19	Precipitation of coldest quarter
**Bio20**	**Annual mean radiation**
Bio21	Highest weekly radiation
Bio22	Lowest weekly radiation
**Bio23**	**Radiation seasonality**
Bio24	Radiation of wettest quarter
Bio25	Radiation of driest quarter
Bio26	Radiation of warmest quarter
Bio27	Radiation of coldest quarter
Bio28	Annual mean moisture index
**Bio29**	**Highest weekly moisture index**
Bio30	Lowest weekly moisture index
Bio31	Moisture index seasonality
**Bio32**	**Mean moisture index of wettest quarter**
**Bio33**	**Mean moisture index of driest quarter**
Bio34	Mean moisture index of warmest quarter
Bio35	Mean moisture index of coldest quarter
