# Supplementary figures and images for: Mapping black panthers: Macroecological modeling of melanism in leopards (Panthera pardus)
Source: PLoS One. 2017 Apr 5;12(4):e0170378. doi: 10.1371/journal.pone.0170378 (PMC5381760; doi:10.1371/journal.pone.0170378)

S1 Fig – Base map for the CSR test.

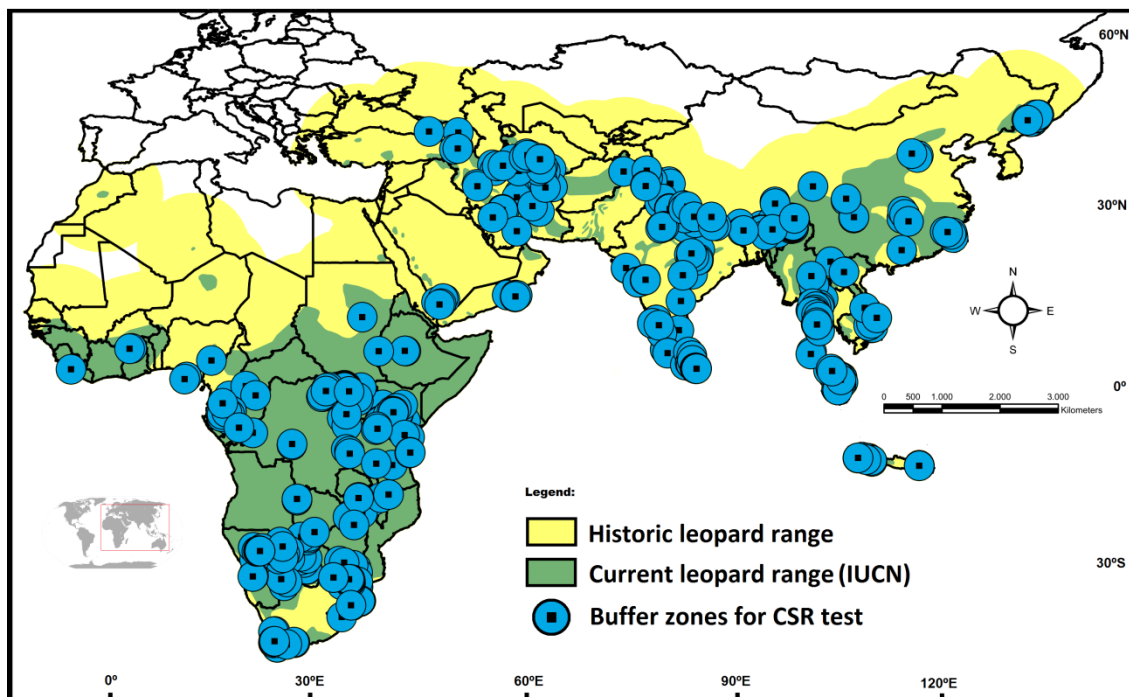

Supplement: S1 Fig — (PDF) [file pone.0170378.s003.pdf]
