# Supplementary material for: Mapping black panthers: Macroecological modeling of melanism in leopards (Panthera pardus)
Source: PLoS One. 2017 Apr 5;12(4):e0170378. doi: 10.1371/journal.pone.0170378 (PMC5381760; doi:10.1371/journal.pone.0170378)

S2 Fig - Response curves observed in the Maxent analysis for each environmental predictor used to construct the non-melanistic (control) model.

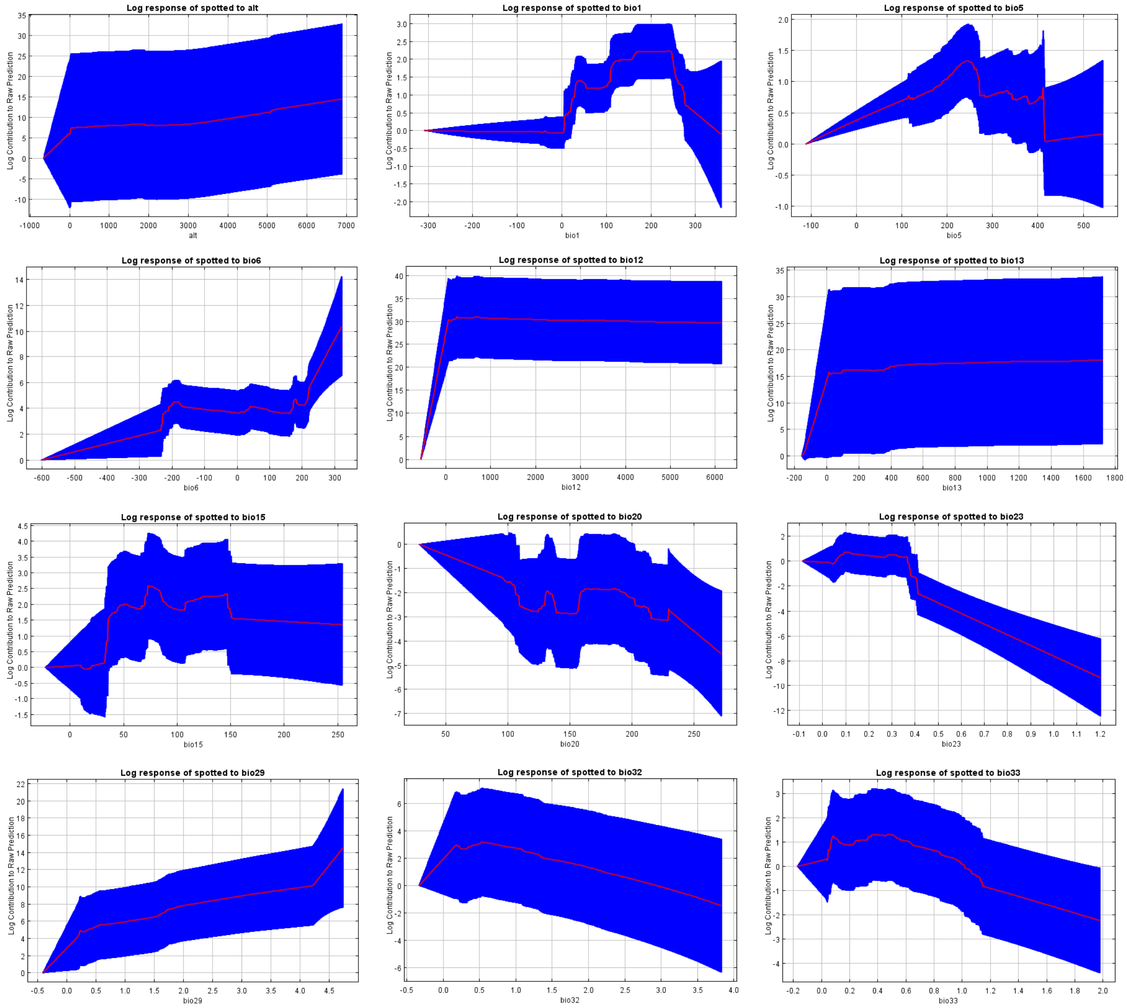

Supplement: S2 Fig — (PDF) [file pone.0170378.s004.pdf]
