# Supplementary material for: Mapping black panthers: Macroecological modeling of melanism in leopards (Panthera pardus)
Source: PLoS One. 2017 Apr 5;12(4):e0170378. doi: 10.1371/journal.pone.0170378 (PMC5381760; doi:10.1371/journal.pone.0170378)

S4 Fig - New distributional map for *Panthera pardus*. Location records comprising our full database are indicated, and overlaid on the present IUCN range map along with additional areas of occurrence documented in this study. Subspecies partitions proposed by Uphyrkina et al. (2001) are also indicated, including summaries of the number records of each coloration phenotype per subspecies.

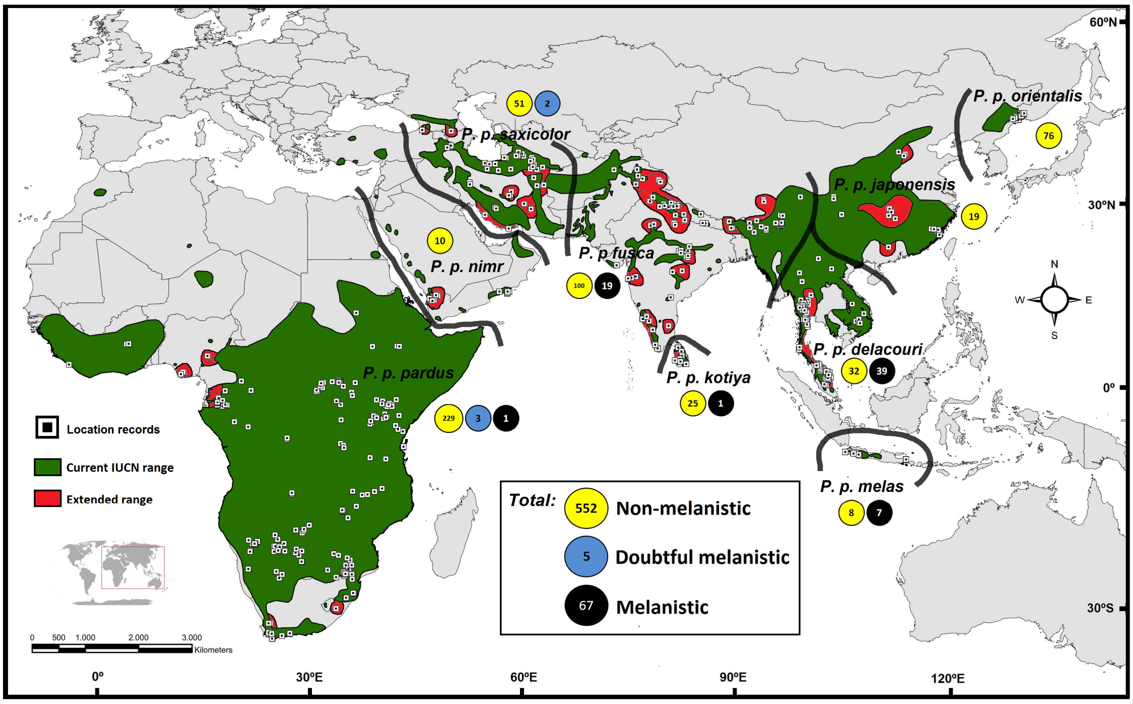

Supplement: S4 Fig — Location records comprising our full database are indicated, and overlaid on the present IUCN range map along with additional areas of occurrence documented in this study. Subspecies partitions proposed by Uphyrkina et al. (2001) are also indicated, including summaries of the number records of each coloration phenotype per subspecies. (PDF) [file pone.0170378.s006.pdf]
