# Supplementary material for: Mapping black panthers: Macroecological modeling of melanism in leopards (Panthera pardus)
Source: PLoS One. 2017 Apr 5;12(4):e0170378. doi: 10.1371/journal.pone.0170378 (PMC5381760; doi:10.1371/journal.pone.0170378)

S5 Fig. – Detailed maps showing the geographic distribution of records comprising our database of melanistic and non-melanistic leopards, overlaid on the terrestrial biomes (based on Olson et al. 2001). Each major geographic region representing a leopard subspecies is shown in a separate panel.

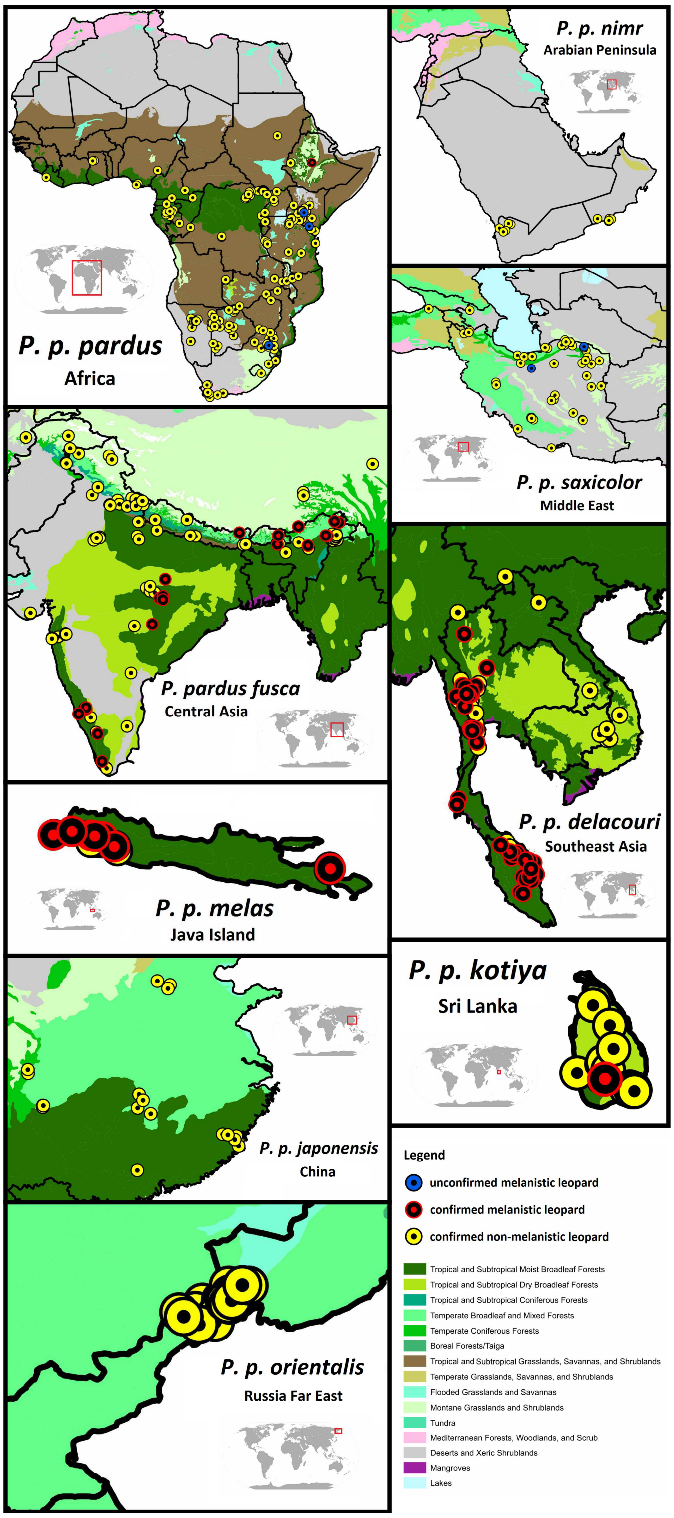

Supplement: S5 Fig — Each major geographic region representing a leopard subspecies is shown in a separate panel. (PDF) [file pone.0170378.s007.pdf]
