# Supplementary material for: Mapping black panthers: Macroecological modeling of melanism in leopards (Panthera pardus)
Source: PLoS One. 2017 Apr 5;12(4):e0170378. doi: 10.1371/journal.pone.0170378 (PMC5381760; doi:10.1371/journal.pone.0170378)

S6 Fig – Detailed assessment of the 12 bioclimatic predictors selected for inclusion in the Maxent modeling of the two coloration phenotypes, after removing the variables showing the most correlation relative to all others (see Methods). A) Relative importance of each variable for modeling habitat suitability, depicted separately for three geographic scales: (i) full data set (‘Non-melanistic’ and ‘melanistic’ in the legend); (ii) Central+Southeastern Asia [*P. p. fusca*, *P. p. melas*, *P. p. delacouri*, *P. p. japonensis* and *P. p. kotiya* in S5 Fig] (‘Non-melanistic Asia’ and ‘melanistic Asia’ in the legend); (iii) (ii) Southeast Asia [*P. p. delacouri* in S5 Fig] (‘Non-melanistic SE Asia’ and ‘melanistic SE Asia’ in the legend). B) Mean absolute values of each of the 12 selected variables for the same six groups (phenotypes *vs.* geographic scales) depicted on panel A. Arrows indicate two variables (‘Annual precipitation’ and ‘Mean moisture index of driest quarter’) that exhibited a consistent pattern of differential effects on the two phenotype-based models, across the three assessed geographic scales.

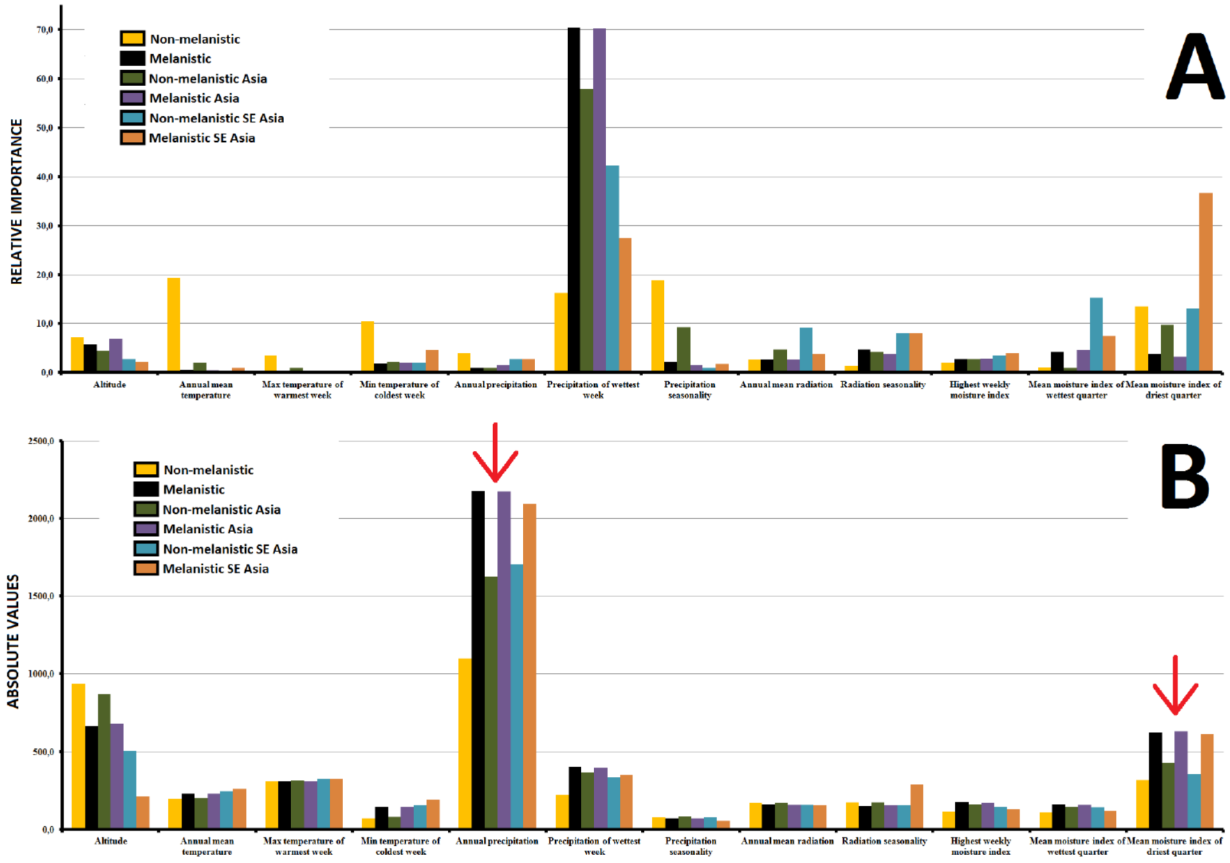

Supplement: S6 Fig — A) Relative importance of each variable for modeling habitat suitability, depicted separately for three geographic scales: (i) full data set (‘Non-melanistic’ and ‘melanistic’ in the legend); (ii) Central+Southeastern Asia [P. p. fusca, P. p. melas, P. p. delacouri, P. p. japonensis and P. p. kotiya in S5 Fig] (‘Non-melanistic Asia’ and ‘melanistic Asia’ in the legend); (iii) (ii) Southeast Asia [P. p. delacouri in S5 Fig] (‘Non-melanistic SE Asia’ and ‘melanistic SE Asia’ in the legend). B) Mean absolute values of each of the 12 selected variables for the same six groups (phenotypes vs. geographic scales) depicted on panel A. Arrows indicate two variables (‘Annual precipitation’ and ‘Mean moisture index of driest quarter’) that exhibited a consistent pattern of differential effects on the two phenotype-based models, across the three assessed geographic scales. (PDF) [file pone.0170378.s008.pdf]
