# Supplementary material for: Mapping black panthers: Macroecological modeling of melanism in leopards (Panthera pardus)
Source: PLoS One. 2017 Apr 5;12(4):e0170378. doi: 10.1371/journal.pone.0170378 (PMC5381760; doi:10.1371/journal.pone.0170378)

S7 Fig – Graphs depicting the results of the suitability test comparing the melanistic and non-melanistic models across all the location records in our database ($p<0.001$). Mean suitability in the non-melanistic model = 0.594 (standard deviation=0.167); mean suitability in the melanistic model = 0.192 (standard deviation=0.280).

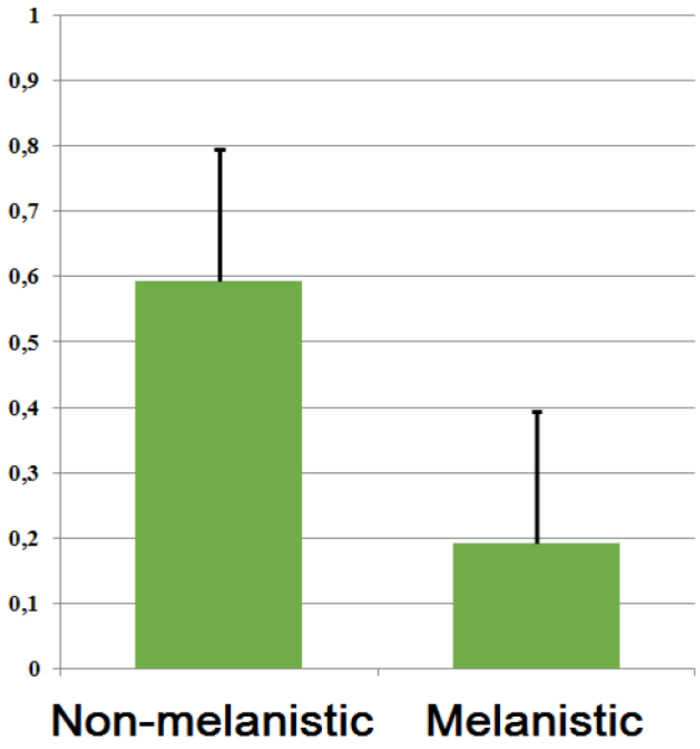

Supplement: S7 Fig — Mean suitability in the non-melanistic model = 0.594 (standard deviation = 0.167); mean suitability in the melanistic model = 0.192 (standard deviation = 0.280). (PDF) [file pone.0170378.s009.pdf]
