# Supplementary material for: Mapping black panthers: Macroecological modeling of melanism in leopards (Panthera pardus)
Source: PLoS One. 2017 Apr 5;12(4):e0170378. doi: 10.1371/journal.pone.0170378 (PMC5381760; doi:10.1371/journal.pone.0170378)

S8 Fig – In-depth analysis of environmental variables and their relationship with melanism in leopards. The top three graphs (panel A) depict the relationships between the two variables identified as having differential effects on the two phenotypes (see S6 Fig), as well as their relationship with a measure of cover (vegetation index NDVI). For each graph, the relationship between the assessed variables is shown for three different geographic scales (as defined in S6 Fig). Panels B-D depict the relationship between each of these three explanatory variables and the habitat suitability estimated for melanistic and non-melanistic leopards, shown separately for the same three geographic scales.

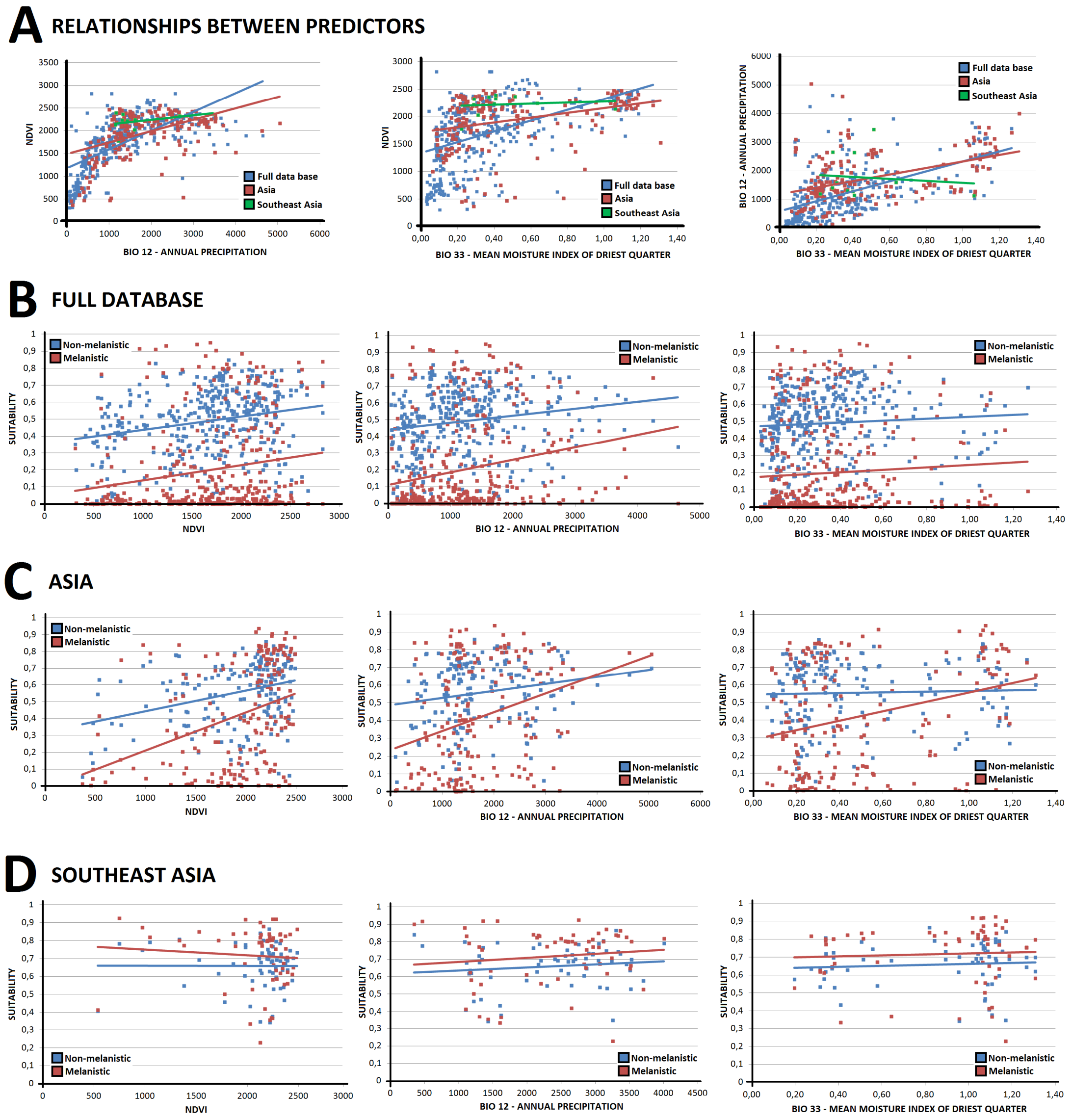

Supplement: S8 Fig — The top three graphs (panel A) depict the relationships between the two variables identified as having differential effects on the two phenotypes (see S6 Fig), as well as their relationship with a measure of cover (vegetation index NDVI). For each graph, the relationship between the assessed variables is shown for three different geographic scales (as defined in S6 Fig). Panels B-D depict the relationship between each of these three explanatory variables and the habitat suitability estimated for melanistic and non-melanistic leopards, shown separately for the same three geographic scales. (PDF) [file pone.0170378.s010.pdf]
